# Novel genes dramatically alter regulatory network topology in amphioxus

**DOI:** 10.1186/gb-2008-9-8-r123

**Published:** 2008-08-04

**Authors:** Qing Zhang, Christian M Zmasek, Larry J Dishaw, M Gail Mueller, Yuzhen Ye, Gary W Litman, Adam Godzik

**Affiliations:** 1 Burnham Institute for Medical Research, North Torrey Pines Road, La Jolla, CA 92037, USA; 2 Department of Molecular Genetics, All Children's Hospital, 6th Street South, St. Petersburg, FL 33701, USA; 3 H Lee Moffitt Cancer Center and Research Institute, Magnolia Drive, Tampa, FL 33612, USA; 4 School of Informatics, Indiana University, E. 10th Street, Bloomington, IN 47408, USA; 5 Department of Pediatrics, University of South Florida, Children's Research Institute, First Street South, St. Petersburg, FL 33701, USA; 6 Skaggs School of Pharmacy and Pharmaceutical Sciences, University of California, San Diego, Gilman Drive, La Jolla, CA 92093, USA

## Abstract

**Background:**

Regulation in protein networks often utilizes specialized domains that 'join' (or 'connect') the network through specific protein-protein interactions. The innate immune system, which provides a first and, in many species, the only line of defense against microbial and viral pathogens, is regulated in this way. Amphioxus (*Branchiostoma floridae*), whose genome was recently sequenced, occupies a unique position in the evolution of innate immunity, having diverged within the chordate lineage prior to the emergence of the adaptive immune system in vertebrates.

**Results:**

The repertoire of several families of innate immunity proteins is expanded in amphioxus compared to both vertebrates and protostome invertebrates. Part of this expansion consists of genes encoding proteins with unusual domain architectures, which often contain both upstream receptor and downstream activator domains, suggesting a potential role for direct connections (shortcuts) that bypass usual signal transduction pathways.

**Conclusion:**

Domain rearrangements can potentially alter the topology of protein-protein interaction (and regulatory) networks. The extent of such arrangements in the innate immune network of amphioxus suggests that domain shuffling, which is an important mechanism in the evolution of multidomain proteins, has also shaped the development of immune systems.

## Abstract 2

Domain rearrangements in the innate immune network of amphioxus suggests that domain shuffling has shaped the evolution of immune systems.

## Background

Protein networks are often 'joined' (or 'connected') by specialized protein-protein interaction domains that specifically recognize their targets and thus connect upstream and downstream elements of the network. The group of proteins involved in apoptosis, members of which incorporate the death domain (DD), death effector domain (DED), and caspase recruitment domain (CARD) [1], and the group of proteins involved in innate immunity, members of which incorporate the Toll/interleukin-1 receptor (TIR) domains [2,3], represent excellent examples of such networks. Genomes of extensively studied organisms, such as *Caenorhabditis elegans*, *Drosophila melanogaster*, and human, display strong conservation of many elements of these two networks. In genome evolution, domain recombination events, such as fusion and fission, can create proteins with novel domain combinations that may lead to new functions, including providing new connections inside an existing network or between different networks [4,5]. Traditionally, it was generally accepted that 'simpler' organisms have less complex networks and that 'more advanced' organisms add new elements to the canonical 'cores' of these networks. However, analyses of recently sequenced genomes, including sea urchin, amphioxus, and sea anemone, challenge this notion [6-8]. For instance, we have shown that the evolution of the apoptotic regulatory network consists of a succession of lineage-specific expansions and losses, which, combined with the limited number of 'apoptotic' protein families, has resulted in apparent similarities between networks in different organisms that mask an underlying complex evolutionary history [9]. Here, we focus our analysis on the innate immune system and discuss the potential effects of domain rearrangements on network topology.

**Figure 1:**
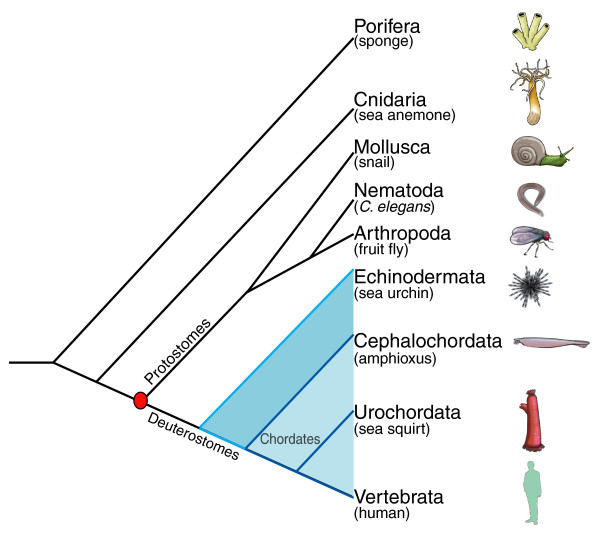
Evolutionary relationships of select metazoans. Taxa are arranged in descending order of phylogenetic emergence relative to vertebrates. The protostomes/deuterostomes split is indicated by a red circle. The blue shading is used to distinguish deuterostomes from all other animals. One branch of the deuterostomes includes the chordates (shown against a light blue background) and the other includes the echinoderms (shown against a deep blue background). Times of phylogenetic divergence are not to scale, and the tree branches are intended only to depict general relationships. The phylogenetic relationships between chordates described here are based on the current view that the cephalochordate is the most basal group in the extant chordate lineage [19-21].

The innate immune system mediates the primary line of defense against bacterial and viral infection and has distinctive roles in inflammatory diseases as well as in cancer [10-12]. In evolutionary terms, innate immunity is very ancient, and several of its mediators can be traced to the basal metazoans (that is, Porifera [13] and Cnidaria [14]). Defense systems that share similarity to animals' innate immunity have also been described in plants, although the exact relationships between these two systems are not clear [15,16]. The evolutionary history of innate immunity and its relationship to adaptive immune systems is of profound significance to our understanding of immune competence, interrelationships of immune mediators, and immune regulatory networks [17,18]. The recent sequencing of the amphioxus and sea urchin genomes, which occupy critical positions in the evolution of the deuterostomes (Figure 1), provides a basis for approaching this broad question.

Sea urchin, an echinoderm, is a representative of one of the two main branches of the deuterostome phylogeny [6]. Amphioxus, a cephalochordate, coming from one of the most basal groups in the extant chordate lineage [19-21], represents the other (Figure 1). A large expansion in several multigene families encoding pathogen recognition molecules relative to both vertebrates, such as mammals, and invertebrates, such as *C. elegans *and *D. melanogaster*, was reported in sea urchin [22,23]. Using different bioinformatics resources and tools as well as directed analysis of specific gene transcripts, we studied the innate immune genes in the recently completed amphioxus genome. We found a similar expansion in the numbers of innate receptors; however, unlike sea urchin, much of this expansion in amphioxus consists of genes with novel domain combinations. It is rather unexpected that such radical changes can occur in a relatively conserved network. At this point, amphioxus seems to be unique in the scale of its novel domain rearrangements, although the phenomenon of domain shuffling is likely to be a common mechanism of genome evolution. The extent of such changes in amphioxus highlights the importance of this mechanism in the evolutionary development of the innate immune system.

## Results

### Large multigene families encoding innate receptors

Innate immune responses depend on several families of pattern-recognition receptors that recognize pathogen-associated molecular patterns and cellular danger signals, which originate from invading pathogens or are released by dying or injured cells. Two families of pattern-recognition receptors, the transmembrane Toll-like receptors (TLRs) [24-26] and the intracellular NOD-like receptors (NLRs) [27-29], are of particular interest because of their role in a number of diseases. Major differences in the numbers of the above pattern-recognition receptors, as well as in other receptors, such as scavenger receptor cysteine-rich (SRCR) proteins [30], have been reported in sea urchin relative to both vertebrates and other invertebrates [22,23]. A similar expansion in these families is seen in the amphioxus genome (Table 1; Additional data file 3). The several-fold increases in the number of genes in these families in both sea urchin and amphioxus over other known invertebrates and vertebrates suggest that there is considerably more specificity in innate recognition in the former two species. It appears as if expansion of innate receptors is a shared characteristic of representatives of both arms of deuterostome evolution (Figure 1). From the standpoint of mammalian immunity, the findings in amphioxus are most interesting as the phenomena along the chordate arm of evolution has been lost in higher vertebrates; relatively few members of these families of innate receptors are found in vertebrate genomes.

**Table 1:** Expansion of protein families with innate immunity domains in amphioxus

Genome	TIR	NACHT
*Homo sapiens *(human)	24 (23)	23 (22)
*Mus musculus *(mouse)	24 (22)	33 (33)
*Canis familiaris *(dog)	26 (25)	17 (17)
*Gallus gallus *(chicken)	28 (27)	6 (6)
*Xenopus tropicalis *(western clawed frog)	28 (28)	22 (21)
*Danio rerio *(zebrafish)	30 (29)	21 (19)
*Fugu rubripes *(Japanese pufferfish)	17 (16)	180 (116)
*Tetraodon nigroviridis *(green pufferfish)	23 (20)	80 (11)
*Ciona intestinalis *(transparent sea squirt)	4 (4)	49 (45)
***Branchiostoma floridae *(amphioxus)**	**134 (125)**	**95 (94)**
*Strongylocentrotus purpuratus *(purple sea urchin)	244 (216)	326 (320)
*Drosophila melanogaster *(fruit fly)	11 (11)	0
*Caenorhabdidits elegans*	2 (2)	0
*Nematostella vectensis *(sea anemone)	7 (7)	45 (43)

The value in each domain category for each species is the total number of full-length protein sequence hits, with the number confirmed by Pfam Protein Search or NCBI CD-Search under the default threshold shown in parentheses. Because of the extreme diversity of both TIR and NACHT domains and experimental verification of only limited numbers of gene predictions, the numbers of predicted proteins in all recently sequenced genomes are considered as approximations, dependent on significance thresholds for gene predictions and specific homology recognition tools used in the analysis. For a detailed list of protein sequences, see Additional data files 1 and 2.

### The domain content of innate receptors in amphioxus is unique

**Figure 2:**
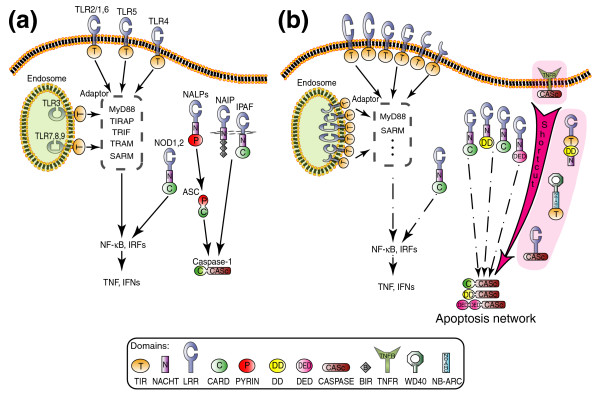
The diversification of the innate immune arsenal in amphioxus. **(a) **A simplified model of extracellular and intracellular innate immune signaling in human. TLR signaling involves recruitment of a number of TIR domain-containing adaptors, including myeloid differentiation factor 88 (MyD88), TIR domain-containing adaptor protein (TIRAP), TIR domain-containing adaptor inducing interferon-β (TRIF), TRIF-related adaptor molecule (TRAM), and sterile α and HEAT-Armadillo motifs containing protein (SARM), which in turn activates transcription factors such as nuclear factor-κB (NF-κB) and interferon regulatory factors (IRFs) that ultimately lead to tumor necrosis factor (TNF) and type I interferon (IFN) production. NLR signaling can also stimulate inflammatory responses via the NF-κB pathway. Also, NLRs can form the inflammasome with apoptosis-associated speck-like protein (ASC) and procaspase-1, leading to the generation of the active form of interleukin (IL)-1β and IL-18. **(b) **The diversity of the innate immune system in amphioxus. Novel domain architectures as well as significant expansion in receptor number are evident. Selected 'direct connection' gene models are shown against a pink background. The cellular localization of amphioxus TLR proteins is still unclear; some of them could be localized in endosome in a manner equivalent to that seen in mammals. Domains: BIR, baculovirus inhibitor of apoptosis repeat domain [1]; CARD, caspase recruitment domain [1]; CASPASE, caspase [1]; DD, death domain [1]; DED, death effector domain [1]; IPAF, ICE (IL-1β converting enzyme) protease activating factor; LRR, leucine-rich repeat [24]; NACHT, NAIP, CIITA, HET-E, and TP1 [28]; NALP, NACHT, LRR, and PYRIN-domain-containing protein; NB-ARC, nucleotide-binding adaptor shared by APAF-1, R proteins, and CED-4 [42]; PYRIN, amino-terminal domain of protein pyrin [1]; TIR, Toll/interleukin-1 receptor [3,26]; TNFR, tumor necrosis factor receptor [59]; WD40, Trp-Asp 40 [60].

**Figure 3:**
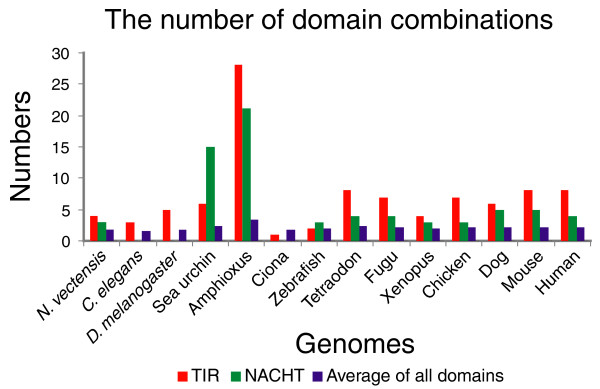
Different domain combinations in innate immunity receptor families. Numbers of different domains that combine with an individual TIR or NACHT domain in each designated genome are displayed. 'Average of all domains' (purple bars) means the average of domain combinations over all domains found in a genome. A detailed list of partner domains that combine with TIR or NACHT in each genome is given in Additional data file 4. The absolute numbers differ slightly when different Ensembl protein datasets or thresholds are used, but the relative fluctuations between different genomes are the same.

TLRs consist of multiple leucine-rich repeats (LRRs) at the amino terminus and a TIR domain at the carboxyl terminus that recruits TIR domain-containing adaptors for downstream signaling [2,31] (Figure 2a); examples (in human) are myeloid differentiation factor 88 (MyD88), TIR domain-containing adaptor protein (TIRAP), TIR domain-containing adaptor inducing interferon-β (TRIF), TRIF-related adaptor molecule (TRAM), and sterile α and HEAT-Armadillo motifs containing protein (SARM). Approximately eight domain combinations containing the TIR domain occur in mammals, five in *Drosophila*, and three in *C. elegans *(Figure 3; Additional data file 4). TIR domain combinations seen in *Drosophila *and *C. elegans *are also found in human. In contrast, 20 (out of a total of 28) domain combinations containing a TIR domain in amphioxus are specific to this organism. The difference with sea urchin is of particular note, since only about six TIR domain combinations exist in sea urchin, although the number of proteins containing TIR domains in sea urchin is even larger than in amphioxus (Table 1).

NLRs contain a nucleotide binding NACHT (domain present in neuronal apoptosis inhibitory protein (NAIP), CIITA, HET-E, and TP1) domain and are members of a distinct subfamily of the AAA+ (ATPase associated with diverse cellular activities) family [32]. In vertebrates, NLRs possess one of several types of linker domains (CARD, PYRIN/PAAD [amino-terminal domain of protein pyrin/pyrin, AIM (absent-in-melanoma), ASC (apoptosis-associated speck-like protein), and DD-like], or BIR (baculovirus inhibitor of apoptosis repeat)) at the amino terminus and multiple LRRs at the carboxyl terminus that effect pathogen recognition [3,28] (Figure 2a). Upon activation, NLRs are believed to assemble into complexes (inflammasomes) and recruit and activate additional proteins, such as caspase-1 and caspase-5 [33]. In amphioxus, approximately 21 different domain combinations involve NACHT domains, whereas approximately 5 are predicted in mammals (Figure 3; Additional data file 4). The NACHT domain is absent in *Drosophila *and *C. elegans*. Finally, it is noteworthy that in amphioxus SRCR-containing proteins, the SRCR domain - another domain related to the innate immune system [30] - is also combined with a greater diversity of other domains than in comparable proteins of sea urchins and other animals (Additional data file 3), similar to observations noted about TIR and NACHT domains.

### Unique domain combinations imply unique topology of innate receptors

Activation of downstream host-defense mechanisms occurs via specialized signal transduction pathways that are mediated by a number of specific protein domains [3,34]. Domain shuffling can create multidomain proteins with new domain architectures and functions, including proteins serving as novel connectors in regulatory pathways [5]. Organisms differ not only in the sizes of protein families, but also in their domain architectures - the combination of different domains in multidomain proteins. To study such differences, we have previously developed the Comparative Analysis of Protein Domain Organization (CADO) software package [35], which provides a tool that can visualize and analyze domain combinations of proteins in a given genome. CADO defines protein organization as a graph in which protein domains are represented as nodes, and domain combinations, defined as instances of two domains found in one protein, are represented as edges (lines). Using CADO, domain graphs of two (or more) genomes can be compared, identifying similarities and differences both in individual domain combinations and in general topology of the domain graph [35,36].

**Figure 4:**
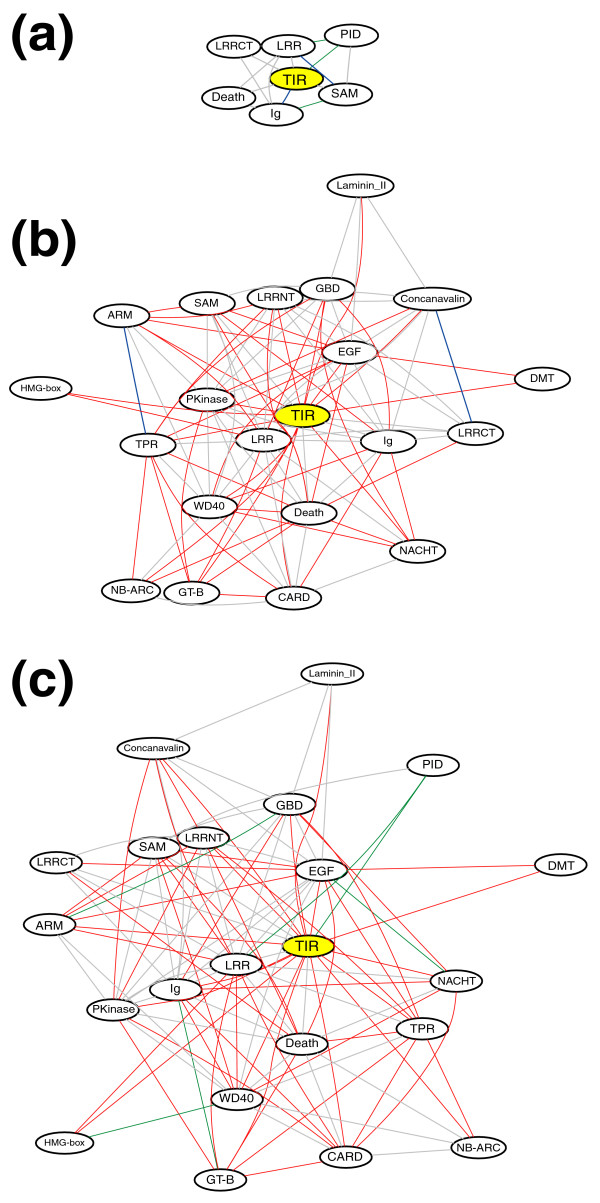
Difference between protein domain networks involving the TIR domain in amphioxus, human, and sea urchin. **(a) **A comparison by CADO of the domain network anchored by the TIR domain in human and sea urchin. **(b) **CADO picture anchored by the TIR domain between human and amphioxus. **(c) **CADO picture anchored by the TIR domain between amphioxus and sea urchin. A line connecting two domains indicates a predicted single protein domain combination. Common domain combinations between the selected genomes are shown in gray; amphioxus-specific combinations are shown in red; human-specific combinations are shown in blue; and sea urchin-specific combinations are shown in green. Please note that to simplify the graphical representation, Pfam clans are adopted for some Pfam domains. The CADO picture may differ slightly when different thresholds are used, for instance, the Ig-TIR domain combination can be found in sea urchin when using SMART domain definitions.

CADO-based analysis was applied in order to determine if the expansion of the innate immunity receptor families also resulted in changes to the overall topology of the innate immune network in terms of unique domain combinations. Based on the comparison of amphioxus, human, and sea urchin genomes, the TIR domain combination repertoire of sea urchin is very close to that seen in human (Figure 4a), although the copy number of TIR-containing sequences between human and sea urchin differs approximately 10-fold (Table 1). Almost all the TIR domain combinations present in human and sea urchin can also be identified in amphioxus, which are shown by gray lines in Figure 4b,c; however, amphioxus has many more unique TIR domain combinations. Most of the domain combinations seen in amphioxus are specific to this organism (red lines in Figure 4b,c).

Similar observations have been made for NLRs. In this case, most of the differences reside in the amino-terminal domain. Instead of a vertebrate-specific PYRIN/PAAD domain, amphioxus can have CARD, DD, or DED as connector domains (Figure 2b). The DD-NACHT and DED-NACHT direct domain combinations seen in NLRs have not been seen in vertebrates but are found in sea urchin [6,23] and *Nematostella vectensis *[7,37]. Because the amino-terminal prodomain in amphioxus caspases can be any of the DD, DED, or CARD types, these hybrid intracellular pathogen recognition receptors may directly trigger the apoptosis response (Figure 2b), rather than function through an ASC-like 'hub'.

Other types of hybrid genes, including those encoding tumor necrosis factor receptor (TNFR)-caspase, LRRs-caspase, TIR-NACHT, TIR-[NB-ARC]-WD40s (NB-ARC is nucleotide-binding adaptor shared by APAF-1, R proteins, and CED-4; WD40 is Trp-Asp 40), TIR-sterile alpha motif (SAM), TIR-Laminin and so on, which potentially could mediate immune-related functions, have also been identified in the amphioxus genome.

### The unique predicted hybrid genes are expressed

Despite the presence of unusually complex patterns of repetitive DNA, the current assembly of the amphioxus genome is generally highly reliable [19]; notwithstanding this high level of confidence in the hybrid gene predictions, it is essential to note that cDNA transcripts of many of the predicted hybrid proteins have been recovered. The TNFR-caspase domain protein (Joint Genome Institute (JGI) model: Brafl1_82667) represents one of the shortcut pathways of particular interest (Figure 2b; Additional data file 6 part a). This predicted transmembrane protein contains an extracellular TNFR domain and an intracellular caspase domain and presumably provides a shortcut between inflammatory-type signals and cell death. cDNA analyses not only validate this domain architecture but also have identified other related gene sequences, including more than one type of both TNFR and caspase domains. These transcripts are the products of three genetic regions on scaffolds: _41, _114, and _457. Other examples include cDNAs encoding: the death-caspase domain combination predicted in model Brafl1_105741 (fgenesh2_pg.scaffold_505000014); the death-NACHT domain combination in model Brafl1_82459 (fgenesh2_pg.scaffold_111000114) and Brafl1_89453 (fgenesh2_pg.scaffold_187000018); the DED-NACHT combination in model Brafl1_98233 (fgenesh2_pg.scaffold_317000043); and the TIR-SAM combination in model Brafl1_131196 (estExt_fgenesh2_pg.C_5050026), which are described in Additional data file 5.

The recovery of transcripts corresponding to the 'direct connector' genes is, in itself, important as many of these genes most likely exhibit developmental stage-specific expression, may be expressed in relatively low abundance, and/or are transcribed in cells that are present in relatively low numbers or are undergoing apoptosis. Efforts to locate the expression of hybrid genes are currently underway.

## Discussion

The large-scale expansion of several families of innate receptors in amphioxus parallels that seen in sea urchin and is a shared feature of both sides of the deuterostome split. The phenomenon of lineage-specific gene expansion has also been reported for protein families in other genomes [38]. Further sequencing efforts are required to establish if the large numbers of novel domain architectures in innate immune-related genes are specific only to amphioxus, are specific only to deuterostomes, or represent a more general mechanism. We stress that the exact functions of these genes from amphioxus remain unknown and that further experimental work is needed; however, it is reasonable to hypothesize that the wide variety of domain combinations reported here likely expands the functions of the innate immune system in amphioxus. It is tempting to speculate that perhaps functionality of the amphioxus specific genes is provided by other regulatory mechanisms in vertebrates and that better understanding of the functions of novel amphioxus genes may help in discovering these mechanisms.

Many of the domain combinations in amphioxus are present in separate proteins in vertebrates that are interconnected by multistep signaling pathways (examples shown in Figure 2b and Additional data file 6). As such, the amphioxus proteins can be viewed as shortcuts between two endpoints. The presence of such shortcuts would change the topology of the network in a way that can be described as a difference between 'hub-and-spoke' versus 'direct connection' networks [39]. For instance, a TIR-NACHT architecture, present in amphioxus but absent in vertebrates, is a shortcut that directly connects the extra- and intracellular pathogenic pattern-recognition pathways (Figure 2b). In human, these two pathways are likely connected 'indirectly' by transforming growth factor-β activated kinase 1 (TAK1), receptor-interacting protein 2 (RIP2), and/or other molecules, although the detailed relationships of this functional integration are not resolved [3,34,40]. Proteins composed of LRRs or TNFR domains that directly connect to the caspase domain could provide direct links between pathogen recognition and apoptosis (Figure 2b; Additional data file 6). All these proteins contain the conserved QACXG (where X is R, Q, or G) pentapeptide active-site motif [41] in their caspase domains and, thus, likely have proteolytic function (Additional data file 7). Amphioxus proteins that combine a TIR domain with an NB-ARC domain [42] and WD40 repeats share features with Apaf-1 (apoptotic protease activating factor 1; a central regulator of apoptosis in animals, which consists of a CARD domain, an NB-ARC domain, and multiple WD40 repeats). The association of these structures with an amino-terminal TIR domain suggests a direct link between the innate immunity and apoptosis networks.

In general, the innate immunity and apoptosis networks, which interact through a complex system of signaling pathways in human and other vertebrates, are closely intertwined in amphioxus through multiple direct connection proteins. It is possible that the close relationship between these two major systems represents an important innovation at the base of the deuterostome lineage that has been preserved throughout the vertebrates, albeit implemented through different mechanisms. It has been shown that the artificial joining of domains in novel combinations [43-45] create new signaling pathways. Specifically, the chimeric adaptor proteins, which contain a DED with a phosphotyrosine-binding (PTB) or Src homology 2 (SH2) domain, can redirect tyrosine kinase signaling from survival and cell growth to apoptosis [45]. In another example, it has been shown that caspase can be activated by the chemically inducible dimerization (CID) signal, resulting in apoptosis when its catalytic domain is artificially fused to CID-binding domains [43]. These directed studies lend considerable support for potential functions of the multiple shortcut proteins that have been identified in amphioxus. Furthermore, the results suggest that engineering of constructs corresponding to the amphioxus chimeric molecules represents a viable approach for gaining a better understanding of how these molecules function in innate immunity. The presence of direct connectors has important consequences for the flexibility of the network. In the hub-and-spoke model, the number of possible connections is exponential, even with the linear growth of the number of proteins. A very large number of different 'direct connections' would be required to provide equivalent flexibility.

Although not characterized at the transcription level, some of the 'hub' domains and connections that are present in human can also be found in the cnidarian *N. vectensis *[14,46], such as the NACHT domain, the death-TIR connection, the Ig-TIR connection, and so on. Thus, the 'hub-and-spoke' model could be considered ancestral and was reduced in the arthropod and nematode lineage by eliminating some 'destinations' and/or even 'hubs' (for example, *C. elegans *has only one Toll-like receptor, TOL-l [47], and one SARM-like TIR domain containing adaptor, TIR-1 [48]; the NACHT domain is absent in both *C. elegans *and *Drosophila *(Table 1)). Taken together with the observations reported here, expansion appears to have occurred at the base of deuterostomes, and further evolution may well have proceeded independently in the echinoderm and cephalochordate branches. Although proteins with novel domain combinations also have been found in sea urchin [23,49], the extent of such direct connections appears to be far greater in amphioxus. It is reasonable to assume that some direct connections could have been lost with the emergence of the vertebrate adaptive immune system or effectively replaced by additional 'hub' molecules, such as the ASC in the vertebrate lineage [33]. In light of these changes, the topology of the network would become closer to that of the common ancestor. The coexistence of both shortcut and conventional pathways in an extant species is exceptional and underscores the potential relevance of amphioxus for understanding the selective advantages of such arrangements.

## Conclusion

Two aspects of genome architecture and complexity influence innate immunity in amphioxus. First, large-scale gene expansion, a characteristic shared with sea urchin, creates a greater level of potential specificity in several families of innate immune receptors than is found in species with adaptive immune systems and could result in refinement of immune function. Second, novel domain architectures and, in particular, direct connections (shortcuts) in regulatory pathways can introduce a more refined level of functional integration of networks than would likely be achieved by the simple duplication and subsequent divergence of genes encoding immune receptors. A model for expansion and the possibility of topology change of a network is presumed in the analyses of the amphioxus genome presented here. A corollary issue raised by these observations is whether specific features of the amphioxus genome, such as the extraordinary level of site variation and unusually complex patterns of repetitive DNA, factor in such changes. Irrespective of their origins, genes with novel architectures in amphioxus could potentially serve as a pathway-level 'Rosetta stone' for elucidating new regulatory connections in the innate systems of contemporary vertebrates, similar to approaches that are used to elucidate protein and regulatory complexes in prokaryotic genomes [50]. Assuming that such shortcuts impart selective advantage, there is reason to look for signaling alternatives that may emulate the predicted distinct function implicit in these unique hybrid structures.

## Materials and methods

### Datasets

The v.1.0 genome assembly and related gene models of amphioxus (*Branchiostoma floridae*) were obtained from the JGI [51] as were the genome assembly 1.0 and related protein set of the sea anemone (*N. vectensis*). The genome assembly Spur_v2.0 and the GLEAN3 gene models for the sea urchin (*Strongylocentrotus purpuratus*) were obtained from the Baylor College of Medicine Human Genome Sequencing Center [52]. The other genome sequences and corresponding protein sets, including human, mouse, dog, chicken, *Xenopus*, zebrafish, fugu, tetraodon, ciona, nematode (*C. elegans*), and fruit fly (*D. melanogaster*) were downloaded from Ensembl [53].

### Database search and sequence analysis

Several rounds of PSITBLASTN [54] searches were performed against each genome using known human TIR or NACHT domain amino acid sequences as seeds. Hits were mapped to the corresponding genome protein set in order to obtain the full-length protein sequences (for sea urchin and sea anemone, some of the gene models were in addition predicted by GenScan [55]). All identified genes were checked using: first, reciprocal BLAST analysis; second, Pfam protein searches, performed either locally or at the Pfam website [56], which also address the issue of family specificity, such as distinguishing NACHT domain from NB-ARC domain based on different hidden Markov models; third, NCBI CD-Search [57] and local RPS-BLAST search; and fourth, multiple sequence-alignment and phylogeny analysis.

### Domain combination analysis

Different combinations of innate immune domains identified in the aforementioned genomes were compared using the CADO [35] approach.

### RT-PCR confirmation of select modular transcripts

JGI-predicted models were used to develop PCR strategies for identifying cDNA transcripts. The predicted transcripts were placed onto the current assembly (v.1.0) using local BLAST (v.2.2.11) to verify genomic organization (for example, exon/intron structure and gene copy number). Primers were designed (from visual alignments or with Primer3 [58]) to span domain combinations and specific exon/intron boundaries. Primer design accommodated variations due to genetic polymorphism and haplotype complexity, a significant confounding aspect of this type of analysis. Total RNA was isolated from 30 animals using RNA-Bee (Tel-Test, Inc., Friendswood, TX, USA), and cDNA synthesis was primed using either poly-A or random hexamer strategies (SuperScriptIII, Invitrogen, Carlsbad, CA, USA). cDNAs were combined and served as templates for PCR amplification. Certain transcripts could be detected only after two rounds of nested PCR. Transcribed sequences with the expected length were sequenced to confirm the predicted gene models. The verified amphioxus gene models in this study have been deposited in the GenBank database under accession numbers [GenBank:EU049583] to [GenBank:EU049596] and [GenBank:EU279424] to [GenBank:EU279425] (Additional data file 5).

## Abbreviations

ASC, apoptosis-associated speck-like protein; CADO, Comparative Analysis of Protein Domain Organization; CARD, caspase recruitment domain; CID, chemically inducible dimerization; DD, death domain; DED, death effector domain; JGI, Joint Genome Institute; LRR, leucine-rich repeat; NACHT, domain present in NAIP, CIITA, HET-E, and TP1; NAIP, neuronal apoptosis inhibitory protein; NB-ARC, nucleotide-binding adaptor shared by APAF-1, R proteins, and CED-4; NLR, NOD-like receptor; PAAD, pyrin, AIM (absent-in-melanoma), ASC, and DD-like; PYRIN, amino-terminal domain of protein pyrin; SAM, sterile alpha motif; SARM, sterile α and HEAT-Armadillo motifs containing protein; SRCR, scavenger receptor cysteine-rich; TIR, Toll/interleukin-1 receptor; TLR, Toll-like receptor; TNFR, tumor necrosis factor receptor; WD40, Trp-Asp 40.

## Authors' contributions

QZ performed the sequence and domain analyses and prepared the figures. CMZ performed phylogenetic analyses. LJD developed approaches for identifying hybrid transcripts. MGM cloned and sequenced hybrid transcripts. YY contributed to the domain analyses of the predicted proteins. GWL interpreted immunology concepts. AG formulated the problem and planned the work. All authors contributed to the interpretation of the results and to writing of the paper.

## Additional data files

The following additional data files are available. Additional data file 1 is a table listing the TIR domain containing sequences in different genomes. Additional data file 2 is a table listing the NACHT domain containing sequences in different genomes. Additional data file 3 is a table listing the SRCR domain combinations in different genomes. Additional data file 4 is a table listing partner domains that combine with individual TIR or NACHT domains in different genomes. Additional data file 5 is a table listing the selected JGI-predicted amphioxus gene models that have been verified by RT-PCR. Additional data file 6 is a figure showing examples of novel domain combinations in amphioxus that represent the shortcuts between two or more proteins present in human. Additional data file 7 is a figure showing alignment of sequences in the vicinity of the catalytic center of the caspase domain from human caspases and amphioxus proteins with TNFR-caspase or LRRs-caspase architectures.

## Supplementary Material

Additional data file 1 TIR domain containing sequences in different genomes. Click here for file

Additional data file 2 NACHT domain containing sequences in different genomes. Click here for file

Additional data file 3 SRCR domain combinations in different genomes. Click here for file

Additional data file 4 Partner domains that combine with individual TIR or NACHT domains in different genomes. Click here for file

Additional data file 5 Selected JGI-predicted amphioxus gene models that have been verified by RT-PCR. Click here for file

Additional data file 6 Examples of novel domain combinations in amphioxus that represent the shortcuts between two or more proteins present in human. Click here for file

Additional data file 7 Alignment of sequences in the vicinity of the catalytic center of the caspase domain from human caspases and amphioxus proteins with TNFR-caspase or LRRs-caspase architectures. Click here for file
