# Supplementary material for: Novel genes dramatically alter regulatory network topology in amphioxus
Source: Genome Biol. 2008 Aug 4;9(8):R123. doi: 10.1186/gb-2008-9-8-r123 (PMC2575513; doi:10.1186/gb-2008-9-8-r123)
Supplement: Additional data file 1 — TIR domain containing sequences in different genomes. [file gb-2008-9-8-r123-S1.pdf]

Additional Table 1. List of TIR domain containing sequences in different genomes

Genome	Data Source	Protein Identifier
H. sapiens (human)	Ensembl release 38 'pep.abinitio'	GENSCAN00000006442
	Ensembl release 38 'pep.abinitio'	GENSCAN00000006563
	Ensembl release 38 'pep.abinitio'	GENSCAN00000006564
	Ensembl release 38 'pep.abinitio'	GENSCAN00000007356
	Ensembl release 38 'pep.abinitio'	GENSCAN00000014110
	Ensembl release 38 'pep.abinitio'	GENSCAN00000016770
	Ensembl release 38 'pep.abinitio'	GENSCAN00000023836
	Ensembl release 38 'pep.abinitio'	GENSCAN00000026205
	Ensembl release 38 'pep.abinitio'	GENSCAN00000026337
	Ensembl release 38 'pep.abinitio'	GENSCAN00000026341
	Ensembl release 38 'pep.abinitio'	GENSCAN00000032006
	Ensembl release 38 'pep.abinitio'	GENSCAN00000032010
	Ensembl release 38 'pep.abinitio'	GENSCAN00000034144
	Ensembl release 38 'pep.abinitio'	GENSCAN00000034623
	Ensembl release 38 'pep.abinitio'	GENSCAN00000040296
	Ensembl release 38 'pep.abinitio'	GENSCAN00000046730
	Ensembl release 38 'pep.abinitio'	GENSCAN00000051721
	Ensembl release 38 'pep.abinitio'	GENSCAN00000052592
	Ensembl release 38 'pep.abinitio'	GENSCAN00000057266
	Ensembl release 38 'pep.abinitio'	GENSCAN00000058482
	Ensembl release 38 'pep.abinitio'	GENSCAN00000066012
	Ensembl release 38 'pep.abinitio'	GENSCAN00000050507
	Ensembl release 38 'pep.abinitio'	GENSCAN00000052380
H. sapiens (human)	Ensembl release 38 'pep'	ENSP00000003834
	Ensembl release 38 'pep'	ENSP00000072516
	Ensembl release 38 'pep'	ENSP00000233946
	Ensembl release 38 'pep'	ENSP00000233954
	Ensembl release 38 'pep'	ENSP00000233957
	Ensembl release 38 'pep'	ENSP00000260010
	Ensembl release 38 'pep'	ENSP00000264257
	Ensembl release 38 'pep'	ENSP00000264260
	Ensembl release 38 'pep'	ENSP00000279992
	Ensembl release 38 'pep'	ENSP00000296795
	Ensembl release 38 'pep'	ENSP00000305200
	Ensembl release 38 'pep'	ENSP00000308925
	Ensembl release 38 'pep'	ENSP00000309253
	Ensembl release 38 'pep'	ENSP00000311174
	Ensembl release 38 'pep'	ENSP00000323890
	Ensembl release 38 'pep'	ENSP00000339832

	Ensembl release 38 'pep'	ENSP00000340089
	Ensembl release 38 'pep'	ENSP00000344347
	Ensembl release 38 'pep'	ENSP00000344976
	Ensembl release 38 'pep'	ENSP00000348619
	Ensembl release 38 'pep'	ENSP00000349736
	Ensembl release 38 'pep'	ENSP00000354932
	Ensembl release 38 'pep'	ENSP00000370028
	Ensembl release 38 'pep'	ENSP00000371960
H. sapiens (human)	Swiss-Prot	P14778|IL1R1_HUMAN
	Swiss-Prot	Q9NPH3|IL1AP_HUMAN
	Swiss-Prot	Q13478|IL18R_HUMAN
	Swiss-Prot	O95256|I18RA_HUMAN
	Swiss-Prot	Q01638|ILRL1_HUMAN
	Swiss-Prot	Q9HB29|ILRL2_HUMAN
	Swiss-Prot	Q9NZN1|IRPL1_HUMAN
	Swiss-Prot	Q9NP60|IRPL2_HUMAN
	Swiss-Prot	Q6IA17|SIGIR_HUMAN
	Swiss-Prot	Q15399|TLR1_HUMAN
	Swiss-Prot	O60603|TLR2_HUMAN
	Swiss-Prot	O15455|TLR3_HUMAN
	Swiss-Prot	O00206|TLR4_HUMAN
	Swiss-Prot	O60602|TLR5_HUMAN
	Swiss-Prot	Q9Y2C9|TLR6_HUMAN
	Swiss-Prot	Q9NYK1|TLR7_HUMAN
	Swiss-Prot	Q9NR97|TLR8_HUMAN
	Swiss-Prot	Q9NR96|TLR9_HUMAN
	Swiss-Prot	Q9BXR5|TLR10_HUMAN
	Swiss-Prot	Q99836|MYD88_HUMAN
	TrEMBL	Q8IUC6|Q8IUC6_HUMAN
	TrEMBL	Q86XR7|Q86XR7_HUMAN
	Swiss-Prot	P58753|TIRAP_HUMAN
	Swiss-Prot	Q6SZW1|SARM1_HUMAN
M. musculus (mouse)	Ensembl release 38 'pep.abinitio'	GENSCAN00000153036
	Ensembl release 38 'pep.abinitio'	GENSCAN00000155466
	Ensembl release 38 'pep.abinitio'	GENSCAN00000157254
	Ensembl release 38 'pep.abinitio'	GENSCAN00000159308
	Ensembl release 38 'pep.abinitio'	GENSCAN00000161585
	Ensembl release 38 'pep.abinitio'	GENSCAN00000162981
	Ensembl release 38 'pep.abinitio'	GENSCAN00000173273
	Ensembl release 38 'pep.abinitio'	GENSCAN00000173275
	Ensembl release 38 'pep.abinitio'	GENSCAN00000175431
	Ensembl release 38 'pep.abinitio'	GENSCAN00000175918
	Ensembl release 38 'pep.abinitio'	GENSCAN00000184846

	Ensembl release 38 'pep.abinitio'	GENSCAN00000187775
	Ensembl release 38 'pep.abinitio'	GENSCAN00000197386
	Ensembl release 38 'pep.abinitio'	GENSCAN00000209084
	Ensembl release 38 'pep.abinitio'	GENSCAN00000214191
	Ensembl release 38 'pep.abinitio'	GENSCAN00000214924
	Ensembl release 38 'pep.abinitio'	GENSCAN00000217755
	Ensembl release 38 'pep.abinitio'	GENSCAN00000218141
	Ensembl release 38 'pep.abinitio'	GENSCAN00000218146
	Ensembl release 38 'pep.abinitio'	GENSCAN00000218583
	Ensembl release 38 'pep.abinitio'	GENSCAN00000217148
	Ensembl release 38 'pep.abinitio'	GENSCAN00000221196
M. musculus (mouse)	Ensembl release 38 'pep'	ENSMUSP00000023156
	Ensembl release 38 'pep'	ENSMUSP00000027237
	Ensembl release 38 'pep'	ENSMUSP00000029623
	Ensembl release 38 'pep'	ENSMUSP00000034056
	Ensembl release 38 'pep'	ENSMUSP00000034540
	Ensembl release 38 'pep'	ENSMUSP00000035092
	Ensembl release 38 'pep'	ENSMUSP00000036762
	Ensembl release 38 'pep'	ENSMUSP00000043101
	Ensembl release 38 'pep'	ENSMUSP00000045770
	Ensembl release 38 'pep'	ENSMUSP00000051059
	Ensembl release 38 'pep'	ENSMUSP00000054914
	Ensembl release 38 'pep'	ENSMUSP00000057529
	Ensembl release 38 'pep'	ENSMUSP00000060793
	Ensembl release 38 'pep'	ENSMUSP00000061853
	Ensembl release 38 'pep'	ENSMUSP00000062096
	Ensembl release 38 'pep'	ENSMUSP00000066239
	Ensembl release 38 'pep'	ENSMUSP00000068906
	Ensembl release 38 'pep'	ENSMUSP00000074381
	Ensembl release 38 'pep'	ENSMUSP00000074917
	Ensembl release 38 'pep'	ENSMUSP00000077915
	Ensembl release 38 'pep'	ENSMUSP00000082207
	Ensembl release 38 'pep'	ENSMUSP00000085298
	Ensembl release 38 'pep'	ENSMUSP00000085305
	Ensembl release 38 'pep'	ENSMUSP00000092629
C. familiaris (dog)	Ensembl release 38 'pep.abinitio'	GENSCAN00000000432
	Ensembl release 38 'pep.abinitio'	GENSCAN00000003693
	Ensembl release 38 'pep.abinitio'	GENSCAN00000012336
	Ensembl release 38 'pep.abinitio'	GENSCAN00000012678
	Ensembl release 38 'pep.abinitio'	GENSCAN00000012892
	Ensembl release 38 'pep.abinitio'	GENSCAN00000018400
	Ensembl release 38 'pep.abinitio'	GENSCAN00000019922

	Ensembl release 38 'pep.abinitio'	GENSCAN00000020531
	Ensembl release 38 'pep.abinitio'	GENSCAN00000020532
	Ensembl release 38 'pep.abinitio'	GENSCAN00000020533
	Ensembl release 38 'pep.abinitio'	GENSCAN00000026555
	Ensembl release 38 'pep.abinitio'	GENSCAN00000026999
	Ensembl release 38 'pep.abinitio'	GENSCAN00000037852
	Ensembl release 38 'pep.abinitio'	GENSCAN00000043592
	Ensembl release 38 'pep.abinitio'	GENSCAN00000046789
	Ensembl release 38 'pep.abinitio'	GENSCAN00000051228
	Ensembl release 38 'pep.abinitio'	GENSCAN00000052684
	Ensembl release 38 'pep.abinitio'	GENSCAN00000052686
	Ensembl release 38 'pep.abinitio'	GENSCAN00000056053
	Ensembl release 38 'pep.abinitio'	GENSCAN00000058584
	Ensembl release 38 'pep.abinitio'	GENSCAN00000072074
	Ensembl release 38 'pep.abinitio'	GENSCAN00000072075
	Ensembl release 38 'pep.abinitio'	GENSCAN00000073119
	Ensembl release 38 'pep.abinitio'	GENSCAN00000038012
	Ensembl release 38 'pep.abinitio'	GENSCAN00000060453
C. familiaris (dog)	Ensembl release 38 'pep'	ENSCAFP00000000299
	Ensembl release 38 'pep'	ENSCAFP00000003121
	Ensembl release 38 'pep'	ENSCAFP00000003126
	Ensembl release 38 'pep'	ENSCAFP00000003131
	Ensembl release 38 'pep'	ENSCAFP00000003133
	Ensembl release 38 'pep'	ENSCAFP00000003138
	Ensembl release 38 'pep'	ENSCAFP00000007387
	Ensembl release 38 'pep'	ENSCAFP00000009741
	Ensembl release 38 'pep'	ENSCAFP00000011004
	Ensembl release 38 'pep'	ENSCAFP00000012269
	Ensembl release 38 'pep'	ENSCAFP00000015327
	Ensembl release 38 'pep'	ENSCAFP00000016726
	Ensembl release 38 'pep'	ENSCAFP00000017193
	Ensembl release 38 'pep'	ENSCAFP00000020084
	Ensembl release 38 'pep'	ENSCAFP00000020679
	Ensembl release 38 'pep'	ENSCAFP00000023836
	Ensembl release 38 'pep'	ENSCAFP00000023840
	Ensembl release 38 'pep'	ENSCAFP00000026353
	Ensembl release 38 'pep'	ENSCAFP00000027553
G. gallus (chicken)	Ensembl release 38 'pep.abinitio'	GENSCAN00000000677
	Ensembl release 38 'pep.abinitio'	GENSCAN00000006545
	Ensembl release 38 'pep.abinitio'	GENSCAN00000006765
	Ensembl release 38 'pep.abinitio'	GENSCAN00000008344
	Ensembl release 38 'pep.abinitio'	GENSCAN00000010251

	Ensembl release 38 'pep.abinitio'	GENSCAN00000010939
	Ensembl release 38 'pep.abinitio'	GENSCAN00000019500
	Ensembl release 38 'pep.abinitio'	GENSCAN00000020129
	Ensembl release 38 'pep.abinitio'	GENSCAN00000021514
	Ensembl release 38 'pep.abinitio'	GENSCAN00000021594
	Ensembl release 38 'pep.abinitio'	GENSCAN00000023012
	Ensembl release 38 'pep.abinitio'	GENSCAN00000023251
	Ensembl release 38 'pep.abinitio'	GENSCAN00000023581
	Ensembl release 38 'pep.abinitio'	GENSCAN00000024545
	Ensembl release 38 'pep.abinitio'	GENSCAN00000025123
	Ensembl release 38 'pep.abinitio'	GENSCAN00000029580
	Ensembl release 38 'pep.abinitio'	GENSCAN00000034556
	Ensembl release 38 'pep.abinitio'	GENSCAN00000044254
	Ensembl release 38 'pep.abinitio'	GENSCAN00000046216
	Ensembl release 38 'pep.abinitio'	GENSCAN00000050319
	Ensembl release 38 'pep.abinitio'	GENSCAN00000054052
	Ensembl release 38 'pep.abinitio'	GENSCAN00000057832
	Ensembl release 38 'pep.abinitio'	GENSCAN00000057834
	Ensembl release 38 'pep.abinitio'	GENSCAN00000065494
	Ensembl release 38 'pep.abinitio'	GENSCAN00000070507
	Ensembl release 38 'pep.abinitio'	GENSCAN00000018661
	Ensembl release 38 'pep.abinitio'	GENSCAN00000075100
G. gallus (chicken)	Ensembl release 38 'pep'	ENSGALP00000001118
	Ensembl release 38 'pep'	ENSGALP00000001610
	Ensembl release 38 'pep'	ENSGALP00000005677
	Ensembl release 38 'pep'	ENSGALP00000006773
	Ensembl release 38 'pep'	ENSGALP00000009551
	Ensembl release 38 'pep'	ENSGALP00000011727
	Ensembl release 38 'pep'	ENSGALP00000013260
	Ensembl release 38 'pep'	ENSGALP00000014431
	Ensembl release 38 'pep'	ENSGALP00000015020
	Ensembl release 38 'pep'	ENSGALP00000015286
	Ensembl release 38 'pep'	ENSGALP00000021914
	Ensembl release 38 'pep'	ENSGALP00000026211
	Ensembl release 38 'pep'	ENSGALP00000026727
	Ensembl release 38 'pep'	ENSGALP00000027049
	Ensembl release 38 'pep'	ENSGALP00000027050
	Ensembl release 38 'pep'	ENSGALP00000027053
	Ensembl release 38 'pep'	ENSGALP00000027055
	Ensembl release 38 'pep'	ENSGALP00000028126
	Ensembl release 38 'pep'	ENSGALP00000029597
	Ensembl release 38 'pep'	ENSGALP00000033864

X. tropicalis (western clawed frog)	Ensembl release 38 'pep.abinitio'	FGENESH00000100541
	Ensembl release 38 'pep.abinitio'	FGENESH00000109261
	Ensembl release 38 'pep.abinitio'	FGENESH00000109955
	Ensembl release 38 'pep.abinitio'	FGENESH00000117286
	Ensembl release 38 'pep.abinitio'	GENSCAN00000008389
	Ensembl release 38 'pep.abinitio'	GENSCAN00000012275
	Ensembl release 38 'pep.abinitio'	GENSCAN00000014909
	Ensembl release 38 'pep.abinitio'	GENSCAN00000016099
	Ensembl release 38 'pep.abinitio'	GENSCAN00000016118
	Ensembl release 38 'pep.abinitio'	GENSCAN00000031703
	Ensembl release 38 'pep.abinitio'	GENSCAN00000031705
	Ensembl release 38 'pep.abinitio'	GENSCAN00000031706
	Ensembl release 38 'pep.abinitio'	GENSCAN00000034368
	Ensembl release 38 'pep.abinitio'	GENSCAN00000035339
	Ensembl release 38 'pep.abinitio'	GENSCAN00000038838
	Ensembl release 38 'pep.abinitio'	GENSCAN00000043392
	Ensembl release 38 'pep.abinitio'	GENSCAN00000046153
	Ensembl release 38 'pep.abinitio'	GENSCAN00000046203
	Ensembl release 38 'pep.abinitio'	GENSCAN00000048308
	Ensembl release 38 'pep.abinitio'	GENSCAN00000054063
	Ensembl release 38 'pep.abinitio'	GENSCAN00000060523
	Ensembl release 38 'pep.abinitio'	GENSCAN00000062691
	Ensembl release 38 'pep.abinitio'	GENSCAN00000062737
	Ensembl release 38 'pep.abinitio'	GENSCAN00000065633
	Ensembl release 38 'pep.abinitio'	GENSCAN00000068925
	Ensembl release 38 'pep.abinitio'	GENSCAN00000071750
	Ensembl release 38 'pep.abinitio'	GENSCAN00000071764
	Ensembl release 38 'pep.abinitio'	GENSCAN00000008482
X. tropicalis (western clawed frog)	Ensembl release 38 'pep'	ENSXETP00000003822
	Ensembl release 38 'pep'	ENSXETP00000009979
	Ensembl release 38 'pep'	ENSXETP00000011729
	Ensembl release 38 'pep'	ENSXETP00000012948
	Ensembl release 38 'pep'	ENSXETP00000012956
	Ensembl release 38 'pep'	ENSXETP00000032872
	Ensembl release 38 'pep'	ENSXETP00000032921
	Ensembl release 38 'pep'	ENSXETP00000033579
	Ensembl release 38 'pep'	ENSXETP00000038393
	Ensembl release 38 'pep'	ENSXETP00000038824
	Ensembl release 38 'pep'	ENSXETP00000039929
	Ensembl release 38 'pep'	ENSXETP00000047459
	Ensembl release 38 'pep'	ENSXETP00000047482
	Ensembl release 38 'pep'	ENSXETP00000055330

	Ensembl release 38 'pep'	ENSXETP00000056436
	Ensembl release 38 'pep'	ENSXETP00000056762
	Ensembl release 38 'pep'	ENSXETP00000056818
	Ensembl release 38 'pep'	ENSXETP00000057554
	Ensembl release 38 'pep'	ENSXETP00000057560
D. rerio (zebrafish)	Ensembl release 38 'pep.abinitio'	FGENESH00000064811
	Ensembl release 38 'pep.abinitio'	FGENESH00000087033
	Ensembl release 38 'pep.abinitio'	GENSCAN00000001002
	Ensembl release 38 'pep.abinitio'	GENSCAN00000004216
	Ensembl release 38 'pep.abinitio'	GENSCAN00000004226
	Ensembl release 38 'pep.abinitio'	GENSCAN00000009227
	Ensembl release 38 'pep.abinitio'	GENSCAN00000009229
	Ensembl release 38 'pep.abinitio'	GENSCAN00000009235
	Ensembl release 38 'pep.abinitio'	GENSCAN00000010689
	Ensembl release 38 'pep.abinitio'	GENSCAN00000012176
	Ensembl release 38 'pep.abinitio'	GENSCAN00000013713
	Ensembl release 38 'pep.abinitio'	GENSCAN00000014011
	Ensembl release 38 'pep.abinitio'	GENSCAN00000014752
	Ensembl release 38 'pep.abinitio'	GENSCAN00000014769
	Ensembl release 38 'pep.abinitio'	GENSCAN00000015669
	Ensembl release 38 'pep.abinitio'	GENSCAN00000017287
	Ensembl release 38 'pep.abinitio'	GENSCAN00000023308
	Ensembl release 38 'pep.abinitio'	GENSCAN00000024449
	Ensembl release 38 'pep.abinitio'	GENSCAN00000024873
	Ensembl release 38 'pep.abinitio'	GENSCAN00000026388
	Ensembl release 38 'pep.abinitio'	GENSCAN00000027324
	Ensembl release 38 'pep.abinitio'	GENSCAN00000028655
	Ensembl release 38 'pep.abinitio'	GENSCAN00000036588
	Ensembl release 38 'pep.abinitio'	GENSCAN00000037261
	Ensembl release 38 'pep.abinitio'	GENSCAN00000037282
	Ensembl release 38 'pep.abinitio'	GENSCAN00000043194
	Ensembl release 38 'pep.abinitio'	GENSCAN00000043195
	Ensembl release 38 'pep.abinitio'	GENSCAN00000043329
	Ensembl release 38 'pep.abinitio'	GENSCAN00000047098
D. rerio (zebrafish)	Ensembl release 38 'pep'	ENSDARP00000014779
	Ensembl release 38 'pep'	ENSDARP00000015475
	Ensembl release 38 'pep'	ENSDARP00000020711
	Ensembl release 38 'pep'	ENSDARP00000028819
	Ensembl release 38 'pep'	ENSDARP00000033635
	Ensembl release 38 'pep'	ENSDARP00000036558
	Ensembl release 38 'pep'	ENSDARP00000049952
	Ensembl release 38 'pep'	ENSDARP00000052310

	Ensembl release 38 'pep'	ENSDARP00000054686
	Ensembl release 38 'pep'	ENSDARP00000055005
	Ensembl release 38 'pep'	ENSDARP00000055023
	Ensembl release 38 'pep'	ENSDARP00000055139
	Ensembl release 38 'pep'	ENSDARP00000055953
	Ensembl release 38 'pep'	ENSDARP00000060141
	Ensembl release 38 'pep'	ENSDARP00000060154
	Ensembl release 38 'pep'	ENSDARP00000060260
	Ensembl release 38 'pep'	ENSDARP00000060334
	Ensembl release 38 'pep'	ENSDARP00000060336
	Ensembl release 38 'pep'	ENSDARP00000063175
	Ensembl release 38 'pep'	ENSDARP00000065175
	Ensembl release 38 'pep'	ENSDARP00000065228
	Ensembl release 38 'pep'	ENSDARP00000065229
	Ensembl release 38 'pep'	ENSDARP00000065339
F. rubripes (Japanese pufferfish)	Ensembl release 38 'pep.abinitio'	GENSCANSLICE00000000270
	Ensembl release 38 'pep.abinitio'	GENSCANSLICE00000008960
	Ensembl release 38 'pep.abinitio'	GENSCANSLICE00000010582
	Ensembl release 38 'pep.abinitio'	GENSCANSLICE00000011064
	Ensembl release 38 'pep.abinitio'	GENSCANSLICE00000013973
	Ensembl release 38 'pep.abinitio'	GENSCANSLICE00000015159
	Ensembl release 38 'pep.abinitio'	GENSCANSLICE00000015886
	Ensembl release 38 'pep.abinitio'	GENSCANSLICE00000016442
	Ensembl release 38 'pep.abinitio'	GENSCANSLICE00000018187
	Ensembl release 38 'pep.abinitio'	GENSCANSLICE00000018608
	Ensembl release 38 'pep.abinitio'	GENSCANSLICE00000019619
	Ensembl release 38 'pep.abinitio'	GENSCANSLICE00000019966
	Ensembl release 38 'pep.abinitio'	GENSCANSLICE00000020185
	Ensembl release 38 'pep.abinitio'	GENSCANSLICE00000020705
	Ensembl release 38 'pep.abinitio'	GENSCANSLICE00000025925
	Ensembl release 38 'pep.abinitio'	GENSCANSLICE00000006602
F. rubripes (Japanese pufferfish)	Ensembl release 38 'pep'	NEWSINFRUP00000131291
	Ensembl release 38 'pep'	NEWSINFRUP00000131701
	Ensembl release 38 'pep'	NEWSINFRUP00000135045
	Ensembl release 38 'pep'	NEWSINFRUP00000138215
	Ensembl release 38 'pep'	NEWSINFRUP00000138634
	Ensembl release 38 'pep'	NEWSINFRUP00000143580
	Ensembl release 38 'pep'	NEWSINFRUP00000144019
	Ensembl release 38 'pep'	NEWSINFRUP00000146386
	Ensembl release 38 'pep'	NEWSINFRUP00000147061
	Ensembl release 38 'pep'	NEWSINFRUP00000157320
	Ensembl release 38 'pep'	NEWSINFRUP00000163648

	Ensembl release 38 'pep'	NEWSINFRUP00000167618
	Ensembl release 38 'pep'	NEWSINFRUP00000168556
	Ensembl release 38 'pep'	NEWSINFRUP00000168996
	Ensembl release 38 'pep'	NEWSINFRUP00000177377
	Ensembl release 38 'pep'	NEWSINFRUP00000181961
T. nigroviridis (green pufferfish)	Ensembl release 38 'pep.abinitio'	GIDT00005317001
	Ensembl release 38 'pep.abinitio'	GSCT00004118001
	Ensembl release 38 'pep.abinitio'	GSCT00004170001
	Ensembl release 38 'pep.abinitio'	GSCT00004243001
	Ensembl release 38 'pep.abinitio'	GSCT00004780001
	Ensembl release 38 'pep.abinitio'	GSCT00005162001
	Ensembl release 38 'pep.abinitio'	GSCT00005835001
	Ensembl release 38 'pep.abinitio'	GSCT00008449001
	Ensembl release 38 'pep.abinitio'	GSCT00010961001
	Ensembl release 38 'pep.abinitio'	GSCT00012623001
	Ensembl release 38 'pep.abinitio'	GSCT00012677001
	Ensembl release 38 'pep.abinitio'	GSCT00013311001
	Ensembl release 38 'pep.abinitio'	GSCT00022847001
	Ensembl release 38 'pep.abinitio'	GSCT00026074001
	Ensembl release 38 'pep.abinitio'	GWSHT00009051001
	Ensembl release 38 'pep.abinitio'	GWSHT00010289001
	Ensembl release 38 'pep.abinitio'	GWSST00001947001
	Ensembl release 38 'pep.abinitio'	GWSST00010292001
	Ensembl release 38 'pep.abinitio'	GWSST00001338001
	Ensembl release 38 'pep.abinitio'	GWSST00009038001
T. nigroviridis (green pufferfish)	Ensembl release 38 'pep'	GSTENP00009678001
	Ensembl release 38 'pep'	GSTENP00010088001
	Ensembl release 38 'pep'	GSTENP00011314001
	Ensembl release 38 'pep'	GSTENP00011330001
	Ensembl release 38 'pep'	GSTENP00013690001
	Ensembl release 38 'pep'	GSTENP00015613001
	Ensembl release 38 'pep'	GSTENP00021168001
	Ensembl release 38 'pep'	GSTENP00023920001
	Ensembl release 38 'pep'	GSTENP00025477001
	Ensembl release 38 'pep'	GSTENP00025576001
	Ensembl release 38 'pep'	GSTENP00025914001
	Ensembl release 38 'pep'	GSTENP00026075001
	Ensembl release 38 'pep'	GSTENP00026551001
	Ensembl release 38 'pep'	GSTENP00027188001
	Ensembl release 38 'pep'	GSTENP00027960001
	Ensembl release 38 'pep'	GSTENP00029184001
	Ensembl release 38 'pep'	GSTENP00037186001

B. floridae (amphioxus)	JGI *B. floridae* v1.0 annotation	e_gw.1.508.1
	JGI *B. floridae* v1.0 annotation	e_gw.102.129.1
	JGI *B. floridae* v1.0 annotation	e_gw.102.131.1
	JGI *B. floridae* v1.0 annotation	e_gw.102.45.1
	JGI *B. floridae* v1.0 annotation	e_gw.1052.5.1
	JGI *B. floridae* v1.0 annotation	e_gw.106.152.1
	JGI *B. floridae* v1.0 annotation	e_gw.239.19.1
	JGI *B. floridae* v1.0 annotation	e_gw.242.16.1
	JGI *B. floridae* v1.0 annotation	e_gw.267.25.1
	JGI *B. floridae* v1.0 annotation	e_gw.287.27.1
	JGI *B. floridae* v1.0 annotation	e_gw.344.14.1
	JGI *B. floridae* v1.0 annotation	e_gw.344.49.1
	JGI *B. floridae* v1.0 annotation	e_gw.371.22.1
	JGI *B. floridae* v1.0 annotation	e_gw.39.83.1
	JGI *B. floridae* v1.0 annotation	e_gw.400.24.1
	JGI *B. floridae* v1.0 annotation	e_gw.41.211.1
	JGI *B. floridae* v1.0 annotation	e_gw.41.55.1
	JGI *B. floridae* v1.0 annotation	e_gw.439.11.1
	JGI *B. floridae* v1.0 annotation	e_gw.439.14.1
	JGI *B. floridae* v1.0 annotation	e_gw.47.58.1
	JGI *B. floridae* v1.0 annotation	e_gw.486.11.1
	JGI *B. floridae* v1.0 annotation	e_gw.54.171.1
	JGI *B. floridae* v1.0 annotation	e_gw.54.3.1
	JGI *B. floridae* v1.0 annotation	e_gw.56.121.1
	JGI *B. floridae* v1.0 annotation	e_gw.56.214.1
	JGI *B. floridae* v1.0 annotation	e_gw.639.4.1
	JGI *B. floridae* v1.0 annotation	e_gw.75.185.1
	JGI *B. floridae* v1.0 annotation	e_gw.75.188.1
	JGI *B. floridae* v1.0 annotation	e_gw.75.189.1
	JGI *B. floridae* v1.0 annotation	e_gw.75.190.1
	JGI *B. floridae* v1.0 annotation	e_gw.75.49.1
	JGI *B. floridae* v1.0 annotation	e_gw.75.54.1
	JGI *B. floridae* v1.0 annotation	e_gw.767.15.1
	JGI *B. floridae* v1.0 annotation	estExt_fgenesh2_pg.C_1850079
	JGI *B. floridae* v1.0 annotation	estExt_fgenesh2_pg.C_2060045
	JGI *B. floridae* v1.0 annotation	estExt_fgenesh2_pg.C_2140015
	JGI *B. floridae* v1.0 annotation	estExt_fgenesh2_pg.C_750028
	JGI *B. floridae* v1.0 annotation	fgenesh2_pg.scaffold_1000237
	JGI *B. floridae* v1.0 annotation	fgenesh2_pg.scaffold_114000017
	JGI *B. floridae* v1.0 annotation	fgenesh2_pg.scaffold_134000033
	JGI *B. floridae* v1.0 annotation	fgenesh2_pg.scaffold_144000044
	JGI *B. floridae* v1.0 annotation	fgenesh2_pg.scaffold_1466000001
	JGI *B. floridae* v1.0 annotation	fgenesh2_pg.scaffold_163000047

JGI *B. floridae* v1.0 annotation	fgenesh2_pg.scaffold_163000048
JGI *B. floridae* v1.0 annotation	fgenesh2_pg.scaffold_163000054
JGI *B. floridae* v1.0 annotation	fgenesh2_pg.scaffold_163000065
JGI *B. floridae* v1.0 annotation	fgenesh2_pg.scaffold_174000015
JGI *B. floridae* v1.0 annotation	fgenesh2_pg.scaffold_175000015
JGI *B. floridae* v1.0 annotation	fgenesh2_pg.scaffold_177000008
JGI *B. floridae* v1.0 annotation	fgenesh2_pg.scaffold_18000081
JGI *B. floridae* v1.0 annotation	fgenesh2_pg.scaffold_18000082
JGI *B. floridae* v1.0 annotation	fgenesh2_pg.scaffold_18000083
JGI *B. floridae* v1.0 annotation	fgenesh2_pg.scaffold_187000032
JGI *B. floridae* v1.0 annotation	fgenesh2_pg.scaffold_187000033
JGI *B. floridae* v1.0 annotation	fgenesh2_pg.scaffold_187000076
JGI *B. floridae* v1.0 annotation	fgenesh2_pg.scaffold_187000078
JGI *B. floridae* v1.0 annotation	fgenesh2_pg.scaffold_187000079
JGI *B. floridae* v1.0 annotation	fgenesh2_pg.scaffold_188000027
JGI *B. floridae* v1.0 annotation	fgenesh2_pg.scaffold_189000035
JGI *B. floridae* v1.0 annotation	fgenesh2_pg.scaffold_19000069
JGI *B. floridae* v1.0 annotation	fgenesh2_pg.scaffold_19000170
JGI *B. floridae* v1.0 annotation	fgenesh2_pg.scaffold_2000287
JGI *B. floridae* v1.0 annotation	fgenesh2_pg.scaffold_206000046
JGI *B. floridae* v1.0 annotation	fgenesh2_pg.scaffold_213000033
JGI *B. floridae* v1.0 annotation	fgenesh2_pg.scaffold_22000045
JGI *B. floridae* v1.0 annotation	fgenesh2_pg.scaffold_22000061
JGI *B. floridae* v1.0 annotation	fgenesh2_pg.scaffold_230000024
JGI *B. floridae* v1.0 annotation	fgenesh2_pg.scaffold_255000034
JGI *B. floridae* v1.0 annotation	fgenesh2_pg.scaffold_287000037
JGI *B. floridae* v1.0 annotation	fgenesh2_pg.scaffold_334000015
JGI *B. floridae* v1.0 annotation	fgenesh2_pg.scaffold_361000032
JGI *B. floridae* v1.0 annotation	fgenesh2_pg.scaffold_366000022
JGI *B. floridae* v1.0 annotation	fgenesh2_pg.scaffold_401000022
JGI *B. floridae* v1.0 annotation	fgenesh2_pg.scaffold_43000097
JGI *B. floridae* v1.0 annotation	fgenesh2_pg.scaffold_48000071
JGI *B. floridae* v1.0 annotation	fgenesh2_pg.scaffold_489000023
JGI *B. floridae* v1.0 annotation	fgenesh2_pg.scaffold_61000179
JGI *B. floridae* v1.0 annotation	fgenesh2_pg.scaffold_7000217
JGI *B. floridae* v1.0 annotation	fgenesh2_pg.scaffold_75000003
JGI *B. floridae* v1.0 annotation	fgenesh2_pg.scaffold_76000029
JGI *B. floridae* v1.0 annotation	fgenesh2_pg.scaffold_90000004
JGI *B. floridae* v1.0 annotation	fgenesh2_pm.scaffold_18000015
JGI *B. floridae* v1.0 annotation	fgenesh2_pm.scaffold_356000002
JGI *B. floridae* v1.0 annotation	gw.102.43.1
JGI *B. floridae* v1.0 annotation	gw.106.131.1
JGI *B. floridae* v1.0 annotation	gw.106.50.1

	JGI *B. floridae* v1.0 annotation	gw.239.18.1
	JGI *B. floridae* v1.0 annotation	gw.239.44.1
	JGI *B. floridae* v1.0 annotation	gw.25.174.1
	JGI *B. floridae* v1.0 annotation	gw.264.29.1
	JGI *B. floridae* v1.0 annotation	gw.32.126.1
	JGI *B. floridae* v1.0 annotation	gw.356.28.1
	JGI *B. floridae* v1.0 annotation	gw.356.82.1
	JGI *B. floridae* v1.0 annotation	gw.391.9.1
	JGI *B. floridae* v1.0 annotation	gw.435.44.1
	JGI *B. floridae* v1.0 annotation	gw.44.60.1
	JGI *B. floridae* v1.0 annotation	gw.675.12.1
	JGI *B. floridae* v1.0 annotation	gw.688.12.1
	JGI *B. floridae* v1.0 annotation	gw.75.193.1
	JGI *B. floridae* v1.0 annotation	gw.784.7.1
	JGI *B. floridae* v1.0 annotation	gw.81.36.1
	JGI *B. floridae* v1.0 annotation	gw.839.2.1
	JGI *B. floridae* v1.0 annotation	e_gw.470.10.1
	JGI *B. floridae* v1.0 annotation	fgenesh2_pg.scaffold_130000118
	JGI *B. floridae* v1.0 annotation	fgenesh2_pg.scaffold_137000036
	JGI *B. floridae* v1.0 annotation	fgenesh2_pg.scaffold_272000001
	JGI *B. floridae* v1.0 annotation	fgenesh2_pg.scaffold_126000025
	JGI *B. floridae* v1.0 annotation	e_gw.75.192.1
	JGI *B. floridae* v1.0 annotation	e_gw.75.60.1
	JGI *B. floridae* v1.0 annotation	fgenesh2_pg.scaffold_73000085
	JGI *B. floridae* v1.0 annotation	fgenesh2_pg.scaffold_1000239
	JGI *B. floridae* v1.0 annotation	fgenesh2_pg.scaffold_410000011
	JGI *B. floridae* v1.0 annotation	estExt_fgenesh2_pg.C_2670041
	JGI *B. floridae* v1.0 annotation	fgenesh2_pg.scaffold_235000018
	JGI *B. floridae* v1.0 annotation	e_gw.334.50.1
	JGI *B. floridae* v1.0 annotation	estExt_fgenesh2_pg.C_1340031
	JGI *B. floridae* v1.0 annotation	fgenesh2_pg.scaffold_186000038
	JGI *B. floridae* v1.0 annotation	estExt_fgenesh2_pg.C_5050026
	JGI *B. floridae* v1.0 annotation	fgenesh2_pg.scaffold_122000096
	JGI *B. floridae* v1.0 annotation	fgenesh2_pg.scaffold_303000054
	JGI *B. floridae* v1.0 annotation	fgenesh2_pg.scaffold_110000023
	JGI *B. floridae* v1.0 annotation	fgenesh2_pg.scaffold_599000007
	JGI *B. floridae* v1.0 annotation	e_gw.334.6.1
	JGI *B. floridae* v1.0 annotation	fgenesh2_pg.scaffold_218000013
	JGI *B. floridae* v1.0 annotation	fgenesh2_pg.scaffold_170000017
C. intestinalis (transparent sea squirt)	Ensembl release 38 'pep.abinitio'	GENSCAN00000083317
	Ensembl release 38 'pep.abinitio'	GENSCAN00000086824
	Ensembl release 38 'pep.abinitio'	GENSCAN00000100475
	Ensembl release 38 'pep.abinitio'	GENSCAN00000096598

C. intestinalis (transparent sea squirt)	Ensembl release 38 'pep'	ENSCINP00000000241
	Ensembl release 38 'pep'	ENSCINP00000024401
S. purpuratus (purple sea urchin)	HGSC Spur_v2.0 assembly (genscan result)	Scaffold_v2_10491_genscan_1
	HGSC Spur_v2.0 assembly (genscan result)	Scaffold_v2_11325_genscan_2
	HGSC Spur_v2.0 assembly (genscan result)	Scaffold_v2_11879_genscan_1
	HGSC Spur_v2.0 assembly (genscan result)	Scaffold_v2_12153_genscan_22
	HGSC Spur_v2.0 assembly (genscan result)	Scaffold_v2_12153_genscan_19
	HGSC Spur_v2.0 assembly (genscan result)	Scaffold_v2_12161_genscan_5
	HGSC Spur_v2.0 assembly (genscan result)	Scaffold_v2_12739_genscan_4
	HGSC Spur_v2.0 assembly (genscan result)	Scaffold_v2_13740_genscan_4
	HGSC Spur_v2.0 assembly (genscan result)	Scaffold_v2_14069_genscan_1
	HGSC Spur_v2.0 assembly (genscan result)	Scaffold_v2_14511_genscan_8
	HGSC Spur_v2.0 assembly (genscan result)	Scaffold_v2_14513_genscan_14
	HGSC Spur_v2.0 assembly (genscan result)	Scaffold_v2_14513_genscan_16
	HGSC Spur_v2.0 assembly (genscan result)	Scaffold_v2_15422_genscan_1
	HGSC Spur_v2.0 assembly (genscan result)	Scaffold_v2_15453_genscan_3
	HGSC Spur_v2.0 assembly (genscan result)	Scaffold_v2_15454_genscan_13
	HGSC Spur_v2.0 assembly (genscan result)	Scaffold_v2_15504_genscan_2
	HGSC Spur_v2.0 assembly (genscan result)	Scaffold_v2_15504_genscan_3
	HGSC Spur_v2.0 assembly (genscan result)	Scaffold_v2_15523_genscan_2
	HGSC Spur_v2.0 assembly (genscan result)	Scaffold_v2_15579_genscan_1
	HGSC Spur_v2.0 assembly (genscan result)	Scaffold_v2_16324_genscan_5
	HGSC Spur_v2.0 assembly (genscan result)	Scaffold_v2_16931_genscan_1
	HGSC Spur_v2.0 assembly (genscan result)	Scaffold_v2_17238_genscan_2
	HGSC Spur_v2.0 assembly (genscan result)	Scaffold_v2_17883_genscan_1
	HGSC Spur_v2.0 assembly (genscan result)	Scaffold_v2_17907_genscan_1
	HGSC Spur_v2.0 assembly (genscan result)	Scaffold_v2_18026_genscan_3
	HGSC Spur_v2.0 assembly (genscan result)	Scaffold_v2_18121_genscan_2
	HGSC Spur_v2.0 assembly (genscan result)	Scaffold_v2_18964_genscan_21
	HGSC Spur_v2.0 assembly (genscan result)	Scaffold_v2_18964_genscan_23
	HGSC Spur_v2.0 assembly (genscan result)	Scaffold_v2_18964_genscan_25
	HGSC Spur_v2.0 assembly (genscan result)	Scaffold_v2_19932_genscan_3
	HGSC Spur_v2.0 assembly (genscan result)	Scaffold_v2_19973_genscan_2
	HGSC Spur_v2.0 assembly (genscan result)	Scaffold_v2_21540_genscan_2
	HGSC Spur_v2.0 assembly (genscan result)	Scaffold_v2_21540_genscan_3
	HGSC Spur_v2.0 assembly (genscan result)	Scaffold_v2_21551_genscan_2
	HGSC Spur_v2.0 assembly (genscan result)	Scaffold_v2_22311_genscan_2
	HGSC Spur_v2.0 assembly (genscan result)	Scaffold_v2_22311_genscan_3
	HGSC Spur_v2.0 assembly (genscan result)	Scaffold_v2_22311_genscan_4
	HGSC Spur_v2.0 assembly (genscan result)	Scaffold_v2_22325_genscan_2
	HGSC Spur_v2.0 assembly (genscan result)	Scaffold_v2_22325_genscan_3
	HGSC Spur_v2.0 assembly (genscan result)	Scaffold_v2_23011_genscan_3

HGSC Spur_v2.0 assembly (genscan result)	Scaffold_v2_2331_genscan_2
HGSC Spur_v2.0 assembly (genscan result)	Scaffold_v2_2339_genscan_1
HGSC Spur_v2.0 assembly (genscan result)	Scaffold_v2_23980_genscan_1
HGSC Spur_v2.0 assembly (genscan result)	Scaffold_v2_24692_genscan_3
HGSC Spur_v2.0 assembly (genscan result)	Scaffold_v2_25560_genscan_10
HGSC Spur_v2.0 assembly (genscan result)	Scaffold_v2_25560_genscan_11
HGSC Spur_v2.0 assembly (genscan result)	Scaffold_v2_25593_genscan_3
HGSC Spur_v2.0 assembly (genscan result)	Scaffold_v2_26354_genscan_11
HGSC Spur_v2.0 assembly (genscan result)	Scaffold_v2_26354_genscan_12
HGSC Spur_v2.0 assembly (genscan result)	Scaffold_v2_26363_genscan_6
HGSC Spur_v2.0 assembly (genscan result)	Scaffold_v2_26382_genscan_1
HGSC Spur_v2.0 assembly (genscan result)	Scaffold_v2_26382_genscan_2
HGSC Spur_v2.0 assembly (genscan result)	Scaffold_v2_26993_genscan_1
HGSC Spur_v2.0 assembly (genscan result)	Scaffold_v2_27018_genscan_1
HGSC Spur_v2.0 assembly (genscan result)	Scaffold_v2_28746_genscan_1
HGSC Spur_v2.0 assembly (genscan result)	Scaffold_v2_28746_genscan_3
HGSC Spur_v2.0 assembly (genscan result)	Scaffold_v2_28746_genscan_5
HGSC Spur_v2.0 assembly (genscan result)	Scaffold_v2_28746_genscan_7
HGSC Spur_v2.0 assembly (genscan result)	Scaffold_v2_28754_genscan_3
HGSC Spur_v2.0 assembly (genscan result)	Scaffold_v2_28833_genscan_1
HGSC Spur_v2.0 assembly (genscan result)	Scaffold_v2_30645_genscan_2
HGSC Spur_v2.0 assembly (genscan result)	Scaffold_v2_32372_genscan_2
HGSC Spur_v2.0 assembly (genscan result)	Scaffold_v2_32421_genscan_1
HGSC Spur_v2.0 assembly (genscan result)	Scaffold_v2_33267_genscan_1
HGSC Spur_v2.0 assembly (genscan result)	Scaffold_v2_3400_genscan_1
HGSC Spur_v2.0 assembly (genscan result)	Scaffold_v2_3400_genscan_9
HGSC Spur_v2.0 assembly (genscan result)	Scaffold_v2_3400_genscan_10
HGSC Spur_v2.0 assembly (genscan result)	Scaffold_v2_3400_genscan_11
HGSC Spur_v2.0 assembly (genscan result)	Scaffold_v2_3400_genscan_12
HGSC Spur_v2.0 assembly (genscan result)	Scaffold_v2_3400_genscan_13
HGSC Spur_v2.0 assembly (genscan result)	Scaffold_v2_3400_genscan_15
HGSC Spur_v2.0 assembly (genscan result)	Scaffold_v2_3400_genscan_21
HGSC Spur_v2.0 assembly (genscan result)	Scaffold_v2_3400_genscan_24
HGSC Spur_v2.0 assembly (genscan result)	Scaffold_v2_34023_genscan_50
HGSC Spur_v2.0 assembly (genscan result)	Scaffold_v2_34023_genscan_52
HGSC Spur_v2.0 assembly (genscan result)	Scaffold_v2_3407_genscan_5
HGSC Spur_v2.0 assembly (genscan result)	Scaffold_v2_3407_genscan_9
HGSC Spur_v2.0 assembly (genscan result)	Scaffold_v2_34693_genscan_25
HGSC Spur_v2.0 assembly (genscan result)	Scaffold_v2_35416_genscan_7
HGSC Spur_v2.0 assembly (genscan result)	Scaffold_v2_35416_genscan_8
HGSC Spur_v2.0 assembly (genscan result)	Scaffold_v2_35416_genscan_20
HGSC Spur_v2.0 assembly (genscan result)	Scaffold_v2_35445_genscan_2
HGSC Spur_v2.0 assembly (genscan result)	Scaffold_v2_35888_genscan_1

HGSC Spur_v2.0 assembly (genscan result)	Scaffold_v2_36376_genscan_3
HGSC Spur_v2.0 assembly (genscan result)	Scaffold_v2_37278_genscan_2
HGSC Spur_v2.0 assembly (genscan result)	Scaffold_v2_38348_genscan_2
HGSC Spur_v2.0 assembly (genscan result)	Scaffold_v2_40154_genscan_1
HGSC Spur_v2.0 assembly (genscan result)	Scaffold_v2_40613_genscan_1
HGSC Spur_v2.0 assembly (genscan result)	Scaffold_v2_40959_genscan_1
HGSC Spur_v2.0 assembly (genscan result)	Scaffold_v2_41062_genscan_1
HGSC Spur_v2.0 assembly (genscan result)	Scaffold_v2_41614_genscan_6
HGSC Spur_v2.0 assembly (genscan result)	Scaffold_v2_41618_genscan_3
HGSC Spur_v2.0 assembly (genscan result)	Scaffold_v2_41626_genscan_5
HGSC Spur_v2.0 assembly (genscan result)	Scaffold_v2_42254_genscan_1
HGSC Spur_v2.0 assembly (genscan result)	Scaffold_v2_42464_genscan_3
HGSC Spur_v2.0 assembly (genscan result)	Scaffold_v2_42593_genscan_1
HGSC Spur_v2.0 assembly (genscan result)	Scaffold_v2_43226_genscan_1
HGSC Spur_v2.0 assembly (genscan result)	Scaffold_v2_4371_genscan_1
HGSC Spur_v2.0 assembly (genscan result)	Scaffold_v2_44502_genscan_7
HGSC Spur_v2.0 assembly (genscan result)	Scaffold_v2_45219_genscan_4
HGSC Spur_v2.0 assembly (genscan result)	Scaffold_v2_45223_genscan_3
HGSC Spur_v2.0 assembly (genscan result)	Scaffold_v2_46931_genscan_1
HGSC Spur_v2.0 assembly (genscan result)	Scaffold_v2_47461_genscan_26
HGSC Spur_v2.0 assembly (genscan result)	Scaffold_v2_47461_genscan_28
HGSC Spur_v2.0 assembly (genscan result)	Scaffold_v2_47491_genscan_2
HGSC Spur_v2.0 assembly (genscan result)	Scaffold_v2_48167_genscan_4
HGSC Spur_v2.0 assembly (genscan result)	Scaffold_v2_48979_genscan_6
HGSC Spur_v2.0 assembly (genscan result)	Scaffold_v2_4912_genscan_3
HGSC Spur_v2.0 assembly (genscan result)	Scaffold_v2_4912_genscan_5
HGSC Spur_v2.0 assembly (genscan result)	Scaffold_v2_4914_genscan_4
HGSC Spur_v2.0 assembly (genscan result)	Scaffold_v2_50246_genscan_3
HGSC Spur_v2.0 assembly (genscan result)	Scaffold_v2_51797_genscan_3
HGSC Spur_v2.0 assembly (genscan result)	Scaffold_v2_52389_genscan_1
HGSC Spur_v2.0 assembly (genscan result)	Scaffold_v2_52482_genscan_4
HGSC Spur_v2.0 assembly (genscan result)	Scaffold_v2_52597_genscan_2
HGSC Spur_v2.0 assembly (genscan result)	Scaffold_v2_53363_genscan_10
HGSC Spur_v2.0 assembly (genscan result)	Scaffold_v2_53478_genscan_1
HGSC Spur_v2.0 assembly (genscan result)	Scaffold_v2_54084_genscan_1
HGSC Spur_v2.0 assembly (genscan result)	Scaffold_v2_54273_genscan_5
HGSC Spur_v2.0 assembly (genscan result)	Scaffold_v2_54273_genscan_6
HGSC Spur_v2.0 assembly (genscan result)	Scaffold_v2_55223_genscan_8
HGSC Spur_v2.0 assembly (genscan result)	Scaffold_v2_5551_genscan_1
HGSC Spur_v2.0 assembly (genscan result)	Scaffold_v2_55778_genscan_1
HGSC Spur_v2.0 assembly (genscan result)	Scaffold_v2_56611_genscan_14
HGSC Spur_v2.0 assembly (genscan result)	Scaffold_v2_56611_genscan_16
HGSC Spur_v2.0 assembly (genscan result)	Scaffold_v2_57319_genscan_8

HGSC Spur_v2.0 assembly (genscan result)	Scaffold_v2_57393_genscan_4
HGSC Spur_v2.0 assembly (genscan result)	Scaffold_v2_57993_genscan_1
HGSC Spur_v2.0 assembly (genscan result)	Scaffold_v2_57995_genscan_3
HGSC Spur_v2.0 assembly (genscan result)	Scaffold_v2_5811_genscan_5
HGSC Spur_v2.0 assembly (genscan result)	Scaffold_v2_58784_genscan_4
HGSC Spur_v2.0 assembly (genscan result)	Scaffold_v2_58795_genscan_6
HGSC Spur_v2.0 assembly (genscan result)	Scaffold_v2_58814_genscan_4
HGSC Spur_v2.0 assembly (genscan result)	Scaffold_v2_58831_genscan_2
HGSC Spur_v2.0 assembly (genscan result)	Scaffold_v2_58831_genscan_6
HGSC Spur_v2.0 assembly (genscan result)	Scaffold_v2_58834_genscan_1
HGSC Spur_v2.0 assembly (genscan result)	Scaffold_v2_5886_genscan_1
HGSC Spur_v2.0 assembly (genscan result)	Scaffold_v2_59506_genscan_1
HGSC Spur_v2.0 assembly (genscan result)	Scaffold_v2_59537_genscan_1
HGSC Spur_v2.0 assembly (genscan result)	Scaffold_v2_59600_genscan_2
HGSC Spur_v2.0 assembly (genscan result)	Scaffold_v2_5975_genscan_4
HGSC Spur_v2.0 assembly (genscan result)	Scaffold_v2_60771_genscan_1
HGSC Spur_v2.0 assembly (genscan result)	Scaffold_v2_61298_genscan_8
HGSC Spur_v2.0 assembly (genscan result)	Scaffold_v2_61525_genscan_1
HGSC Spur_v2.0 assembly (genscan result)	Scaffold_v2_62076_genscan_8
HGSC Spur_v2.0 assembly (genscan result)	Scaffold_v2_62987_genscan_1
HGSC Spur_v2.0 assembly (genscan result)	Scaffold_v2_63638_genscan_1
HGSC Spur_v2.0 assembly (genscan result)	Scaffold_v2_63638_genscan_6
HGSC Spur_v2.0 assembly (genscan result)	Scaffold_v2_63638_genscan_22
HGSC Spur_v2.0 assembly (genscan result)	Scaffold_v2_63638_genscan_25
HGSC Spur_v2.0 assembly (genscan result)	Scaffold_v2_63638_genscan_26
HGSC Spur_v2.0 assembly (genscan result)	Scaffold_v2_6416_genscan_11
HGSC Spur_v2.0 assembly (genscan result)	Scaffold_v2_6451_genscan_1
HGSC Spur_v2.0 assembly (genscan result)	Scaffold_v2_64559_genscan_2
HGSC Spur_v2.0 assembly (genscan result)	Scaffold_v2_64564_genscan_1
HGSC Spur_v2.0 assembly (genscan result)	Scaffold_v2_6465_genscan_1
HGSC Spur_v2.0 assembly (genscan result)	Scaffold_v2_6488_genscan_2
HGSC Spur_v2.0 assembly (genscan result)	Scaffold_v2_65202_genscan_1
HGSC Spur_v2.0 assembly (genscan result)	Scaffold_v2_67435_genscan_1
HGSC Spur_v2.0 assembly (genscan result)	Scaffold_v2_67840_genscan_1
HGSC Spur_v2.0 assembly (genscan result)	Scaffold_v2_68_genscan_3
HGSC Spur_v2.0 assembly (genscan result)	Scaffold_v2_68041_genscan_2
HGSC Spur_v2.0 assembly (genscan result)	Scaffold_v2_68317_genscan_1
HGSC Spur_v2.0 assembly (genscan result)	Scaffold_v2_68317_genscan_2
HGSC Spur_v2.0 assembly (genscan result)	Scaffold_v2_68397_genscan_15
HGSC Spur_v2.0 assembly (genscan result)	Scaffold_v2_70729_genscan_2
HGSC Spur_v2.0 assembly (genscan result)	Scaffold_v2_71913_genscan_12
HGSC Spur_v2.0 assembly (genscan result)	Scaffold_v2_71945_genscan_1
HGSC Spur_v2.0 assembly (genscan result)	Scaffold_v2_74946_genscan_17

HGSC Spur_v2.0 assembly (genscan result)	Scaffold_v2_74946_genscan_18
HGSC Spur_v2.0 assembly (genscan result)	Scaffold_v2_74946_genscan_19
HGSC Spur_v2.0 assembly (genscan result)	Scaffold_v2_74946_genscan_20
HGSC Spur_v2.0 assembly (genscan result)	Scaffold_v2_74946_genscan_22
HGSC Spur_v2.0 assembly (genscan result)	Scaffold_v2_74946_genscan_23
HGSC Spur_v2.0 assembly (genscan result)	Scaffold_v2_74946_genscan_24
HGSC Spur_v2.0 assembly (genscan result)	Scaffold_v2_74946_genscan_29
HGSC Spur_v2.0 assembly (genscan result)	Scaffold_v2_74946_genscan_31
HGSC Spur_v2.0 assembly (genscan result)	Scaffold_v2_75062_genscan_1
HGSC Spur_v2.0 assembly (genscan result)	Scaffold_v2_75802_genscan_2
HGSC Spur_v2.0 assembly (genscan result)	Scaffold_v2_75802_genscan_5
HGSC Spur_v2.0 assembly (genscan result)	Scaffold_v2_76986_genscan_2
HGSC Spur_v2.0 assembly (genscan result)	Scaffold_v2_78130_genscan_1
HGSC Spur_v2.0 assembly (genscan result)	Scaffold_v2_79447_genscan_2
HGSC Spur_v2.0 assembly (genscan result)	Scaffold_v2_80295_genscan_2
HGSC Spur_v2.0 assembly (genscan result)	Scaffold_v2_80669_genscan_1
HGSC Spur_v2.0 assembly (genscan result)	Scaffold_v2_8113_genscan_1
HGSC Spur_v2.0 assembly (genscan result)	Scaffold_v2_81714_genscan_3
HGSC Spur_v2.0 assembly (genscan result)	Scaffold_v2_81725_genscan_8
HGSC Spur_v2.0 assembly (genscan result)	Scaffold_v2_81778_genscan_1
HGSC Spur_v2.0 assembly (genscan result)	Scaffold_v2_81826_genscan_2
HGSC Spur_v2.0 assembly (genscan result)	Scaffold_v2_82277_genscan_2
HGSC Spur_v2.0 assembly (genscan result)	Scaffold_v2_82277_genscan_3
HGSC Spur_v2.0 assembly (genscan result)	Scaffold_v2_83189_genscan_1
HGSC Spur_v2.0 assembly (genscan result)	Scaffold_v2_83307_genscan_1
HGSC Spur_v2.0 assembly (genscan result)	Scaffold_v2_83337_genscan_1
HGSC Spur_v2.0 assembly (genscan result)	Scaffold_v2_83907_genscan_9
HGSC Spur_v2.0 assembly (genscan result)	Scaffold_v2_84556_genscan_1
HGSC Spur_v2.0 assembly (genscan result)	Scaffold_v2_85337_genscan_10
HGSC Spur_v2.0 assembly (genscan result)	Scaffold_v2_86602_genscan_1
HGSC Spur_v2.0 assembly (genscan result)	Scaffold_v2_87793_genscan_1
HGSC Spur_v2.0 assembly (genscan result)	Scaffold_v2_87793_genscan_36
HGSC Spur_v2.0 assembly (genscan result)	Scaffold_v2_87804_genscan_2
HGSC Spur_v2.0 assembly (genscan result)	Scaffold_v2_87804_genscan_8
HGSC Spur_v2.0 assembly (genscan result)	Scaffold_v2_88473_genscan_1
HGSC Spur_v2.0 assembly (genscan result)	Scaffold_v2_88598_genscan_2
HGSC Spur_v2.0 assembly (genscan result)	Scaffold_v2_89526_genscan_2
HGSC Spur_v2.0 assembly (genscan result)	Scaffold_v2_89526_genscan_1
HGSC Spur_v2.0 assembly (genscan result)	Scaffold_v2_8987_genscan_2
HGSC Spur_v2.0 assembly (genscan result)	Scaffold_v2_92197_genscan_1
HGSC Spur_v2.0 assembly (genscan result)	Scaffold_v2_96016_genscan_1
HGSC Spur_v2.0 assembly (genscan result)	Scaffold_v2_38360_genscan_1
HGSC Spur_v2.0 assembly (genscan result)	Scaffold_v2_46257_genscan_1

	HGSC Spur_v2.0 assembly (genscan result)	Scaffold_v2_59541_genscan_12
	HGSC Spur_v2.0 assembly (genscan result)	Scaffold_v2_59541_genscan_13
	HGSC Spur_v2.0 assembly (genscan result)	Scaffold_v2_68554_genscan_1
	HGSC Spur_v2.0 assembly (genscan result)	Scaffold_v2_74156_genscan_4
S. purpuratus (purple sea urchin)	HGSC *S. purpuratus* GLEAN3	GLEAN3_00199
	HGSC *S. purpuratus* GLEAN3	GLEAN3_00375
	HGSC *S. purpuratus* GLEAN3	GLEAN3_00615
	HGSC *S. purpuratus* GLEAN3	GLEAN3_00870
	HGSC *S. purpuratus* GLEAN3	GLEAN3_00871
	HGSC *S. purpuratus* GLEAN3	GLEAN3_00911
	HGSC *S. purpuratus* GLEAN3	GLEAN3_01458
	HGSC *S. purpuratus* GLEAN3	GLEAN3_01650
	HGSC *S. purpuratus* GLEAN3	GLEAN3_01877
	HGSC *S. purpuratus* GLEAN3	GLEAN3_01970
	HGSC *S. purpuratus* GLEAN3	GLEAN3_01971
	HGSC *S. purpuratus* GLEAN3	GLEAN3_01993
	HGSC *S. purpuratus* GLEAN3	GLEAN3_02224
	HGSC *S. purpuratus* GLEAN3	GLEAN3_02442
	HGSC *S. purpuratus* GLEAN3	GLEAN3_02538
	HGSC *S. purpuratus* GLEAN3	GLEAN3_02803
	HGSC *S. purpuratus* GLEAN3	GLEAN3_03419
	HGSC *S. purpuratus* GLEAN3	GLEAN3_03578
	HGSC *S. purpuratus* GLEAN3	GLEAN3_03579
	HGSC *S. purpuratus* GLEAN3	GLEAN3_04139
	HGSC *S. purpuratus* GLEAN3	GLEAN3_04150
	HGSC *S. purpuratus* GLEAN3	GLEAN3_04360
	HGSC *S. purpuratus* GLEAN3	GLEAN3_04655
	HGSC *S. purpuratus* GLEAN3	GLEAN3_04792
	HGSC *S. purpuratus* GLEAN3	GLEAN3_04951
	HGSC *S. purpuratus* GLEAN3	GLEAN3_04957
	HGSC *S. purpuratus* GLEAN3	GLEAN3_05088
	HGSC *S. purpuratus* GLEAN3	GLEAN3_05148
	HGSC *S. purpuratus* GLEAN3	GLEAN3_05339
	HGSC *S. purpuratus* GLEAN3	GLEAN3_05830
	HGSC *S. purpuratus* GLEAN3	GLEAN3_05832
	HGSC *S. purpuratus* GLEAN3	GLEAN3_05950
	HGSC *S. purpuratus* GLEAN3	GLEAN3_06218
	HGSC *S. purpuratus* GLEAN3	GLEAN3_06458
	HGSC *S. purpuratus* GLEAN3	GLEAN3_06939
	HGSC *S. purpuratus* GLEAN3	GLEAN3_07342
	HGSC *S. purpuratus* GLEAN3	GLEAN3_07418
	HGSC *S. purpuratus* GLEAN3	GLEAN3_07429
	HGSC *S. purpuratus* GLEAN3	GLEAN3_07430

HGSC *S. purpuratus* GLEAN3	GLEAN3_07790
HGSC *S. purpuratus* GLEAN3	GLEAN3_07850
HGSC *S. purpuratus* GLEAN3	GLEAN3_07859
HGSC *S. purpuratus* GLEAN3	GLEAN3_07986
HGSC *S. purpuratus* GLEAN3	GLEAN3_08228
HGSC *S. purpuratus* GLEAN3	GLEAN3_08267
HGSC *S. purpuratus* GLEAN3	GLEAN3_08278
HGSC *S. purpuratus* GLEAN3	GLEAN3_08396
HGSC *S. purpuratus* GLEAN3	GLEAN3_08456
HGSC *S. purpuratus* GLEAN3	GLEAN3_08962
HGSC *S. purpuratus* GLEAN3	GLEAN3_08963
HGSC *S. purpuratus* GLEAN3	GLEAN3_09037
HGSC *S. purpuratus* GLEAN3	GLEAN3_09129
HGSC *S. purpuratus* GLEAN3	GLEAN3_09173
HGSC *S. purpuratus* GLEAN3	GLEAN3_09343
HGSC *S. purpuratus* GLEAN3	GLEAN3_09435
HGSC *S. purpuratus* GLEAN3	GLEAN3_09829
HGSC *S. purpuratus* GLEAN3	GLEAN3_09933
HGSC *S. purpuratus* GLEAN3	GLEAN3_10575
HGSC *S. purpuratus* GLEAN3	GLEAN3_10619
HGSC *S. purpuratus* GLEAN3	GLEAN3_10680
HGSC *S. purpuratus* GLEAN3	GLEAN3_10693
HGSC *S. purpuratus* GLEAN3	GLEAN3_10695
HGSC *S. purpuratus* GLEAN3	GLEAN3_10940
HGSC *S. purpuratus* GLEAN3	GLEAN3_11042
HGSC *S. purpuratus* GLEAN3	GLEAN3_11328
HGSC *S. purpuratus* GLEAN3	GLEAN3_11454
HGSC *S. purpuratus* GLEAN3	GLEAN3_11481
HGSC *S. purpuratus* GLEAN3	GLEAN3_11536
HGSC *S. purpuratus* GLEAN3	GLEAN3_11537
HGSC *S. purpuratus* GLEAN3	GLEAN3_11539
HGSC *S. purpuratus* GLEAN3	GLEAN3_11540
HGSC *S. purpuratus* GLEAN3	GLEAN3_11570
HGSC *S. purpuratus* GLEAN3	GLEAN3_11823
HGSC *S. purpuratus* GLEAN3	GLEAN3_11949
HGSC *S. purpuratus* GLEAN3	GLEAN3_12257
HGSC *S. purpuratus* GLEAN3	GLEAN3_12464
HGSC *S. purpuratus* GLEAN3	GLEAN3_13111
HGSC *S. purpuratus* GLEAN3	GLEAN3_13162
HGSC *S. purpuratus* GLEAN3	GLEAN3_13470
HGSC *S. purpuratus* GLEAN3	GLEAN3_13676
HGSC *S. purpuratus* GLEAN3	GLEAN3_13751
HGSC *S. purpuratus* GLEAN3	GLEAN3_13824

HGSC *S. purpuratus* GLEAN3	GLEAN3_14041
HGSC *S. purpuratus* GLEAN3	GLEAN3_14191
HGSC *S. purpuratus* GLEAN3	GLEAN3_14266
HGSC *S. purpuratus* GLEAN3	GLEAN3_14352
HGSC *S. purpuratus* GLEAN3	GLEAN3_14926
HGSC *S. purpuratus* GLEAN3	GLEAN3_15029
HGSC *S. purpuratus* GLEAN3	GLEAN3_15066
HGSC *S. purpuratus* GLEAN3	GLEAN3_15303
HGSC *S. purpuratus* GLEAN3	GLEAN3_15533
HGSC *S. purpuratus* GLEAN3	GLEAN3_16388
HGSC *S. purpuratus* GLEAN3	GLEAN3_16438
HGSC *S. purpuratus* GLEAN3	GLEAN3_16457
HGSC *S. purpuratus* GLEAN3	GLEAN3_16501
HGSC *S. purpuratus* GLEAN3	GLEAN3_16536
HGSC *S. purpuratus* GLEAN3	GLEAN3_16554
HGSC *S. purpuratus* GLEAN3	GLEAN3_17104
HGSC *S. purpuratus* GLEAN3	GLEAN3_17180
HGSC *S. purpuratus* GLEAN3	GLEAN3_17529
HGSC *S. purpuratus* GLEAN3	GLEAN3_17530
HGSC *S. purpuratus* GLEAN3	GLEAN3_17735
HGSC *S. purpuratus* GLEAN3	GLEAN3_17794
HGSC *S. purpuratus* GLEAN3	GLEAN3_18055
HGSC *S. purpuratus* GLEAN3	GLEAN3_18100
HGSC *S. purpuratus* GLEAN3	GLEAN3_18211
HGSC *S. purpuratus* GLEAN3	GLEAN3_18212
HGSC *S. purpuratus* GLEAN3	GLEAN3_18380
HGSC *S. purpuratus* GLEAN3	GLEAN3_18409
HGSC *S. purpuratus* GLEAN3	GLEAN3_18410
HGSC *S. purpuratus* GLEAN3	GLEAN3_18519
HGSC *S. purpuratus* GLEAN3	GLEAN3_18838
HGSC *S. purpuratus* GLEAN3	GLEAN3_19309
HGSC *S. purpuratus* GLEAN3	GLEAN3_19661
HGSC *S. purpuratus* GLEAN3	GLEAN3_19834
HGSC *S. purpuratus* GLEAN3	GLEAN3_20258
HGSC *S. purpuratus* GLEAN3	GLEAN3_20259
HGSC *S. purpuratus* GLEAN3	GLEAN3_20428
HGSC *S. purpuratus* GLEAN3	GLEAN3_20472
HGSC *S. purpuratus* GLEAN3	GLEAN3_20654
HGSC *S. purpuratus* GLEAN3	GLEAN3_20996
HGSC *S. purpuratus* GLEAN3	GLEAN3_20997
HGSC *S. purpuratus* GLEAN3	GLEAN3_21075
HGSC *S. purpuratus* GLEAN3	GLEAN3_21162
HGSC *S. purpuratus* GLEAN3	GLEAN3_21225

HGSC *S. purpuratus* GLEAN3	GLEAN3_21395
HGSC *S. purpuratus* GLEAN3	GLEAN3_21415
HGSC *S. purpuratus* GLEAN3	GLEAN3_21420
HGSC *S. purpuratus* GLEAN3	GLEAN3_21502
HGSC *S. purpuratus* GLEAN3	GLEAN3_21787
HGSC *S. purpuratus* GLEAN3	GLEAN3_21908
HGSC *S. purpuratus* GLEAN3	GLEAN3_21936
HGSC *S. purpuratus* GLEAN3	GLEAN3_22707
HGSC *S. purpuratus* GLEAN3	GLEAN3_22708
HGSC *S. purpuratus* GLEAN3	GLEAN3_22909
HGSC *S. purpuratus* GLEAN3	GLEAN3_22911
HGSC *S. purpuratus* GLEAN3	GLEAN3_23033
HGSC *S. purpuratus* GLEAN3	GLEAN3_23035
HGSC *S. purpuratus* GLEAN3	GLEAN3_23321
HGSC *S. purpuratus* GLEAN3	GLEAN3_23491
HGSC *S. purpuratus* GLEAN3	GLEAN3_24062
HGSC *S. purpuratus* GLEAN3	GLEAN3_24204
HGSC *S. purpuratus* GLEAN3	GLEAN3_24205
HGSC *S. purpuratus* GLEAN3	GLEAN3_24207
HGSC *S. purpuratus* GLEAN3	GLEAN3_24385
HGSC *S. purpuratus* GLEAN3	GLEAN3_24386
HGSC *S. purpuratus* GLEAN3	GLEAN3_24404
HGSC *S. purpuratus* GLEAN3	GLEAN3_24429
HGSC *S. purpuratus* GLEAN3	GLEAN3_24479
HGSC *S. purpuratus* GLEAN3	GLEAN3_24501
HGSC *S. purpuratus* GLEAN3	GLEAN3_24590
HGSC *S. purpuratus* GLEAN3	GLEAN3_24731
HGSC *S. purpuratus* GLEAN3	GLEAN3_24733
HGSC *S. purpuratus* GLEAN3	GLEAN3_24815
HGSC *S. purpuratus* GLEAN3	GLEAN3_24847
HGSC *S. purpuratus* GLEAN3	GLEAN3_24868
HGSC *S. purpuratus* GLEAN3	GLEAN3_24960
HGSC *S. purpuratus* GLEAN3	GLEAN3_25076
HGSC *S. purpuratus* GLEAN3	GLEAN3_25136
HGSC *S. purpuratus* GLEAN3	GLEAN3_25719
HGSC *S. purpuratus* GLEAN3	GLEAN3_26200
HGSC *S. purpuratus* GLEAN3	GLEAN3_26274
HGSC *S. purpuratus* GLEAN3	GLEAN3_27162
HGSC *S. purpuratus* GLEAN3	GLEAN3_27164
HGSC *S. purpuratus* GLEAN3	GLEAN3_27222
HGSC *S. purpuratus* GLEAN3	GLEAN3_27735
HGSC *S. purpuratus* GLEAN3	GLEAN3_27798
HGSC *S. purpuratus* GLEAN3	GLEAN3_27815

	HGSC *S. purpuratus* GLEAN3	GLEAN3_28404
	HGSC *S. purpuratus* GLEAN3	GLEAN3_28576
	HGSC *S. purpuratus* GLEAN3	GLEAN3_28639
	HGSC *S. purpuratus* GLEAN3	GLEAN3_28893
D. melanogaster (fruit fly)	Ensembl release 38 'pep.abinitio'	GENSCAN00000000961
	Ensembl release 38 'pep.abinitio'	GENSCAN00000001346
	Ensembl release 38 'pep.abinitio'	GENSCAN00000005967
	Ensembl release 38 'pep.abinitio'	GENSCAN00000006330
	Ensembl release 38 'pep.abinitio'	GENSCAN00000006474
	Ensembl release 38 'pep.abinitio'	GENSCAN00000008355
	Ensembl release 38 'pep.abinitio'	GENSCAN00000011490
	Ensembl release 38 'pep.abinitio'	GENSCAN00000012092
	Ensembl release 38 'pep.abinitio'	GENSCAN00000015188
	Ensembl release 38 'pep.abinitio'	GENSCAN00000011840
	Ensembl release 38 'pep.abinitio'	GENSCAN00000012701
D. melanogaster (fruit fly)	Ensembl release 38 'pep'	CG1149-PA
	Ensembl release 38 'pep'	CG18241-PA
	Ensembl release 38 'pep'	CG2078-PA
	Ensembl release 38 'pep'	CG5490-PB
	Ensembl release 38 'pep'	CG5528-PA
	Ensembl release 38 'pep'	CG6890-PA
	Ensembl release 38 'pep'	CG7121-PA
	Ensembl release 38 'pep'	CG7250-PA
	Ensembl release 38 'pep'	CG7915-PB
	Ensembl release 38 'pep'	CG8595-PA
	Ensembl release 38 'pep'	CG8896-PA
D. melanogaster (fruit fly)	NCBI	GI:34334903
	NCBI	GI:17136634
	NCBI	GI:9246971
	NCBI	GI:9246969
	NCBI	GI:9246967
	NCBI	GI:17648021
	NCBI	GI:9246963
	NCBI	GI:9246961
	NCBI	GI:21356407
	NCBI	GI:19921906
	NCBI	GI:67472684
C. elegans	Ensembl release 38 'pep.abinitio'	GENEFINDER00000036667
	Ensembl release 38 'pep.abinitio'	GENEFINDER00000017849
C. elegans	Ensembl release 38 'pep'	C07F11.1
	Ensembl release 38 'pep'	F13B10.1d.1
C. elegans	NCBI	GI:71980576

	NCBI	GI:17553054
N. vectensis (sea anemone)	JGI *N. vectensis* v1.0 annotation	e_gw.141.61.1
	JGI *N. vectensis* v1.0 annotation	e_gw.26.221.1
	JGI *N. vectensis* v1.0 annotation	e_gw.5.187.1
	JGI *N. vectensis* v1.0 annotation	fgenesh1_pg.scaffold_3000058
	JGI *N. vectensis* v1.0 annotation	fgenesh1_pg.scaffold_47000068
	JGI *N. vectensis* v1.0 annotation	gw.141.125.1
	JGI *N. vectensis* v1.0 annotation	gw.152.108.1
N. vectensis (sea anemone)	JGI N. vectensis v1.0 assembly (genscan result)	scaffold_141_genscan_43
	JGI N. vectensis v1.0 assembly (genscan result)	scaffold_141_genscan_51
	JGI N. vectensis v1.0 assembly (genscan result)	scaffold_26_genscan_162
	JGI N. vectensis v1.0 assembly (genscan result)	scaffold_3_genscan_51
	JGI N. vectensis v1.0 assembly (genscan result)	scaffold_47_genscan_81
	JGI N. vectensis v1.0 assembly (genscan result)	scaffold_5_genscan_179

To get a more detailed view of TIR domain containing sequences in the selected species, different protein databases were checked and all sequences that could be confirmed by Pfam Protein Search or NCBI Conserved Domain Search under the default threshold were listed above.
