# Supplementary material for: Novel genes dramatically alter regulatory network topology in amphioxus
Source: Genome Biol. 2008 Aug 4;9(8):R123. doi: 10.1186/gb-2008-9-8-r123 (PMC2575513; doi:10.1186/gb-2008-9-8-r123)
Supplement: Additional data file 2 — NACHT domain containing sequences in different genomes. [file gb-2008-9-8-r123-S2.pdf]

Additional Table 2. List of NACHT domain containing sequences in different genomes

Genome	Data Source	Protein Identifier
H. sapiens (human)	Ensembl release 38 'pep.abinitio'	GENSCAN00000000608
	Ensembl release 38 'pep.abinitio'	GENSCAN00000000910
	Ensembl release 38 'pep.abinitio'	GENSCAN00000001215
	Ensembl release 38 'pep.abinitio'	GENSCAN00000001216
	Ensembl release 38 'pep.abinitio'	GENSCAN00000001217
	Ensembl release 38 'pep.abinitio'	GENSCAN00000004427
	Ensembl release 38 'pep.abinitio'	GENSCAN00000008263
	Ensembl release 38 'pep.abinitio'	GENSCAN00000015265
	Ensembl release 38 'pep.abinitio'	GENSCAN00000020422
	Ensembl release 38 'pep.abinitio'	GENSCAN00000022439
	Ensembl release 38 'pep.abinitio'	GENSCAN00000027967
	Ensembl release 38 'pep.abinitio'	GENSCAN00000028541
	Ensembl release 38 'pep.abinitio'	GENSCAN00000028634
	Ensembl release 38 'pep.abinitio'	GENSCAN00000040206
	Ensembl release 38 'pep.abinitio'	GENSCAN00000045567
	Ensembl release 38 'pep.abinitio'	GENSCAN00000054018
	Ensembl release 38 'pep.abinitio'	GENSCAN00000055729
	Ensembl release 38 'pep.abinitio'	GENSCAN00000056262
	Ensembl release 38 'pep.abinitio'	GENSCAN00000059219
	Ensembl release 38 'pep.abinitio'	GENSCAN00000059321
	Ensembl release 38 'pep.abinitio'	GENSCAN00000066521
	Ensembl release 38 'pep.abinitio'	GENSCAN00000066524
H. sapiens (human)	Ensembl release 38 'pep'	ENSP00000222823
	Ensembl release 38 'pep'	ENSP00000323897
	Ensembl release 38 'pep'	ENSP00000300589
	Ensembl release 38 'pep'	ENSP00000327462
	Ensembl release 38 'pep'	ENSP00000346231
	Ensembl release 38 'pep'	ENSP00000291971
	Ensembl release 38 'pep'	ENSP00000339491
	Ensembl release 38 'pep'	ENSP00000344074
	Ensembl release 38 'pep'	ENSP00000353251
	Ensembl release 38 'pep'	ENSP00000344787
	Ensembl release 38 'pep'	ENSP00000343891
	Ensembl release 38 'pep'	ENSP00000262510
	Ensembl release 38 'pep'	ENSP00000311233
	Ensembl release 38 'pep'	ENSP00000194097
	Ensembl release 38 'pep'	ENSP00000327763
	Ensembl release 38 'pep'	ENSP00000324366
	Ensembl release 38 'pep'	ENSP00000299481

	Ensembl release 38 'pep'	ENSP00000355449
	Ensembl release 38 'pep'	ENSP00000316328
	Ensembl release 38 'pep'	ENSP00000309767
	Ensembl release 38 'pep'	ENSP00000321876
	Ensembl release 38 'pep'	ENSP00000354159
	Ensembl release 38 'pep'	ENSP00000292199
H. sapiens (human)	NCBI	GI:14719829
	NCBI	GI:8923473
	NCBI	GI:34878693
	NCBI	GI:19745162
	NCBI	GI:32481211
	NCBI	GI:21264320
	NCBI	GI:45593128
	NCBI	GI:33667040
	NCBI	GI:33519450
	NCBI	GI:28827807
	NCBI	GI:21450725
	NCBI	GI:21955154
	NCBI	GI:28827789
	NCBI	GI:28827813
	NCBI	GI:5174617
	NCBI	GI:11545912
	NCBI	GI:40788015
	NCBI	GI:4557749
	NCBI	GI:4758752
	NCBI	GI:30524928
	NCBI	GI:25777608
	NCBI	GI:28951071
M. musculus (mouse)	Ensembl release 38 'pep.abinitio'	GENSCAN00000154468
	Ensembl release 38 'pep.abinitio'	GENSCAN00000156858
	Ensembl release 38 'pep.abinitio'	GENSCAN00000160146
	Ensembl release 38 'pep.abinitio'	GENSCAN00000160147
	Ensembl release 38 'pep.abinitio'	GENSCAN00000161183
	Ensembl release 38 'pep.abinitio'	GENSCAN00000165190
	Ensembl release 38 'pep.abinitio'	GENSCAN00000165211
	Ensembl release 38 'pep.abinitio'	GENSCAN00000165287
	Ensembl release 38 'pep.abinitio'	GENSCAN00000168608
	Ensembl release 38 'pep.abinitio'	GENSCAN00000168611
	Ensembl release 38 'pep.abinitio'	GENSCAN00000168620
	Ensembl release 38 'pep.abinitio'	GENSCAN00000168988
	Ensembl release 38 'pep.abinitio'	GENSCAN00000170402
	Ensembl release 38 'pep.abinitio'	GENSCAN00000171143

	Ensembl release 38 'pep.abinitio'	GENSCAN00000180938
	Ensembl release 38 'pep.abinitio'	GENSCAN00000180939
	Ensembl release 38 'pep.abinitio'	GENSCAN00000183740
	Ensembl release 38 'pep.abinitio'	GENSCAN00000184049
	Ensembl release 38 'pep.abinitio'	GENSCAN00000185655
	Ensembl release 38 'pep.abinitio'	GENSCAN00000185657
	Ensembl release 38 'pep.abinitio'	GENSCAN00000189363
	Ensembl release 38 'pep.abinitio'	GENSCAN00000197447
	Ensembl release 38 'pep.abinitio'	GENSCAN00000198101
	Ensembl release 38 'pep.abinitio'	GENSCAN00000213501
	Ensembl release 38 'pep.abinitio'	GENSCAN00000214335
	Ensembl release 38 'pep.abinitio'	GENSCAN00000216425
	Ensembl release 38 'pep.abinitio'	GENSCAN00000217705
	Ensembl release 38 'pep.abinitio'	GENSCAN00000220435
	Ensembl release 38 'pep.abinitio'	GENSCAN00000220778
	Ensembl release 38 'pep.abinitio'	GENSCAN00000222274
	Ensembl release 38 'pep.abinitio'	GENSCAN00000224066
	Ensembl release 38 'pep.abinitio'	GENSCAN00000224634
	Ensembl release 38 'pep.abinitio'	GENSCAN00000225683
M. musculus (mouse)	Ensembl release 38 'pep'	ENSMUSP00000015866
	Ensembl release 38 'pep'	ENSMUSP00000022142
	Ensembl release 38 'pep'	ENSMUSP00000022144
	Ensembl release 38 'pep'	ENSMUSP00000023147
	Ensembl release 38 'pep'	ENSMUSP00000038186
	Ensembl release 38 'pep'	ENSMUSP00000041766
	Ensembl release 38 'pep'	ENSMUSP00000042759
	Ensembl release 38 'pep'	ENSMUSP00000043881
	Ensembl release 38 'pep'	ENSMUSP00000045077
	Ensembl release 38 'pep'	ENSMUSP00000046503
	Ensembl release 38 'pep'	ENSMUSP00000050252
	Ensembl release 38 'pep'	ENSMUSP00000050538
	Ensembl release 38 'pep'	ENSMUSP00000055747
	Ensembl release 38 'pep'	ENSMUSP00000058611
	Ensembl release 38 'pep'	ENSMUSP00000059637
	Ensembl release 38 'pep'	ENSMUSP00000060688
	Ensembl release 38 'pep'	ENSMUSP00000063752
	Ensembl release 38 'pep'	ENSMUSP00000065508
	Ensembl release 38 'pep'	ENSMUSP00000066841
	Ensembl release 38 'pep'	ENSMUSP00000069511
	Ensembl release 38 'pep'	ENSMUSP00000071685
	Ensembl release 38 'pep'	ENSMUSP00000072895
	Ensembl release 38 'pep'	ENSMUSP00000075794
	Ensembl release 38 'pep'	ENSMUSP00000077561

	Ensembl release 38 'pep'	ENSMUSP00000078440
	Ensembl release 38 'pep'	ENSMUSP00000079455
	Ensembl release 38 'pep'	ENSMUSP00000080222
	Ensembl release 38 'pep'	ENSMUSP00000081819
	Ensembl release 38 'pep'	ENSMUSP00000083106
	Ensembl release 38 'pep'	ENSMUSP00000083143
	Ensembl release 38 'pep'	ENSMUSP00000083450
	Ensembl release 38 'pep'	ENSMUSP00000091588
	Ensembl release 38 'pep'	ENSMUSP00000094030
C. familiaris (dog)	Ensembl release 38 'pep.abinitio'	GENSCAN00000000648
	Ensembl release 38 'pep.abinitio'	GENSCAN00000000856
	Ensembl release 38 'pep.abinitio'	GENSCAN00000004214
	Ensembl release 38 'pep.abinitio'	GENSCAN00000008582
	Ensembl release 38 'pep.abinitio'	GENSCAN00000016719
	Ensembl release 38 'pep.abinitio'	GENSCAN00000019061
	Ensembl release 38 'pep.abinitio'	GENSCAN00000035249
	Ensembl release 38 'pep.abinitio'	GENSCAN00000036025
	Ensembl release 38 'pep.abinitio'	GENSCAN00000037452
	Ensembl release 38 'pep.abinitio'	GENSCAN00000041479
	Ensembl release 38 'pep.abinitio'	GENSCAN00000043070
	Ensembl release 38 'pep.abinitio'	GENSCAN00000044030
	Ensembl release 38 'pep.abinitio'	GENSCAN00000056756
	Ensembl release 38 'pep.abinitio'	GENSCAN00000057986
	Ensembl release 38 'pep.abinitio'	GENSCAN00000064727
	Ensembl release 38 'pep.abinitio'	GENSCAN00000068599
	Ensembl release 38 'pep.abinitio'	GENSCAN00000077417
C. familiaris (dog)	Ensembl release 38 'pep'	ENSCAFP00000003638
	Ensembl release 38 'pep'	ENSCAFP00000003649
	Ensembl release 38 'pep'	ENSCAFP00000003659
	Ensembl release 38 'pep'	ENSCAFP00000003675
	Ensembl release 38 'pep'	ENSCAFP00000003834
	Ensembl release 38 'pep'	ENSCAFP00000003994
	Ensembl release 38 'pep'	ENSCAFP00000004572
	Ensembl release 38 'pep'	ENSCAFP00000009686
	Ensembl release 38 'pep'	ENSCAFP00000010005
	Ensembl release 38 'pep'	ENSCAFP00000010243
	Ensembl release 38 'pep'	ENSCAFP00000013161
	Ensembl release 38 'pep'	ENSCAFP00000014425
	Ensembl release 38 'pep'	ENSCAFP00000015745
	Ensembl release 38 'pep'	ENSCAFP00000017960
	Ensembl release 38 'pep'	ENSCAFP00000022816
	Ensembl release 38 'pep'	ENSCAFP00000026586

	Ensembl release 38 'pep'	ENSCAFP00000027946
	Ensembl release 38 'pep'	ENSCAFP00000028434
G. gallus (chicken)	Ensembl release 38 'pep.abinitio'	GENSCAN00000028018
	Ensembl release 38 'pep.abinitio'	GENSCAN00000043077
	Ensembl release 38 'pep.abinitio'	GENSCAN00000043886
	Ensembl release 38 'pep.abinitio'	GENSCAN00000058421
	Ensembl release 38 'pep.abinitio'	GENSCAN00000059775
	Ensembl release 38 'pep.abinitio'	GENSCAN00000070918
G. gallus (chicken)	Ensembl release 38 'pep'	ENSGALP00000001906
	Ensembl release 38 'pep'	ENSGALP00000008260
	Ensembl release 38 'pep'	ENSGALP00000010953
	Ensembl release 38 'pep'	ENSGALP00000011599
	Ensembl release 38 'pep'	ENSGALP00000012708
	Ensembl release 38 'pep'	ENSGALP00000018785
X. tropicalis (western clawed frog)	Ensembl release 38 'pep.abinitio'	GENSCAN00000004010
	Ensembl release 38 'pep.abinitio'	GENSCAN00000013743
	Ensembl release 38 'pep.abinitio'	GENSCAN00000015124
	Ensembl release 38 'pep.abinitio'	GENSCAN00000016256
	Ensembl release 38 'pep.abinitio'	GENSCAN00000017302
	Ensembl release 38 'pep.abinitio'	GENSCAN00000026853
	Ensembl release 38 'pep.abinitio'	GENSCAN00000026863
	Ensembl release 38 'pep.abinitio'	GENSCAN00000026868
	Ensembl release 38 'pep.abinitio'	GENSCAN00000026883
	Ensembl release 38 'pep.abinitio'	GENSCAN00000030400
	Ensembl release 38 'pep.abinitio'	GENSCAN00000032287
	Ensembl release 38 'pep.abinitio'	GENSCAN00000035595
	Ensembl release 38 'pep.abinitio'	GENSCAN00000040978
	Ensembl release 38 'pep.abinitio'	GENSCAN00000047097
	Ensembl release 38 'pep.abinitio'	GENSCAN00000049710
	Ensembl release 38 'pep.abinitio'	GENSCAN00000069736
	Ensembl release 38 'pep.abinitio'	GENSCAN00000069752
	Ensembl release 38 'pep.abinitio'	GENSCAN00000070753
	Ensembl release 38 'pep.abinitio'	GENSCAN00000070784
	Ensembl release 38 'pep.abinitio'	GENSCAN00000070793
	Ensembl release 38 'pep.abinitio'	GENSCAN00000070809
X. tropicalis (western clawed frog)	Ensembl release 38 'pep'	ENSXETP00000009512
	Ensembl release 38 'pep'	ENSXETP00000019175
	Ensembl release 38 'pep'	ENSXETP00000019188
	Ensembl release 38 'pep'	ENSXETP00000019215
	Ensembl release 38 'pep'	ENSXETP00000019240
	Ensembl release 38 'pep'	ENSXETP00000030524
	Ensembl release 38 'pep'	ENSXETP00000040025

	Ensembl release 38 'pep'	ENSXETP00000046634
	Ensembl release 38 'pep'	ENSXETP00000047642
	Ensembl release 38 'pep'	ENSXETP00000054623
D. rerio (zebrafish)	Ensembl release 38 'pep.abinitio'	GENSCAN00000002116
	Ensembl release 38 'pep.abinitio'	GENSCAN00000002446
	Ensembl release 38 'pep.abinitio'	GENSCAN00000004164
	Ensembl release 38 'pep.abinitio'	GENSCAN00000012757
	Ensembl release 38 'pep.abinitio'	GENSCAN00000013244
	Ensembl release 38 'pep.abinitio'	GENSCAN00000013254
	Ensembl release 38 'pep.abinitio'	GENSCAN00000017201
	Ensembl release 38 'pep.abinitio'	GENSCAN00000018328
	Ensembl release 38 'pep.abinitio'	GENSCAN00000025665
	Ensembl release 38 'pep.abinitio'	GENSCAN00000025668
	Ensembl release 38 'pep.abinitio'	GENSCAN00000026656
	Ensembl release 38 'pep.abinitio'	GENSCAN00000031000
	Ensembl release 38 'pep.abinitio'	GENSCAN00000031330
	Ensembl release 38 'pep.abinitio'	GENSCAN00000032635
	Ensembl release 38 'pep.abinitio'	GENSCAN00000032637
	Ensembl release 38 'pep.abinitio'	GENSCAN00000037878
	Ensembl release 38 'pep.abinitio'	GENSCAN00000038851
	Ensembl release 38 'pep.abinitio'	GENSCAN00000042051
	Ensembl release 38 'pep.abinitio'	GENSCAN00000044593
D. rerio (zebrafish)	Ensembl release 38 'pep'	ENSDARP00000013454
	Ensembl release 38 'pep'	ENSDARP00000015139
	Ensembl release 38 'pep'	ENSDARP00000035227
	Ensembl release 38 'pep'	ENSDARP00000052744
	Ensembl release 38 'pep'	ENSDARP00000056172
	Ensembl release 38 'pep'	ENSDARP00000056257
	Ensembl release 38 'pep'	ENSDARP00000056299
	Ensembl release 38 'pep'	ENSDARP00000056352
	Ensembl release 38 'pep'	ENSDARP00000061179
	Ensembl release 38 'pep'	ENSDARP00000065811
F. rubripes (Japanese pufferfish)	Ensembl release 38 'pep.abinitio'	GENSCANSLICE00000000062
	Ensembl release 38 'pep.abinitio'	GENSCANSLICE00000000378
	Ensembl release 38 'pep.abinitio'	GENSCANSLICE00000000479
	Ensembl release 38 'pep.abinitio'	GENSCANSLICE00000000561
	Ensembl release 38 'pep.abinitio'	GENSCANSLICE00000001128
	Ensembl release 38 'pep.abinitio'	GENSCANSLICE00000001347
	Ensembl release 38 'pep.abinitio'	GENSCANSLICE00000002016
	Ensembl release 38 'pep.abinitio'	GENSCANSLICE00000002747
	Ensembl release 38 'pep.abinitio'	GENSCANSLICE00000002988
	Ensembl release 38 'pep.abinitio'	GENSCANSLICE00000003023

Ensembl release 38 'pep.abinitio'	GENSCANSLICE00000003298
Ensembl release 38 'pep.abinitio'	GENSCANSLICE00000003348
Ensembl release 38 'pep.abinitio'	GENSCANSLICE00000003741
Ensembl release 38 'pep.abinitio'	GENSCANSLICE00000003748
Ensembl release 38 'pep.abinitio'	GENSCANSLICE00000003763
Ensembl release 38 'pep.abinitio'	GENSCANSLICE00000003765
Ensembl release 38 'pep.abinitio'	GENSCANSLICE00000003900
Ensembl release 38 'pep.abinitio'	GENSCANSLICE00000003907
Ensembl release 38 'pep.abinitio'	GENSCANSLICE00000003955
Ensembl release 38 'pep.abinitio'	GENSCANSLICE00000004029
Ensembl release 38 'pep.abinitio'	GENSCANSLICE00000004479
Ensembl release 38 'pep.abinitio'	GENSCANSLICE00000004624
Ensembl release 38 'pep.abinitio'	GENSCANSLICE00000004706
Ensembl release 38 'pep.abinitio'	GENSCANSLICE00000005037
Ensembl release 38 'pep.abinitio'	GENSCANSLICE00000005498
Ensembl release 38 'pep.abinitio'	GENSCANSLICE00000005670
Ensembl release 38 'pep.abinitio'	GENSCANSLICE00000005904
Ensembl release 38 'pep.abinitio'	GENSCANSLICE00000006151
Ensembl release 38 'pep.abinitio'	GENSCANSLICE00000006286
Ensembl release 38 'pep.abinitio'	GENSCANSLICE00000006462
Ensembl release 38 'pep.abinitio'	GENSCANSLICE00000006813
Ensembl release 38 'pep.abinitio'	GENSCANSLICE00000007471
Ensembl release 38 'pep.abinitio'	GENSCANSLICE00000007657
Ensembl release 38 'pep.abinitio'	GENSCANSLICE00000007821
Ensembl release 38 'pep.abinitio'	GENSCANSLICE00000008271
Ensembl release 38 'pep.abinitio'	GENSCANSLICE00000008401
Ensembl release 38 'pep.abinitio'	GENSCANSLICE00000009090
Ensembl release 38 'pep.abinitio'	GENSCANSLICE00000009451
Ensembl release 38 'pep.abinitio'	GENSCANSLICE00000010357
Ensembl release 38 'pep.abinitio'	GENSCANSLICE00000010626
Ensembl release 38 'pep.abinitio'	GENSCANSLICE00000010720
Ensembl release 38 'pep.abinitio'	GENSCANSLICE00000010721
Ensembl release 38 'pep.abinitio'	GENSCANSLICE00000010842
Ensembl release 38 'pep.abinitio'	GENSCANSLICE00000010980
Ensembl release 38 'pep.abinitio'	GENSCANSLICE00000011252
Ensembl release 38 'pep.abinitio'	GENSCANSLICE00000011312
Ensembl release 38 'pep.abinitio'	GENSCANSLICE00000011756
Ensembl release 38 'pep.abinitio'	GENSCANSLICE00000012164
Ensembl release 38 'pep.abinitio'	GENSCANSLICE00000012167
Ensembl release 38 'pep.abinitio'	GENSCANSLICE00000012711
Ensembl release 38 'pep.abinitio'	GENSCANSLICE00000012824
Ensembl release 38 'pep.abinitio'	GENSCANSLICE00000012992
Ensembl release 38 'pep.abinitio'	GENSCANSLICE00000013036

Ensembl release 38 'pep.abinitio'	GENSCANSLICE00000013068
Ensembl release 38 'pep.abinitio'	GENSCANSLICE00000013146
Ensembl release 38 'pep.abinitio'	GENSCANSLICE00000013669
Ensembl release 38 'pep.abinitio'	GENSCANSLICE00000013783
Ensembl release 38 'pep.abinitio'	GENSCANSLICE00000014693
Ensembl release 38 'pep.abinitio'	GENSCANSLICE00000015367
Ensembl release 38 'pep.abinitio'	GENSCANSLICE00000015400
Ensembl release 38 'pep.abinitio'	GENSCANSLICE00000015449
Ensembl release 38 'pep.abinitio'	GENSCANSLICE00000015901
Ensembl release 38 'pep.abinitio'	GENSCANSLICE00000015922
Ensembl release 38 'pep.abinitio'	GENSCANSLICE00000015945
Ensembl release 38 'pep.abinitio'	GENSCANSLICE00000016037
Ensembl release 38 'pep.abinitio'	GENSCANSLICE00000016062
Ensembl release 38 'pep.abinitio'	GENSCANSLICE00000016103
Ensembl release 38 'pep.abinitio'	GENSCANSLICE00000016427
Ensembl release 38 'pep.abinitio'	GENSCANSLICE00000016569
Ensembl release 38 'pep.abinitio'	GENSCANSLICE00000016678
Ensembl release 38 'pep.abinitio'	GENSCANSLICE00000016693
Ensembl release 38 'pep.abinitio'	GENSCANSLICE00000016974
Ensembl release 38 'pep.abinitio'	GENSCANSLICE00000017428
Ensembl release 38 'pep.abinitio'	GENSCANSLICE00000017475
Ensembl release 38 'pep.abinitio'	GENSCANSLICE00000017698
Ensembl release 38 'pep.abinitio'	GENSCANSLICE00000018105
Ensembl release 38 'pep.abinitio'	GENSCANSLICE00000018248
Ensembl release 38 'pep.abinitio'	GENSCANSLICE00000019003
Ensembl release 38 'pep.abinitio'	GENSCANSLICE00000019210
Ensembl release 38 'pep.abinitio'	GENSCANSLICE00000019312
Ensembl release 38 'pep.abinitio'	GENSCANSLICE00000019448
Ensembl release 38 'pep.abinitio'	GENSCANSLICE00000019803
Ensembl release 38 'pep.abinitio'	GENSCANSLICE00000019921
Ensembl release 38 'pep.abinitio'	GENSCANSLICE00000020017
Ensembl release 38 'pep.abinitio'	GENSCANSLICE00000020174
Ensembl release 38 'pep.abinitio'	GENSCANSLICE00000020748
Ensembl release 38 'pep.abinitio'	GENSCANSLICE00000020805
Ensembl release 38 'pep.abinitio'	GENSCANSLICE00000021001
Ensembl release 38 'pep.abinitio'	GENSCANSLICE00000021249
Ensembl release 38 'pep.abinitio'	GENSCANSLICE00000021433
Ensembl release 38 'pep.abinitio'	GENSCANSLICE00000021810
Ensembl release 38 'pep.abinitio'	GENSCANSLICE00000021825
Ensembl release 38 'pep.abinitio'	GENSCANSLICE00000021896
Ensembl release 38 'pep.abinitio'	GENSCANSLICE00000022639
Ensembl release 38 'pep.abinitio'	GENSCANSLICE00000023026
Ensembl release 38 'pep.abinitio'	GENSCANSLICE00000023370

	Ensembl release 38 'pep.abinitio'	GENSCANSLICE00000023709
	Ensembl release 38 'pep.abinitio'	GENSCANSLICE00000024497
	Ensembl release 38 'pep.abinitio'	GENSCANSLICE00000024515
	Ensembl release 38 'pep.abinitio'	GENSCANSLICE00000024776
	Ensembl release 38 'pep.abinitio'	GENSCANSLICE00000024783
	Ensembl release 38 'pep.abinitio'	GENSCANSLICE00000024880
	Ensembl release 38 'pep.abinitio'	GENSCANSLICE00000025205
	Ensembl release 38 'pep.abinitio'	GENSCANSLICE00000025220
	Ensembl release 38 'pep.abinitio'	GENSCANSLICE00000025373
	Ensembl release 38 'pep.abinitio'	GENSCANSLICE00000025507
	Ensembl release 38 'pep.abinitio'	GENSCANSLICE00000025734
	Ensembl release 38 'pep.abinitio'	GENSCANSLICE00000025871
	Ensembl release 38 'pep.abinitio'	GENSCANSLICE00000027051
	Ensembl release 38 'pep.abinitio'	GENSCANSLICE00000027337
	Ensembl release 38 'pep.abinitio'	GENSCANSLICE00000027395
	Ensembl release 38 'pep.abinitio'	GENSCANSLICE00000027468
	Ensembl release 38 'pep.abinitio'	GENSCANSLICE00000027504
	Ensembl release 38 'pep.abinitio'	GENSCANSLICE00000027696
	Ensembl release 38 'pep.abinitio'	GENSCANSLICE00000027895
	Ensembl release 38 'pep.abinitio'	GENSCANSLICE00000028565
F. rubripes (Japanese pufferfish)	Ensembl release 38 'pep'	NEWSINFRUP00000127580
	Ensembl release 38 'pep'	NEWSINFRUP00000129039
	Ensembl release 38 'pep'	NEWSINFRUP00000130946
	Ensembl release 38 'pep'	NEWSINFRUP00000132575
	Ensembl release 38 'pep'	NEWSINFRUP00000133735
	Ensembl release 38 'pep'	NEWSINFRUP00000134480
	Ensembl release 38 'pep'	NEWSINFRUP00000135500
	Ensembl release 38 'pep'	NEWSINFRUP00000141434
	Ensembl release 38 'pep'	NEWSINFRUP00000142189
	Ensembl release 38 'pep'	NEWSINFRUP00000143465
	Ensembl release 38 'pep'	NEWSINFRUP00000146443
	Ensembl release 38 'pep'	NEWSINFRUP00000146538
	Ensembl release 38 'pep'	NEWSINFRUP00000153199
	Ensembl release 38 'pep'	NEWSINFRUP00000155380
	Ensembl release 38 'pep'	NEWSINFRUP00000155383
	Ensembl release 38 'pep'	NEWSINFRUP00000156887
	Ensembl release 38 'pep'	NEWSINFRUP00000157307
	Ensembl release 38 'pep'	NEWSINFRUP00000157696
	Ensembl release 38 'pep'	NEWSINFRUP00000158091
	Ensembl release 38 'pep'	NEWSINFRUP00000159769
	Ensembl release 38 'pep'	NEWSINFRUP00000159770
	Ensembl release 38 'pep'	NEWSINFRUP00000159961
	Ensembl release 38 'pep'	NEWSINFRUP00000170468

Ensembl release 38 'pep'	NEWSINFRUP00000175400
Ensembl release 38 'pep'	NEWSINFRUP00000177426
Ensembl release 38 'pep'	NEWSINFRUP00000177556
Ensembl release 38 'pep'	NEWSINFRUP00000177567
Ensembl release 38 'pep'	NEWSINFRUP00000177706
Ensembl release 38 'pep'	NEWSINFRUP00000177793
Ensembl release 38 'pep'	NEWSINFRUP00000177812
Ensembl release 38 'pep'	NEWSINFRUP00000177839
Ensembl release 38 'pep'	NEWSINFRUP00000177848
Ensembl release 38 'pep'	NEWSINFRUP00000178067
Ensembl release 38 'pep'	NEWSINFRUP00000178091
Ensembl release 38 'pep'	NEWSINFRUP00000178122
Ensembl release 38 'pep'	NEWSINFRUP00000178168
Ensembl release 38 'pep'	NEWSINFRUP00000178703
Ensembl release 38 'pep'	NEWSINFRUP00000178725
Ensembl release 38 'pep'	NEWSINFRUP00000178774
Ensembl release 38 'pep'	NEWSINFRUP00000178972
Ensembl release 38 'pep'	NEWSINFRUP00000179032
Ensembl release 38 'pep'	NEWSINFRUP00000179096
Ensembl release 38 'pep'	NEWSINFRUP00000179161
Ensembl release 38 'pep'	NEWSINFRUP00000179164
Ensembl release 38 'pep'	NEWSINFRUP00000179178
Ensembl release 38 'pep'	NEWSINFRUP00000179535
Ensembl release 38 'pep'	NEWSINFRUP00000179663
Ensembl release 38 'pep'	NEWSINFRUP00000179697
Ensembl release 38 'pep'	NEWSINFRUP00000179886
Ensembl release 38 'pep'	NEWSINFRUP00000179927
Ensembl release 38 'pep'	NEWSINFRUP00000180381
Ensembl release 38 'pep'	NEWSINFRUP00000180502
Ensembl release 38 'pep'	NEWSINFRUP00000180592
Ensembl release 38 'pep'	NEWSINFRUP00000180612
Ensembl release 38 'pep'	NEWSINFRUP00000180718
Ensembl release 38 'pep'	NEWSINFRUP00000180792
Ensembl release 38 'pep'	NEWSINFRUP00000180928
Ensembl release 38 'pep'	NEWSINFRUP00000181102
Ensembl release 38 'pep'	NEWSINFRUP00000181110
Ensembl release 38 'pep'	NEWSINFRUP00000181427
Ensembl release 38 'pep'	NEWSINFRUP00000181430
Ensembl release 38 'pep'	NEWSINFRUP00000181465
Ensembl release 38 'pep'	NEWSINFRUP00000181846
Ensembl release 38 'pep'	NEWSINFRUP00000181929
Ensembl release 38 'pep'	NEWSINFRUP00000181970
Ensembl release 38 'pep'	NEWSINFRUP00000182093

	Ensembl release 38 'pep'	NEWSINFRUP00000182112
	Ensembl release 38 'pep'	NEWSINFRUP00000182122
	Ensembl release 38 'pep'	NEWSINFRUP00000182203
	Ensembl release 38 'pep'	NEWSINFRUP00000182204
	Ensembl release 38 'pep'	NEWSINFRUP00000182280
	Ensembl release 38 'pep'	NEWSINFRUP00000182339
	Ensembl release 38 'pep'	NEWSINFRUP00000182422
	Ensembl release 38 'pep'	NEWSINFRUP00000182506
	Ensembl release 38 'pep'	NEWSINFRUP00000183002
	Ensembl release 38 'pep'	NEWSINFRUP00000183047
	Ensembl release 38 'pep'	NEWSINFRUP00000183080
T. nigroviridis (green pufferfish)	Ensembl release 38 'pep.abinitio'	GIDT00029328001
	Ensembl release 38 'pep.abinitio'	GSCT00002781001
	Ensembl release 38 'pep.abinitio'	GSCT00007551001
	Ensembl release 38 'pep.abinitio'	GSCT00014284001
	Ensembl release 38 'pep.abinitio'	GSCT00020363001
	Ensembl release 38 'pep.abinitio'	GSCT00020484001
	Ensembl release 38 'pep.abinitio'	GSCT00022202001
	Ensembl release 38 'pep.abinitio'	GSCT00023404001
	Ensembl release 38 'pep.abinitio'	GWSHT00007909001
	Ensembl release 38 'pep.abinitio'	GWSHT00009127001
	Ensembl release 38 'pep.abinitio'	GWSHT00009665001
T. nigroviridis (green pufferfish)	Ensembl release 38 'pep'	GSTENP00000269001
	Ensembl release 38 'pep'	GSTENP00000753001
	Ensembl release 38 'pep'	GSTENP00005101001
	Ensembl release 38 'pep'	GSTENP00005304001
	Ensembl release 38 'pep'	GSTENP00005633001
	Ensembl release 38 'pep'	GSTENP00010767001
	Ensembl release 38 'pep'	GSTENP00010923001
	Ensembl release 38 'pep'	GSTENP00012155001
	Ensembl release 38 'pep'	GSTENP00012203001
	Ensembl release 38 'pep'	GSTENP00021862001
	Ensembl release 38 'pep'	GSTENP00024060001
	Ensembl release 38 'pep'	GSTENP00024994001
	Ensembl release 38 'pep'	GSTENP00026128001
	Ensembl release 38 'pep'	GSTENP00033222001
	Ensembl release 38 'pep'	GSTENP00034770001
B. floridae (amphioxus)	JGI *B. floridae* v1.0 annotation	estExt_fgenesh2_pg.C_1870006
	JGI *B. floridae* v1.0 annotation	estExt_fgenesh2_pg.C_2190014
	JGI *B. floridae* v1.0 annotation	estExt_fgenesh2_pg.C_3710025
	JGI *B. floridae* v1.0 annotation	estExt_fgenesh2_pg.C_400088
	JGI *B. floridae* v1.0 annotation	estExt_fgenesh2_pg.C_4110028

JGI *B. floridae* v1.0 annotation	estExt_fgenesh2_pg.C_4900008
JGI *B. floridae* v1.0 annotation	estExt_fgenesh2_pg.C_620041
JGI *B. floridae* v1.0 annotation	estExt_fgenesh2_pg.C_90156
JGI *B. floridae* v1.0 annotation	fgenesh2_pg.scaffold_104000024
JGI *B. floridae* v1.0 annotation	fgenesh2_pg.scaffold_108000006
JGI *B. floridae* v1.0 annotation	fgenesh2_pg.scaffold_108000007
JGI *B. floridae* v1.0 annotation	fgenesh2_pg.scaffold_110000051
JGI *B. floridae* v1.0 annotation	fgenesh2_pg.scaffold_111000114
JGI *B. floridae* v1.0 annotation	fgenesh2_pg.scaffold_111000115
JGI *B. floridae* v1.0 annotation	fgenesh2_pg.scaffold_111000116
JGI *B. floridae* v1.0 annotation	fgenesh2_pg.scaffold_113000055
JGI *B. floridae* v1.0 annotation	fgenesh2_pg.scaffold_113000056
JGI *B. floridae* v1.0 annotation	fgenesh2_pg.scaffold_113000058
JGI *B. floridae* v1.0 annotation	fgenesh2_pg.scaffold_129000001
JGI *B. floridae* v1.0 annotation	fgenesh2_pg.scaffold_129000002
JGI *B. floridae* v1.0 annotation	fgenesh2_pg.scaffold_136000031
JGI *B. floridae* v1.0 annotation	fgenesh2_pg.scaffold_14000015
JGI *B. floridae* v1.0 annotation	fgenesh2_pg.scaffold_148000014
JGI *B. floridae* v1.0 annotation	fgenesh2_pg.scaffold_154000048
JGI *B. floridae* v1.0 annotation	fgenesh2_pg.scaffold_155000019
JGI *B. floridae* v1.0 annotation	fgenesh2_pg.scaffold_155000031
JGI *B. floridae* v1.0 annotation	fgenesh2_pg.scaffold_157000018
JGI *B. floridae* v1.0 annotation	fgenesh2_pg.scaffold_157000063
JGI *B. floridae* v1.0 annotation	fgenesh2_pg.scaffold_173000009
JGI *B. floridae* v1.0 annotation	fgenesh2_pg.scaffold_187000007
JGI *B. floridae* v1.0 annotation	fgenesh2_pg.scaffold_187000009
JGI *B. floridae* v1.0 annotation	fgenesh2_pg.scaffold_187000013
JGI *B. floridae* v1.0 annotation	fgenesh2_pg.scaffold_187000016
JGI *B. floridae* v1.0 annotation	fgenesh2_pg.scaffold_187000018
JGI *B. floridae* v1.0 annotation	fgenesh2_pg.scaffold_187000032
JGI *B. floridae* v1.0 annotation	fgenesh2_pg.scaffold_187000037
JGI *B. floridae* v1.0 annotation	fgenesh2_pg.scaffold_187000038
JGI *B. floridae* v1.0 annotation	fgenesh2_pg.scaffold_187000040
JGI *B. floridae* v1.0 annotation	fgenesh2_pg.scaffold_187000042
JGI *B. floridae* v1.0 annotation	fgenesh2_pg.scaffold_187000044
JGI *B. floridae* v1.0 annotation	fgenesh2_pg.scaffold_190000004
JGI *B. floridae* v1.0 annotation	fgenesh2_pg.scaffold_190000005
JGI *B. floridae* v1.0 annotation	fgenesh2_pg.scaffold_190000006
JGI *B. floridae* v1.0 annotation	fgenesh2_pg.scaffold_190000007
JGI *B. floridae* v1.0 annotation	fgenesh2_pg.scaffold_19000229
JGI *B. floridae* v1.0 annotation	fgenesh2_pg.scaffold_199000027
JGI *B. floridae* v1.0 annotation	fgenesh2_pg.scaffold_20000114
JGI *B. floridae* v1.0 annotation	fgenesh2_pg.scaffold_21000036

JGI *B. floridae* v1.0 annotation	fgenesh2_pg.scaffold_21000095
JGI *B. floridae* v1.0 annotation	fgenesh2_pg.scaffold_214000028
JGI *B. floridae* v1.0 annotation	fgenesh2_pg.scaffold_214000029
JGI *B. floridae* v1.0 annotation	fgenesh2_pg.scaffold_219000011
JGI *B. floridae* v1.0 annotation	fgenesh2_pg.scaffold_219000021
JGI *B. floridae* v1.0 annotation	fgenesh2_pg.scaffold_219000022
JGI *B. floridae* v1.0 annotation	fgenesh2_pg.scaffold_22000003
JGI *B. floridae* v1.0 annotation	fgenesh2_pg.scaffold_22000004
JGI *B. floridae* v1.0 annotation	fgenesh2_pg.scaffold_229000061
JGI *B. floridae* v1.0 annotation	fgenesh2_pg.scaffold_23000133
JGI *B. floridae* v1.0 annotation	fgenesh2_pg.scaffold_252000045
JGI *B. floridae* v1.0 annotation	fgenesh2_pg.scaffold_253000048
JGI *B. floridae* v1.0 annotation	fgenesh2_pg.scaffold_253000049
JGI *B. floridae* v1.0 annotation	fgenesh2_pg.scaffold_253000050
JGI *B. floridae* v1.0 annotation	fgenesh2_pg.scaffold_253000051
JGI *B. floridae* v1.0 annotation	fgenesh2_pg.scaffold_253000052
JGI *B. floridae* v1.0 annotation	fgenesh2_pg.scaffold_253000054
JGI *B. floridae* v1.0 annotation	fgenesh2_pg.scaffold_253000055
JGI *B. floridae* v1.0 annotation	fgenesh2_pg.scaffold_269000046
JGI *B. floridae* v1.0 annotation	fgenesh2_pg.scaffold_290000018
JGI *B. floridae* v1.0 annotation	fgenesh2_pg.scaffold_290000019
JGI *B. floridae* v1.0 annotation	fgenesh2_pg.scaffold_302000017
JGI *B. floridae* v1.0 annotation	fgenesh2_pg.scaffold_312000041
JGI *B. floridae* v1.0 annotation	fgenesh2_pg.scaffold_317000043
JGI *B. floridae* v1.0 annotation	fgenesh2_pg.scaffold_317000045
JGI *B. floridae* v1.0 annotation	fgenesh2_pg.scaffold_344000020
JGI *B. floridae* v1.0 annotation	fgenesh2_pg.scaffold_344000021
JGI *B. floridae* v1.0 annotation	fgenesh2_pg.scaffold_344000022
JGI *B. floridae* v1.0 annotation	fgenesh2_pg.scaffold_344000023
JGI *B. floridae* v1.0 annotation	fgenesh2_pg.scaffold_371000017
JGI *B. floridae* v1.0 annotation	fgenesh2_pg.scaffold_371000018
JGI *B. floridae* v1.0 annotation	fgenesh2_pg.scaffold_377000005
JGI *B. floridae* v1.0 annotation	fgenesh2_pg.scaffold_377000006
JGI *B. floridae* v1.0 annotation	fgenesh2_pg.scaffold_39000118
JGI *B. floridae* v1.0 annotation	fgenesh2_pg.scaffold_4000241
JGI *B. floridae* v1.0 annotation	fgenesh2_pg.scaffold_437000014
JGI *B. floridae* v1.0 annotation	fgenesh2_pg.scaffold_444000045
JGI *B. floridae* v1.0 annotation	fgenesh2_pg.scaffold_52000071
JGI *B. floridae* v1.0 annotation	fgenesh2_pg.scaffold_62000043
JGI *B. floridae* v1.0 annotation	fgenesh2_pg.scaffold_67000004
JGI *B. floridae* v1.0 annotation	fgenesh2_pg.scaffold_7000192
JGI *B. floridae* v1.0 annotation	fgenesh2_pg.scaffold_73000041
JGI *B. floridae* v1.0 annotation	fgenesh2_pg.scaffold_75000105

	JGI *B. floridae* v1.0 annotation	fgenesh2_pg.scaffold_862000001
	JGI *B. floridae* v1.0 annotation	fgenesh2_pg.scaffold_929000001
	JGI *B. floridae* v1.0 annotation	fgenesh2_pg.scaffold_99000003
C. intestinalis (transparent sea squirt)	Ensembl release 38 'pep.abinitio'	GENEFINDER00000103062
	Ensembl release 38 'pep.abinitio'	GENSCAN00000077531
	Ensembl release 38 'pep.abinitio'	GENSCAN00000077820
	Ensembl release 38 'pep.abinitio'	GENSCAN00000078509
	Ensembl release 38 'pep.abinitio'	GENSCAN00000080663
	Ensembl release 38 'pep.abinitio'	GENSCAN00000080805
	Ensembl release 38 'pep.abinitio'	GENSCAN00000081083
	Ensembl release 38 'pep.abinitio'	GENSCAN00000081135
	Ensembl release 38 'pep.abinitio'	GENSCAN00000081180
	Ensembl release 38 'pep.abinitio'	GENSCAN00000081263
	Ensembl release 38 'pep.abinitio'	GENSCAN00000081587
	Ensembl release 38 'pep.abinitio'	GENSCAN00000083675
	Ensembl release 38 'pep.abinitio'	GENSCAN00000084617
	Ensembl release 38 'pep.abinitio'	GENSCAN00000084811
	Ensembl release 38 'pep.abinitio'	GENSCAN00000084814
	Ensembl release 38 'pep.abinitio'	GENSCAN00000087200
	Ensembl release 38 'pep.abinitio'	GENSCAN00000088102
	Ensembl release 38 'pep.abinitio'	GENSCAN00000088107
	Ensembl release 38 'pep.abinitio'	GENSCAN00000088147
	Ensembl release 38 'pep.abinitio'	GENSCAN00000088157
	Ensembl release 38 'pep.abinitio'	GENSCAN00000088188
	Ensembl release 38 'pep.abinitio'	GENSCAN00000088192
	Ensembl release 38 'pep.abinitio'	GENSCAN00000088703
	Ensembl release 38 'pep.abinitio'	GENSCAN00000089014
	Ensembl release 38 'pep.abinitio'	GENSCAN00000089929
	Ensembl release 38 'pep.abinitio'	GENSCAN00000090765
	Ensembl release 38 'pep.abinitio'	GENSCAN00000092879
	Ensembl release 38 'pep.abinitio'	GENSCAN00000092883
	Ensembl release 38 'pep.abinitio'	GENSCAN00000093556
	Ensembl release 38 'pep.abinitio'	GENSCAN00000093819
	Ensembl release 38 'pep.abinitio'	GENSCAN00000094267
	Ensembl release 38 'pep.abinitio'	GENSCAN00000094367
	Ensembl release 38 'pep.abinitio'	GENSCAN00000094602
	Ensembl release 38 'pep.abinitio'	GENSCAN00000094610
	Ensembl release 38 'pep.abinitio'	GENSCAN00000095386
	Ensembl release 38 'pep.abinitio'	GENSCAN00000096219
	Ensembl release 38 'pep.abinitio'	GENSCAN00000096363
	Ensembl release 38 'pep.abinitio'	GENSCAN00000097885
	Ensembl release 38 'pep.abinitio'	GENSCAN00000099153
	Ensembl release 38 'pep.abinitio'	GENSCAN00000099417

	Ensembl release 38 'pep.abinitio'	GENSCAN00000099846
	Ensembl release 38 'pep.abinitio'	GENSCAN00000100461
	Ensembl release 38 'pep.abinitio'	GENSCAN00000100479
	Ensembl release 38 'pep.abinitio'	GENSCAN00000102260
	Ensembl release 38 'pep.abinitio'	SNAP_CIONA00000038625
C. intestinalis (transparent sea squirt)	Ensembl release 38 'pep'	ENSCINP00000006173
	Ensembl release 38 'pep'	ENSCINP00000021190
	Ensembl release 38 'pep'	ENSCINP00000023281
	Ensembl release 38 'pep'	ENSCINP00000024089
	Ensembl release 38 'pep'	ENSCINP00000025840
	Ensembl release 38 'pep'	ENSCINP00000026828
	Ensembl release 38 'pep'	ENSCINP00000027491
	Ensembl release 38 'pep'	ENSCINP00000027614
S. purpuratus (purple sea urchin)	HGSC Spur_v2.0 assembly (genscan result)	Scaffold_v2_10414_genscan_15
	HGSC Spur_v2.0 assembly (genscan result)	Scaffold_v2_10489_genscan_2
	HGSC Spur_v2.0 assembly (genscan result)	Scaffold_v2_10511_genscan_1
	HGSC Spur_v2.0 assembly (genscan result)	Scaffold_v2_11349_genscan_2
	HGSC Spur_v2.0 assembly (genscan result)	Scaffold_v2_12165_genscan_5
	HGSC Spur_v2.0 assembly (genscan result)	Scaffold_v2_12167_genscan_1
	HGSC Spur_v2.0 assembly (genscan result)	Scaffold_v2_12710_genscan_1
	HGSC Spur_v2.0 assembly (genscan result)	Scaffold_v2_12736_genscan_10
	HGSC Spur_v2.0 assembly (genscan result)	Scaffold_v2_12740_genscan_7
	HGSC Spur_v2.0 assembly (genscan result)	Scaffold_v2_12740_genscan_11
	HGSC Spur_v2.0 assembly (genscan result)	Scaffold_v2_12759_genscan_2
	HGSC Spur_v2.0 assembly (genscan result)	Scaffold_v2_13740_genscan_1
	HGSC Spur_v2.0 assembly (genscan result)	Scaffold_v2_13748_genscan_4
	HGSC Spur_v2.0 assembly (genscan result)	Scaffold_v2_140_genscan_1
	HGSC Spur_v2.0 assembly (genscan result)	Scaffold_v2_14216_genscan_1
	HGSC Spur_v2.0 assembly (genscan result)	Scaffold_v2_14517_genscan_2
	HGSC Spur_v2.0 assembly (genscan result)	Scaffold_v2_14522_genscan_1
	HGSC Spur_v2.0 assembly (genscan result)	Scaffold_v2_14522_genscan_4
	HGSC Spur_v2.0 assembly (genscan result)	Scaffold_v2_14543_genscan_5
	HGSC Spur_v2.0 assembly (genscan result)	Scaffold_v2_15130_genscan_1
	HGSC Spur_v2.0 assembly (genscan result)	Scaffold_v2_15458_genscan_7
	HGSC Spur_v2.0 assembly (genscan result)	Scaffold_v2_15493_genscan_3
	HGSC Spur_v2.0 assembly (genscan result)	Scaffold_v2_16305_genscan_14
	HGSC Spur_v2.0 assembly (genscan result)	Scaffold_v2_16317_genscan_1
	HGSC Spur_v2.0 assembly (genscan result)	Scaffold_v2_16322_genscan_1
	HGSC Spur_v2.0 assembly (genscan result)	Scaffold_v2_16332_genscan_1
	HGSC Spur_v2.0 assembly (genscan result)	Scaffold_v2_16332_genscan_2
	HGSC Spur_v2.0 assembly (genscan result)	Scaffold_v2_1707_genscan_2
	HGSC Spur_v2.0 assembly (genscan result)	Scaffold_v2_17186_genscan_3

HGSC Spur_v2.0 assembly (genscan result)	Scaffold_v2_17186_genscan_5
HGSC Spur_v2.0 assembly (genscan result)	Scaffold_v2_17255_genscan_2
HGSC Spur_v2.0 assembly (genscan result)	Scaffold_v2_17288_genscan_1
HGSC Spur_v2.0 assembly (genscan result)	Scaffold_v2_17313_genscan_1
HGSC Spur_v2.0 assembly (genscan result)	Scaffold_v2_18023_genscan_5
HGSC Spur_v2.0 assembly (genscan result)	Scaffold_v2_18028_genscan_3
HGSC Spur_v2.0 assembly (genscan result)	Scaffold_v2_19100_genscan_1
HGSC Spur_v2.0 assembly (genscan result)	Scaffold_v2_19970_genscan_6
HGSC Spur_v2.0 assembly (genscan result)	Scaffold_v2_20012_genscan_3
HGSC Spur_v2.0 assembly (genscan result)	Scaffold_v2_20805_genscan_4
HGSC Spur_v2.0 assembly (genscan result)	Scaffold_v2_20823_genscan_4
HGSC Spur_v2.0 assembly (genscan result)	Scaffold_v2_20887_genscan_1
HGSC Spur_v2.0 assembly (genscan result)	Scaffold_v2_20917_genscan_1
HGSC Spur_v2.0 assembly (genscan result)	Scaffold_v2_21543_genscan_1
HGSC Spur_v2.0 assembly (genscan result)	Scaffold_v2_22362_genscan_1
HGSC Spur_v2.0 assembly (genscan result)	Scaffold_v2_22989_genscan_1
HGSC Spur_v2.0 assembly (genscan result)	Scaffold_v2_23006_genscan_1
HGSC Spur_v2.0 assembly (genscan result)	Scaffold_v2_23040_genscan_1
HGSC Spur_v2.0 assembly (genscan result)	Scaffold_v2_23088_genscan_1
HGSC Spur_v2.0 assembly (genscan result)	Scaffold_v2_2320_genscan_1
HGSC Spur_v2.0 assembly (genscan result)	Scaffold_v2_23796_genscan_4
HGSC Spur_v2.0 assembly (genscan result)	Scaffold_v2_23812_genscan_6
HGSC Spur_v2.0 assembly (genscan result)	Scaffold_v2_23819_genscan_2
HGSC Spur_v2.0 assembly (genscan result)	Scaffold_v2_23840_genscan_1
HGSC Spur_v2.0 assembly (genscan result)	Scaffold_v2_2420_genscan_2
HGSC Spur_v2.0 assembly (genscan result)	Scaffold_v2_2440_genscan_1
HGSC Spur_v2.0 assembly (genscan result)	Scaffold_v2_24479_genscan_1
HGSC Spur_v2.0 assembly (genscan result)	Scaffold_v2_2455_genscan_1
HGSC Spur_v2.0 assembly (genscan result)	Scaffold_v2_24589_genscan_2
HGSC Spur_v2.0 assembly (genscan result)	Scaffold_v2_25081_genscan_2
HGSC Spur_v2.0 assembly (genscan result)	Scaffold_v2_25664_genscan_1
HGSC Spur_v2.0 assembly (genscan result)	Scaffold_v2_26364_genscan_3
HGSC Spur_v2.0 assembly (genscan result)	Scaffold_v2_26366_genscan_1
HGSC Spur_v2.0 assembly (genscan result)	Scaffold_v2_26367_genscan_3
HGSC Spur_v2.0 assembly (genscan result)	Scaffold_v2_26655_genscan_1
HGSC Spur_v2.0 assembly (genscan result)	Scaffold_v2_27021_genscan_2
HGSC Spur_v2.0 assembly (genscan result)	Scaffold_v2_27025_genscan_1
HGSC Spur_v2.0 assembly (genscan result)	Scaffold_v2_27055_genscan_1
HGSC Spur_v2.0 assembly (genscan result)	Scaffold_v2_27458_genscan_1
HGSC Spur_v2.0 assembly (genscan result)	Scaffold_v2_28085_genscan_3
HGSC Spur_v2.0 assembly (genscan result)	Scaffold_v2_28085_genscan_7
HGSC Spur_v2.0 assembly (genscan result)	Scaffold_v2_28087_genscan_4
HGSC Spur_v2.0 assembly (genscan result)	Scaffold_v2_28090_genscan_5

HGSC Spur_v2.0 assembly (genscan result)	Scaffold_v2_28090_genscan_7
HGSC Spur_v2.0 assembly (genscan result)	Scaffold_v2_28098_genscan_1
HGSC Spur_v2.0 assembly (genscan result)	Scaffold_v2_28157_genscan_1
HGSC Spur_v2.0 assembly (genscan result)	Scaffold_v2_28817_genscan_2
HGSC Spur_v2.0 assembly (genscan result)	Scaffold_v2_29780_genscan_4
HGSC Spur_v2.0 assembly (genscan result)	Scaffold_v2_29780_genscan_8
HGSC Spur_v2.0 assembly (genscan result)	Scaffold_v2_29804_genscan_2
HGSC Spur_v2.0 assembly (genscan result)	Scaffold_v2_30619_genscan_2
HGSC Spur_v2.0 assembly (genscan result)	Scaffold_v2_30669_genscan_1
HGSC Spur_v2.0 assembly (genscan result)	Scaffold_v2_31377_genscan_36
HGSC Spur_v2.0 assembly (genscan result)	Scaffold_v2_31379_genscan_27
HGSC Spur_v2.0 assembly (genscan result)	Scaffold_v2_31858_genscan_1
HGSC Spur_v2.0 assembly (genscan result)	Scaffold_v2_32333_genscan_4
HGSC Spur_v2.0 assembly (genscan result)	Scaffold_v2_32338_genscan_6
HGSC Spur_v2.0 assembly (genscan result)	Scaffold_v2_32350_genscan_6
HGSC Spur_v2.0 assembly (genscan result)	Scaffold_v2_32393_genscan_1
HGSC Spur_v2.0 assembly (genscan result)	Scaffold_v2_32404_genscan_3
HGSC Spur_v2.0 assembly (genscan result)	Scaffold_v2_33329_genscan_2
HGSC Spur_v2.0 assembly (genscan result)	Scaffold_v2_33368_genscan_1
HGSC Spur_v2.0 assembly (genscan result)	Scaffold_v2_34023_genscan_12
HGSC Spur_v2.0 assembly (genscan result)	Scaffold_v2_34024_genscan_20
HGSC Spur_v2.0 assembly (genscan result)	Scaffold_v2_3409_genscan_1
HGSC Spur_v2.0 assembly (genscan result)	Scaffold_v2_3409_genscan_15
HGSC Spur_v2.0 assembly (genscan result)	Scaffold_v2_3451_genscan_2
HGSC Spur_v2.0 assembly (genscan result)	Scaffold_v2_34528_genscan_1
HGSC Spur_v2.0 assembly (genscan result)	Scaffold_v2_34693_genscan_70
HGSC Spur_v2.0 assembly (genscan result)	Scaffold_v2_34798_genscan_1
HGSC Spur_v2.0 assembly (genscan result)	Scaffold_v2_35449_genscan_1
HGSC Spur_v2.0 assembly (genscan result)	Scaffold_v2_36392_genscan_1
HGSC Spur_v2.0 assembly (genscan result)	Scaffold_v2_36408_genscan_2
HGSC Spur_v2.0 assembly (genscan result)	Scaffold_v2_36451_genscan_1
HGSC Spur_v2.0 assembly (genscan result)	Scaffold_v2_37250_genscan_2
HGSC Spur_v2.0 assembly (genscan result)	Scaffold_v2_37299_genscan_2
HGSC Spur_v2.0 assembly (genscan result)	Scaffold_v2_37344_genscan_2
HGSC Spur_v2.0 assembly (genscan result)	Scaffold_v2_39235_genscan_1
HGSC Spur_v2.0 assembly (genscan result)	Scaffold_v2_39362_genscan_2
HGSC Spur_v2.0 assembly (genscan result)	Scaffold_v2_39383_genscan_1
HGSC Spur_v2.0 assembly (genscan result)	Scaffold_v2_40038_genscan_2
HGSC Spur_v2.0 assembly (genscan result)	Scaffold_v2_40042_genscan_1
HGSC Spur_v2.0 assembly (genscan result)	Scaffold_v2_40073_genscan_1
HGSC Spur_v2.0 assembly (genscan result)	Scaffold_v2_41305_genscan_1
HGSC Spur_v2.0 assembly (genscan result)	Scaffold_v2_41638_genscan_4
HGSC Spur_v2.0 assembly (genscan result)	Scaffold_v2_41665_genscan_1

HGSC Spur_v2.0 assembly (genscan result)	Scaffold_v2_41851_genscan_1
HGSC Spur_v2.0 assembly (genscan result)	Scaffold_v2_42453_genscan_2
HGSC Spur_v2.0 assembly (genscan result)	Scaffold_v2_42466_genscan_1
HGSC Spur_v2.0 assembly (genscan result)	Scaffold_v2_4301_genscan_16
HGSC Spur_v2.0 assembly (genscan result)	Scaffold_v2_4320_genscan_5
HGSC Spur_v2.0 assembly (genscan result)	Scaffold_v2_43339_genscan_1
HGSC Spur_v2.0 assembly (genscan result)	Scaffold_v2_43351_genscan_3
HGSC Spur_v2.0 assembly (genscan result)	Scaffold_v2_43505_genscan_1
HGSC Spur_v2.0 assembly (genscan result)	Scaffold_v2_43908_genscan_1
HGSC Spur_v2.0 assembly (genscan result)	Scaffold_v2_44179_genscan_2
HGSC Spur_v2.0 assembly (genscan result)	Scaffold_v2_44503_genscan_10
HGSC Spur_v2.0 assembly (genscan result)	Scaffold_v2_44576_genscan_1
HGSC Spur_v2.0 assembly (genscan result)	Scaffold_v2_45213_genscan_12
HGSC Spur_v2.0 assembly (genscan result)	Scaffold_v2_45221_genscan_1
HGSC Spur_v2.0 assembly (genscan result)	Scaffold_v2_45260_genscan_4
HGSC Spur_v2.0 assembly (genscan result)	Scaffold_v2_45356_genscan_1
HGSC Spur_v2.0 assembly (genscan result)	Scaffold_v2_45600_genscan_1
HGSC Spur_v2.0 assembly (genscan result)	Scaffold_v2_46118_genscan_2
HGSC Spur_v2.0 assembly (genscan result)	Scaffold_v2_4661_genscan_1
HGSC Spur_v2.0 assembly (genscan result)	Scaffold_v2_47500_genscan_1
HGSC Spur_v2.0 assembly (genscan result)	Scaffold_v2_47551_genscan_1
HGSC Spur_v2.0 assembly (genscan result)	Scaffold_v2_48168_genscan_1
HGSC Spur_v2.0 assembly (genscan result)	Scaffold_v2_48183_genscan_1
HGSC Spur_v2.0 assembly (genscan result)	Scaffold_v2_48364_genscan_1
HGSC Spur_v2.0 assembly (genscan result)	Scaffold_v2_48970_genscan_2
HGSC Spur_v2.0 assembly (genscan result)	Scaffold_v2_48973_genscan_6
HGSC Spur_v2.0 assembly (genscan result)	Scaffold_v2_48981_genscan_3
HGSC Spur_v2.0 assembly (genscan result)	Scaffold_v2_49010_genscan_1
HGSC Spur_v2.0 assembly (genscan result)	Scaffold_v2_49024_genscan_1
HGSC Spur_v2.0 assembly (genscan result)	Scaffold_v2_4914_genscan_15
HGSC Spur_v2.0 assembly (genscan result)	Scaffold_v2_4953_genscan_1
HGSC Spur_v2.0 assembly (genscan result)	Scaffold_v2_50144_genscan_34
HGSC Spur_v2.0 assembly (genscan result)	Scaffold_v2_50181_genscan_4
HGSC Spur_v2.0 assembly (genscan result)	Scaffold_v2_50950_genscan_5
HGSC Spur_v2.0 assembly (genscan result)	Scaffold_v2_50956_genscan_2
HGSC Spur_v2.0 assembly (genscan result)	Scaffold_v2_51007_genscan_1
HGSC Spur_v2.0 assembly (genscan result)	Scaffold_v2_51834_genscan_1
HGSC Spur_v2.0 assembly (genscan result)	Scaffold_v2_51880_genscan_1
HGSC Spur_v2.0 assembly (genscan result)	Scaffold_v2_52510_genscan_4
HGSC Spur_v2.0 assembly (genscan result)	Scaffold_v2_53427_genscan_1
HGSC Spur_v2.0 assembly (genscan result)	Scaffold_v2_55216_genscan_1
HGSC Spur_v2.0 assembly (genscan result)	Scaffold_v2_55236_genscan_3
HGSC Spur_v2.0 assembly (genscan result)	Scaffold_v2_55356_genscan_1

HGSC Spur_v2.0 assembly (genscan result)	Scaffold_v2_55386_genscan_1
HGSC Spur_v2.0 assembly (genscan result)	Scaffold_v2_55868_genscan_1
HGSC Spur_v2.0 assembly (genscan result)	Scaffold_v2_56606_genscan_12
HGSC Spur_v2.0 assembly (genscan result)	Scaffold_v2_56607_genscan_14
HGSC Spur_v2.0 assembly (genscan result)	Scaffold_v2_56617_genscan_6
HGSC Spur_v2.0 assembly (genscan result)	Scaffold_v2_56642_genscan_2
HGSC Spur_v2.0 assembly (genscan result)	Scaffold_v2_57313_genscan_3
HGSC Spur_v2.0 assembly (genscan result)	Scaffold_v2_57313_genscan_5
HGSC Spur_v2.0 assembly (genscan result)	Scaffold_v2_57333_genscan_1
HGSC Spur_v2.0 assembly (genscan result)	Scaffold_v2_57338_genscan_3
HGSC Spur_v2.0 assembly (genscan result)	Scaffold_v2_57395_genscan_1
HGSC Spur_v2.0 assembly (genscan result)	Scaffold_v2_57984_genscan_11
HGSC Spur_v2.0 assembly (genscan result)	Scaffold_v2_57984_genscan_20
HGSC Spur_v2.0 assembly (genscan result)	Scaffold_v2_57989_genscan_10
HGSC Spur_v2.0 assembly (genscan result)	Scaffold_v2_58011_genscan_1
HGSC Spur_v2.0 assembly (genscan result)	Scaffold_v2_5802_genscan_1
HGSC Spur_v2.0 assembly (genscan result)	Scaffold_v2_5802_genscan_3
HGSC Spur_v2.0 assembly (genscan result)	Scaffold_v2_58033_genscan_2
HGSC Spur_v2.0 assembly (genscan result)	Scaffold_v2_58053_genscan_3
HGSC Spur_v2.0 assembly (genscan result)	Scaffold_v2_5819_genscan_1
HGSC Spur_v2.0 assembly (genscan result)	Scaffold_v2_58230_genscan_1
HGSC Spur_v2.0 assembly (genscan result)	Scaffold_v2_58801_genscan_1
HGSC Spur_v2.0 assembly (genscan result)	Scaffold_v2_58899_genscan_1
HGSC Spur_v2.0 assembly (genscan result)	Scaffold_v2_59540_genscan_79
HGSC Spur_v2.0 assembly (genscan result)	Scaffold_v2_59573_genscan_1
HGSC Spur_v2.0 assembly (genscan result)	Scaffold_v2_59587_genscan_3
HGSC Spur_v2.0 assembly (genscan result)	Scaffold_v2_6_genscan_17
HGSC Spur_v2.0 assembly (genscan result)	Scaffold_v2_60310_genscan_4
HGSC Spur_v2.0 assembly (genscan result)	Scaffold_v2_60334_genscan_1
HGSC Spur_v2.0 assembly (genscan result)	Scaffold_v2_61111_genscan_1
HGSC Spur_v2.0 assembly (genscan result)	Scaffold_v2_61191_genscan_1
HGSC Spur_v2.0 assembly (genscan result)	Scaffold_v2_61305_genscan_1
HGSC Spur_v2.0 assembly (genscan result)	Scaffold_v2_61384_genscan_2
HGSC Spur_v2.0 assembly (genscan result)	Scaffold_v2_62_genscan_1
HGSC Spur_v2.0 assembly (genscan result)	Scaffold_v2_62067_genscan_9
HGSC Spur_v2.0 assembly (genscan result)	Scaffold_v2_62090_genscan_3
HGSC Spur_v2.0 assembly (genscan result)	Scaffold_v2_62111_genscan_1
HGSC Spur_v2.0 assembly (genscan result)	Scaffold_v2_62116_genscan_1
HGSC Spur_v2.0 assembly (genscan result)	Scaffold_v2_62135_genscan_1
HGSC Spur_v2.0 assembly (genscan result)	Scaffold_v2_62894_genscan_22
HGSC Spur_v2.0 assembly (genscan result)	Scaffold_v2_62903_genscan_11
HGSC Spur_v2.0 assembly (genscan result)	Scaffold_v2_63832_genscan_2
HGSC Spur_v2.0 assembly (genscan result)	Scaffold_v2_64463_genscan_1

HGSC Spur_v2.0 assembly (genscan result)	Scaffold_v2_64463_genscan_7
HGSC Spur_v2.0 assembly (genscan result)	Scaffold_v2_64512_genscan_1
HGSC Spur_v2.0 assembly (genscan result)	Scaffold_v2_6483_genscan_1
HGSC Spur_v2.0 assembly (genscan result)	Scaffold_v2_6493_genscan_1
HGSC Spur_v2.0 assembly (genscan result)	Scaffold_v2_65158_genscan_5
HGSC Spur_v2.0 assembly (genscan result)	Scaffold_v2_65176_genscan_2
HGSC Spur_v2.0 assembly (genscan result)	Scaffold_v2_65179_genscan_1
HGSC Spur_v2.0 assembly (genscan result)	Scaffold_v2_65194_genscan_1
HGSC Spur_v2.0 assembly (genscan result)	Scaffold_v2_65233_genscan_2
HGSC Spur_v2.0 assembly (genscan result)	Scaffold_v2_65883_genscan_1
HGSC Spur_v2.0 assembly (genscan result)	Scaffold_v2_65899_genscan_1
HGSC Spur_v2.0 assembly (genscan result)	Scaffold_v2_65931_genscan_1
HGSC Spur_v2.0 assembly (genscan result)	Scaffold_v2_66668_genscan_1
HGSC Spur_v2.0 assembly (genscan result)	Scaffold_v2_66689_genscan_2
HGSC Spur_v2.0 assembly (genscan result)	Scaffold_v2_66694_genscan_1
HGSC Spur_v2.0 assembly (genscan result)	Scaffold_v2_67888_genscan_15
HGSC Spur_v2.0 assembly (genscan result)	Scaffold_v2_67890_genscan_12
HGSC Spur_v2.0 assembly (genscan result)	Scaffold_v2_67893_genscan_9
HGSC Spur_v2.0 assembly (genscan result)	Scaffold_v2_67988_genscan_1
HGSC Spur_v2.0 assembly (genscan result)	Scaffold_v2_68068_genscan_1
HGSC Spur_v2.0 assembly (genscan result)	Scaffold_v2_68405_genscan_3
HGSC Spur_v2.0 assembly (genscan result)	Scaffold_v2_68408_genscan_1
HGSC Spur_v2.0 assembly (genscan result)	Scaffold_v2_68438_genscan_2
HGSC Spur_v2.0 assembly (genscan result)	Scaffold_v2_69289_genscan_2
HGSC Spur_v2.0 assembly (genscan result)	Scaffold_v2_69329_genscan_1
HGSC Spur_v2.0 assembly (genscan result)	Scaffold_v2_69893_genscan_2
HGSC Spur_v2.0 assembly (genscan result)	Scaffold_v2_69893_genscan_9
HGSC Spur_v2.0 assembly (genscan result)	Scaffold_v2_69944_genscan_1
HGSC Spur_v2.0 assembly (genscan result)	Scaffold_v2_70594_genscan_6
HGSC Spur_v2.0 assembly (genscan result)	Scaffold_v2_70596_genscan_9
HGSC Spur_v2.0 assembly (genscan result)	Scaffold_v2_70600_genscan_15
HGSC Spur_v2.0 assembly (genscan result)	Scaffold_v2_70607_genscan_1
HGSC Spur_v2.0 assembly (genscan result)	Scaffold_v2_70618_genscan_2
HGSC Spur_v2.0 assembly (genscan result)	Scaffold_v2_70618_genscan_4
HGSC Spur_v2.0 assembly (genscan result)	Scaffold_v2_70625_genscan_3
HGSC Spur_v2.0 assembly (genscan result)	Scaffold_v2_70650_genscan_1
HGSC Spur_v2.0 assembly (genscan result)	Scaffold_v2_70666_genscan_1
HGSC Spur_v2.0 assembly (genscan result)	Scaffold_v2_71276_genscan_3
HGSC Spur_v2.0 assembly (genscan result)	Scaffold_v2_71286_genscan_1
HGSC Spur_v2.0 assembly (genscan result)	Scaffold_v2_7178_genscan_4
HGSC Spur_v2.0 assembly (genscan result)	Scaffold_v2_7181_genscan_6
HGSC Spur_v2.0 assembly (genscan result)	Scaffold_v2_7181_genscan_8
HGSC Spur_v2.0 assembly (genscan result)	Scaffold_v2_71911_genscan_3

HGSC Spur_v2.0 assembly (genscan result)	Scaffold_v2_71911_genscan_4
HGSC Spur_v2.0 assembly (genscan result)	Scaffold_v2_71919_genscan_10
HGSC Spur_v2.0 assembly (genscan result)	Scaffold_v2_71939_genscan_1
HGSC Spur_v2.0 assembly (genscan result)	Scaffold_v2_72747_genscan_4
HGSC Spur_v2.0 assembly (genscan result)	Scaffold_v2_73334_genscan_41
HGSC Spur_v2.0 assembly (genscan result)	Scaffold_v2_73360_genscan_4
HGSC Spur_v2.0 assembly (genscan result)	Scaffold_v2_74148_genscan_1
HGSC Spur_v2.0 assembly (genscan result)	Scaffold_v2_74949_genscan_3
HGSC Spur_v2.0 assembly (genscan result)	Scaffold_v2_74949_genscan_5
HGSC Spur_v2.0 assembly (genscan result)	Scaffold_v2_74957_genscan_2
HGSC Spur_v2.0 assembly (genscan result)	Scaffold_v2_74970_genscan_1
HGSC Spur_v2.0 assembly (genscan result)	Scaffold_v2_75014_genscan_1
HGSC Spur_v2.0 assembly (genscan result)	Scaffold_v2_75019_genscan_1
HGSC Spur_v2.0 assembly (genscan result)	Scaffold_v2_75773_genscan_33
HGSC Spur_v2.0 assembly (genscan result)	Scaffold_v2_75782_genscan_2
HGSC Spur_v2.0 assembly (genscan result)	Scaffold_v2_75783_genscan_3
HGSC Spur_v2.0 assembly (genscan result)	Scaffold_v2_75783_genscan_5
HGSC Spur_v2.0 assembly (genscan result)	Scaffold_v2_75790_genscan_1
HGSC Spur_v2.0 assembly (genscan result)	Scaffold_v2_75869_genscan_3
HGSC Spur_v2.0 assembly (genscan result)	Scaffold_v2_76009_genscan_1
HGSC Spur_v2.0 assembly (genscan result)	Scaffold_v2_76987_genscan_1
HGSC Spur_v2.0 assembly (genscan result)	Scaffold_v2_77015_genscan_1
HGSC Spur_v2.0 assembly (genscan result)	Scaffold_v2_77104_genscan_1
HGSC Spur_v2.0 assembly (genscan result)	Scaffold_v2_77843_genscan_1
HGSC Spur_v2.0 assembly (genscan result)	Scaffold_v2_77852_genscan_1
HGSC Spur_v2.0 assembly (genscan result)	Scaffold_v2_78432_genscan_2
HGSC Spur_v2.0 assembly (genscan result)	Scaffold_v2_78469_genscan_1
HGSC Spur_v2.0 assembly (genscan result)	Scaffold_v2_78576_genscan_1
HGSC Spur_v2.0 assembly (genscan result)	Scaffold_v2_78944_genscan_1
HGSC Spur_v2.0 assembly (genscan result)	Scaffold_v2_79438_genscan_1
HGSC Spur_v2.0 assembly (genscan result)	Scaffold_v2_79474_genscan_1
HGSC Spur_v2.0 assembly (genscan result)	Scaffold_v2_79557_genscan_1
HGSC Spur_v2.0 assembly (genscan result)	Scaffold_v2_80270_genscan_35
HGSC Spur_v2.0 assembly (genscan result)	Scaffold_v2_80293_genscan_1
HGSC Spur_v2.0 assembly (genscan result)	Scaffold_v2_80897_genscan_2
HGSC Spur_v2.0 assembly (genscan result)	Scaffold_v2_8124_genscan_6
HGSC Spur_v2.0 assembly (genscan result)	Scaffold_v2_81741_genscan_1
HGSC Spur_v2.0 assembly (genscan result)	Scaffold_v2_81756_genscan_4
HGSC Spur_v2.0 assembly (genscan result)	Scaffold_v2_81772_genscan_1
HGSC Spur_v2.0 assembly (genscan result)	Scaffold_v2_82264_genscan_5
HGSC Spur_v2.0 assembly (genscan result)	Scaffold_v2_82274_genscan_2
HGSC Spur_v2.0 assembly (genscan result)	Scaffold_v2_82299_genscan_1
HGSC Spur_v2.0 assembly (genscan result)	Scaffold_v2_82300_genscan_6

	HGSC Spur_v2.0 assembly (genscan result)	Scaffold_v2_82342_genscan_2
	HGSC Spur_v2.0 assembly (genscan result)	Scaffold_v2_828_genscan_2
	HGSC Spur_v2.0 assembly (genscan result)	Scaffold_v2_83189_genscan_10
	HGSC Spur_v2.0 assembly (genscan result)	Scaffold_v2_83189_genscan_15
	HGSC Spur_v2.0 assembly (genscan result)	Scaffold_v2_83194_genscan_3
	HGSC Spur_v2.0 assembly (genscan result)	Scaffold_v2_83194_genscan_11
	HGSC Spur_v2.0 assembly (genscan result)	Scaffold_v2_83200_genscan_1
	HGSC Spur_v2.0 assembly (genscan result)	Scaffold_v2_84490_genscan_5
	HGSC Spur_v2.0 assembly (genscan result)	Scaffold_v2_84497_genscan_2
	HGSC Spur_v2.0 assembly (genscan result)	Scaffold_v2_84497_genscan_4
	HGSC Spur_v2.0 assembly (genscan result)	Scaffold_v2_85338_genscan_11
	HGSC Spur_v2.0 assembly (genscan result)	Scaffold_v2_85343_genscan_1
	HGSC Spur_v2.0 assembly (genscan result)	Scaffold_v2_85347_genscan_1
	HGSC Spur_v2.0 assembly (genscan result)	Scaffold_v2_85352_genscan_2
	HGSC Spur_v2.0 assembly (genscan result)	Scaffold_v2_85352_genscan_4
	HGSC Spur_v2.0 assembly (genscan result)	Scaffold_v2_85465_genscan_1
	HGSC Spur_v2.0 assembly (genscan result)	Scaffold_v2_8615_genscan_1
	HGSC Spur_v2.0 assembly (genscan result)	Scaffold_v2_86249_genscan_1
	HGSC Spur_v2.0 assembly (genscan result)	Scaffold_v2_87215_genscan_1
	HGSC Spur_v2.0 assembly (genscan result)	Scaffold_v2_87836_genscan_1
	HGSC Spur_v2.0 assembly (genscan result)	Scaffold_v2_88554_genscan_6
	HGSC Spur_v2.0 assembly (genscan result)	Scaffold_v2_88563_genscan_7
	HGSC Spur_v2.0 assembly (genscan result)	Scaffold_v2_88590_genscan_1
	HGSC Spur_v2.0 assembly (genscan result)	Scaffold_v2_89442_genscan_2
	HGSC Spur_v2.0 assembly (genscan result)	Scaffold_v2_89449_genscan_7
	HGSC Spur_v2.0 assembly (genscan result)	Scaffold_v2_89933_genscan_1
	HGSC Spur_v2.0 assembly (genscan result)	Scaffold_v2_8998_genscan_3
	HGSC Spur_v2.0 assembly (genscan result)	Scaffold_v2_9008_genscan_2
	HGSC Spur_v2.0 assembly (genscan result)	Scaffold_v2_9764_genscan_6
	HGSC Spur_v2.0 assembly (genscan result)	Scaffold_v2_9772_genscan_2
	HGSC Spur_v2.0 assembly (genscan result)	Scaffold_v2_9777_genscan_2
	HGSC Spur_v2.0 assembly (genscan result)	Scaffold_v2_9790_genscan_2
	HGSC Spur_v2.0 assembly (genscan result)	Scaffold_v2_9833_genscan_1
S. purpuratus (purple sea urchin)	HGSC *S. purpuratus* GLEAN3	GLEAN3_00001
	HGSC *S. purpuratus* GLEAN3	GLEAN3_00015
	HGSC *S. purpuratus* GLEAN3	GLEAN3_00457
	HGSC *S. purpuratus* GLEAN3	GLEAN3_00523
	HGSC *S. purpuratus* GLEAN3	GLEAN3_00672
	HGSC *S. purpuratus* GLEAN3	GLEAN3_00738
	HGSC *S. purpuratus* GLEAN3	GLEAN3_00816
	HGSC *S. purpuratus* GLEAN3	GLEAN3_00852
	HGSC *S. purpuratus* GLEAN3	GLEAN3_00863
	HGSC *S. purpuratus* GLEAN3	GLEAN3_00896

HGSC *S. purpuratus* GLEAN3	GLEAN3_01016
HGSC *S. purpuratus* GLEAN3	GLEAN3_01054
HGSC *S. purpuratus* GLEAN3	GLEAN3_01210
HGSC *S. purpuratus* GLEAN3	GLEAN3_01444
HGSC *S. purpuratus* GLEAN3	GLEAN3_01548
HGSC *S. purpuratus* GLEAN3	GLEAN3_01591
HGSC *S. purpuratus* GLEAN3	GLEAN3_01608
HGSC *S. purpuratus* GLEAN3	GLEAN3_01781
HGSC *S. purpuratus* GLEAN3	GLEAN3_01884
HGSC *S. purpuratus* GLEAN3	GLEAN3_02086
HGSC *S. purpuratus* GLEAN3	GLEAN3_02272
HGSC *S. purpuratus* GLEAN3	GLEAN3_02372
HGSC *S. purpuratus* GLEAN3	GLEAN3_02436
HGSC *S. purpuratus* GLEAN3	GLEAN3_02641
HGSC *S. purpuratus* GLEAN3	GLEAN3_02758
HGSC *S. purpuratus* GLEAN3	GLEAN3_02868
HGSC *S. purpuratus* GLEAN3	GLEAN3_02888
HGSC *S. purpuratus* GLEAN3	GLEAN3_02962
HGSC *S. purpuratus* GLEAN3	GLEAN3_03186
HGSC *S. purpuratus* GLEAN3	GLEAN3_03200
HGSC *S. purpuratus* GLEAN3	GLEAN3_03247
HGSC *S. purpuratus* GLEAN3	GLEAN3_03303
HGSC *S. purpuratus* GLEAN3	GLEAN3_03366
HGSC *S. purpuratus* GLEAN3	GLEAN3_03539
HGSC *S. purpuratus* GLEAN3	GLEAN3_03553
HGSC *S. purpuratus* GLEAN3	GLEAN3_03640
HGSC *S. purpuratus* GLEAN3	GLEAN3_03715
HGSC *S. purpuratus* GLEAN3	GLEAN3_03762
HGSC *S. purpuratus* GLEAN3	GLEAN3_03797
HGSC *S. purpuratus* GLEAN3	GLEAN3_03934
HGSC *S. purpuratus* GLEAN3	GLEAN3_04012
HGSC *S. purpuratus* GLEAN3	GLEAN3_04043
HGSC *S. purpuratus* GLEAN3	GLEAN3_04053
HGSC *S. purpuratus* GLEAN3	GLEAN3_04165
HGSC *S. purpuratus* GLEAN3	GLEAN3_04343
HGSC *S. purpuratus* GLEAN3	GLEAN3_04872
HGSC *S. purpuratus* GLEAN3	GLEAN3_05301
HGSC *S. purpuratus* GLEAN3	GLEAN3_05383
HGSC *S. purpuratus* GLEAN3	GLEAN3_05410
HGSC *S. purpuratus* GLEAN3	GLEAN3_05462
HGSC *S. purpuratus* GLEAN3	GLEAN3_05581
HGSC *S. purpuratus* GLEAN3	GLEAN3_05609
HGSC *S. purpuratus* GLEAN3	GLEAN3_05732

HGSC *S. purpuratus* GLEAN3	GLEAN3_05993
HGSC *S. purpuratus* GLEAN3	GLEAN3_06016
HGSC *S. purpuratus* GLEAN3	GLEAN3_06203
HGSC *S. purpuratus* GLEAN3	GLEAN3_06229
HGSC *S. purpuratus* GLEAN3	GLEAN3_06456
HGSC *S. purpuratus* GLEAN3	GLEAN3_06610
HGSC *S. purpuratus* GLEAN3	GLEAN3_06733
HGSC *S. purpuratus* GLEAN3	GLEAN3_07446
HGSC *S. purpuratus* GLEAN3	GLEAN3_07561
HGSC *S. purpuratus* GLEAN3	GLEAN3_08029
HGSC *S. purpuratus* GLEAN3	GLEAN3_08283
HGSC *S. purpuratus* GLEAN3	GLEAN3_08382
HGSC *S. purpuratus* GLEAN3	GLEAN3_08431
HGSC *S. purpuratus* GLEAN3	GLEAN3_08442
HGSC *S. purpuratus* GLEAN3	GLEAN3_08498
HGSC *S. purpuratus* GLEAN3	GLEAN3_08547
HGSC *S. purpuratus* GLEAN3	GLEAN3_08597
HGSC *S. purpuratus* GLEAN3	GLEAN3_08707
HGSC *S. purpuratus* GLEAN3	GLEAN3_08833
HGSC *S. purpuratus* GLEAN3	GLEAN3_09017
HGSC *S. purpuratus* GLEAN3	GLEAN3_09111
HGSC *S. purpuratus* GLEAN3	GLEAN3_09488
HGSC *S. purpuratus* GLEAN3	GLEAN3_09659
HGSC *S. purpuratus* GLEAN3	GLEAN3_10039
HGSC *S. purpuratus* GLEAN3	GLEAN3_10053
HGSC *S. purpuratus* GLEAN3	GLEAN3_10091
HGSC *S. purpuratus* GLEAN3	GLEAN3_10097
HGSC *S. purpuratus* GLEAN3	GLEAN3_10153
HGSC *S. purpuratus* GLEAN3	GLEAN3_10667
HGSC *S. purpuratus* GLEAN3	GLEAN3_11088
HGSC *S. purpuratus* GLEAN3	GLEAN3_11097
HGSC *S. purpuratus* GLEAN3	GLEAN3_11439
HGSC *S. purpuratus* GLEAN3	GLEAN3_11441
HGSC *S. purpuratus* GLEAN3	GLEAN3_11776
HGSC *S. purpuratus* GLEAN3	GLEAN3_11827
HGSC *S. purpuratus* GLEAN3	GLEAN3_11855
HGSC *S. purpuratus* GLEAN3	GLEAN3_13038
HGSC *S. purpuratus* GLEAN3	GLEAN3_13206
HGSC *S. purpuratus* GLEAN3	GLEAN3_13465
HGSC *S. purpuratus* GLEAN3	GLEAN3_13504
HGSC *S. purpuratus* GLEAN3	GLEAN3_13521
HGSC *S. purpuratus* GLEAN3	GLEAN3_13665
HGSC *S. purpuratus* GLEAN3	GLEAN3_13952

HGSC *S. purpuratus* GLEAN3	GLEAN3_14112
HGSC *S. purpuratus* GLEAN3	GLEAN3_14122
HGSC *S. purpuratus* GLEAN3	GLEAN3_14128
HGSC *S. purpuratus* GLEAN3	GLEAN3_14495
HGSC *S. purpuratus* GLEAN3	GLEAN3_14503
HGSC *S. purpuratus* GLEAN3	GLEAN3_14530
HGSC *S. purpuratus* GLEAN3	GLEAN3_14761
HGSC *S. purpuratus* GLEAN3	GLEAN3_15033
HGSC *S. purpuratus* GLEAN3	GLEAN3_15052
HGSC *S. purpuratus* GLEAN3	GLEAN3_15105
HGSC *S. purpuratus* GLEAN3	GLEAN3_15205
HGSC *S. purpuratus* GLEAN3	GLEAN3_15206
HGSC *S. purpuratus* GLEAN3	GLEAN3_15298
HGSC *S. purpuratus* GLEAN3	GLEAN3_15340
HGSC *S. purpuratus* GLEAN3	GLEAN3_15481
HGSC *S. purpuratus* GLEAN3	GLEAN3_15972
HGSC *S. purpuratus* GLEAN3	GLEAN3_16060
HGSC *S. purpuratus* GLEAN3	GLEAN3_16257
HGSC *S. purpuratus* GLEAN3	GLEAN3_16759
HGSC *S. purpuratus* GLEAN3	GLEAN3_16783
HGSC *S. purpuratus* GLEAN3	GLEAN3_16794
HGSC *S. purpuratus* GLEAN3	GLEAN3_16810
HGSC *S. purpuratus* GLEAN3	GLEAN3_16921
HGSC *S. purpuratus* GLEAN3	GLEAN3_16926
HGSC *S. purpuratus* GLEAN3	GLEAN3_17038
HGSC *S. purpuratus* GLEAN3	GLEAN3_17054
HGSC *S. purpuratus* GLEAN3	GLEAN3_17196
HGSC *S. purpuratus* GLEAN3	GLEAN3_17245
HGSC *S. purpuratus* GLEAN3	GLEAN3_17341
HGSC *S. purpuratus* GLEAN3	GLEAN3_17505
HGSC *S. purpuratus* GLEAN3	GLEAN3_17708
HGSC *S. purpuratus* GLEAN3	GLEAN3_17993
HGSC *S. purpuratus* GLEAN3	GLEAN3_18384
HGSC *S. purpuratus* GLEAN3	GLEAN3_19347
HGSC *S. purpuratus* GLEAN3	GLEAN3_19497
HGSC *S. purpuratus* GLEAN3	GLEAN3_19696
HGSC *S. purpuratus* GLEAN3	GLEAN3_19699
HGSC *S. purpuratus* GLEAN3	GLEAN3_19700
HGSC *S. purpuratus* GLEAN3	GLEAN3_20240
HGSC *S. purpuratus* GLEAN3	GLEAN3_20380
HGSC *S. purpuratus* GLEAN3	GLEAN3_20436
HGSC *S. purpuratus* GLEAN3	GLEAN3_20561
HGSC *S. purpuratus* GLEAN3	GLEAN3_20569

HGSC *S. purpuratus* GLEAN3	GLEAN3_20661
HGSC *S. purpuratus* GLEAN3	GLEAN3_20916
HGSC *S. purpuratus* GLEAN3	GLEAN3_21243
HGSC *S. purpuratus* GLEAN3	GLEAN3_21447
HGSC *S. purpuratus* GLEAN3	GLEAN3_21478
HGSC *S. purpuratus* GLEAN3	GLEAN3_21844
HGSC *S. purpuratus* GLEAN3	GLEAN3_21930
HGSC *S. purpuratus* GLEAN3	GLEAN3_22001
HGSC *S. purpuratus* GLEAN3	GLEAN3_22130
HGSC *S. purpuratus* GLEAN3	GLEAN3_22294
HGSC *S. purpuratus* GLEAN3	GLEAN3_22394
HGSC *S. purpuratus* GLEAN3	GLEAN3_22412
HGSC *S. purpuratus* GLEAN3	GLEAN3_22442
HGSC *S. purpuratus* GLEAN3	GLEAN3_22564
HGSC *S. purpuratus* GLEAN3	GLEAN3_22780
HGSC *S. purpuratus* GLEAN3	GLEAN3_23120
HGSC *S. purpuratus* GLEAN3	GLEAN3_23183
HGSC *S. purpuratus* GLEAN3	GLEAN3_23474
HGSC *S. purpuratus* GLEAN3	GLEAN3_23532
HGSC *S. purpuratus* GLEAN3	GLEAN3_23550
HGSC *S. purpuratus* GLEAN3	GLEAN3_23642
HGSC *S. purpuratus* GLEAN3	GLEAN3_24020
HGSC *S. purpuratus* GLEAN3	GLEAN3_24649
HGSC *S. purpuratus* GLEAN3	GLEAN3_24709
HGSC *S. purpuratus* GLEAN3	GLEAN3_24975
HGSC *S. purpuratus* GLEAN3	GLEAN3_25077
HGSC *S. purpuratus* GLEAN3	GLEAN3_25138
HGSC *S. purpuratus* GLEAN3	GLEAN3_25166
HGSC *S. purpuratus* GLEAN3	GLEAN3_25167
HGSC *S. purpuratus* GLEAN3	GLEAN3_25179
HGSC *S. purpuratus* GLEAN3	GLEAN3_25204
HGSC *S. purpuratus* GLEAN3	GLEAN3_25600
HGSC *S. purpuratus* GLEAN3	GLEAN3_25680
HGSC *S. purpuratus* GLEAN3	GLEAN3_25914
HGSC *S. purpuratus* GLEAN3	GLEAN3_25948
HGSC *S. purpuratus* GLEAN3	GLEAN3_26020
HGSC *S. purpuratus* GLEAN3	GLEAN3_26036
HGSC *S. purpuratus* GLEAN3	GLEAN3_26189
HGSC *S. purpuratus* GLEAN3	GLEAN3_26304
HGSC *S. purpuratus* GLEAN3	GLEAN3_26400
HGSC *S. purpuratus* GLEAN3	GLEAN3_26622
HGSC *S. purpuratus* GLEAN3	GLEAN3_27035
HGSC *S. purpuratus* GLEAN3	GLEAN3_27207

	HGSC *S. purpuratus* GLEAN3	GLEAN3_27300
	HGSC *S. purpuratus* GLEAN3	GLEAN3_27511
	HGSC *S. purpuratus* GLEAN3	GLEAN3_27513
	HGSC *S. purpuratus* GLEAN3	GLEAN3_27610
	HGSC *S. purpuratus* GLEAN3	GLEAN3_27762
	HGSC *S. purpuratus* GLEAN3	GLEAN3_27808
	HGSC *S. purpuratus* GLEAN3	GLEAN3_27858
	HGSC *S. purpuratus* GLEAN3	GLEAN3_28060
	HGSC *S. purpuratus* GLEAN3	GLEAN3_28294
	HGSC *S. purpuratus* GLEAN3	GLEAN3_28387
	HGSC *S. purpuratus* GLEAN3	GLEAN3_28433
	HGSC *S. purpuratus* GLEAN3	GLEAN3_28483
	HGSC *S. purpuratus* GLEAN3	GLEAN3_28485
	HGSC *S. purpuratus* GLEAN3	GLEAN3_28595
	HGSC *S. purpuratus* GLEAN3	GLEAN3_28630
	HGSC *S. purpuratus* GLEAN3	GLEAN3_28681
	HGSC *S. purpuratus* GLEAN3	GLEAN3_28805
	HGSC *S. purpuratus* GLEAN3	GLEAN3_28820
N. vectensis (sea anemone)	JGI N. vectensis v1.0 assembly (genscan result)	scaffold_101_genscan_6
	JGI N. vectensis v1.0 assembly (genscan result)	scaffold_1163_genscan_1
	JGI N. vectensis v1.0 assembly (genscan result)	scaffold_117_genscan_35
	JGI N. vectensis v1.0 assembly (genscan result)	scaffold_117_genscan_38
	JGI N. vectensis v1.0 assembly (genscan result)	scaffold_1400_genscan_2
	JGI N. vectensis v1.0 assembly (genscan result)	scaffold_150_genscan_22
	JGI N. vectensis v1.0 assembly (genscan result)	scaffold_150_genscan_9
	JGI N. vectensis v1.0 assembly (genscan result)	scaffold_168_genscan_35
	JGI N. vectensis v1.0 assembly (genscan result)	scaffold_179_genscan_43
	JGI N. vectensis v1.0 assembly (genscan result)	scaffold_179_genscan_46
	JGI N. vectensis v1.0 assembly (genscan result)	scaffold_1867_genscan_3
	JGI N. vectensis v1.0 assembly (genscan result)	scaffold_189_genscan_19
	JGI N. vectensis v1.0 assembly (genscan result)	scaffold_189_genscan_29
	JGI N. vectensis v1.0 assembly (genscan result)	scaffold_189_genscan_31
	JGI N. vectensis v1.0 assembly (genscan result)	scaffold_189_genscan_35
	JGI N. vectensis v1.0 assembly (genscan result)	scaffold_192_genscan_42
	JGI N. vectensis v1.0 assembly (genscan result)	scaffold_289_genscan_23
	JGI N. vectensis v1.0 assembly (genscan result)	scaffold_291_genscan_5
	JGI N. vectensis v1.0 assembly (genscan result)	scaffold_319_genscan_9
	JGI N. vectensis v1.0 assembly (genscan result)	scaffold_32_genscan_11
	JGI N. vectensis v1.0 assembly (genscan result)	scaffold_32_genscan_1
	JGI N. vectensis v1.0 assembly (genscan result)	scaffold_32_genscan_5
	JGI N. vectensis v1.0 assembly (genscan result)	scaffold_393_genscan_12
	JGI N. vectensis v1.0 assembly (genscan result)	scaffold_402_genscan_6
	JGI N. vectensis v1.0 assembly (genscan result)	scaffold_402_genscan_9

	JGI N. vectensis v1.0 assembly (genscan result)	scaffold_402_genscan_4
	JGI N. vectensis v1.0 assembly (genscan result)	scaffold_405_genscan_5
	JGI N. vectensis v1.0 assembly (genscan result)	scaffold_41_genscan_3
	JGI N. vectensis v1.0 assembly (genscan result)	scaffold_41_genscan_7
	JGI N. vectensis v1.0 assembly (genscan result)	scaffold_41_genscan_57
	JGI N. vectensis v1.0 assembly (genscan result)	scaffold_417_genscan_2
	JGI N. vectensis v1.0 assembly (genscan result)	scaffold_417_genscan_4
	JGI N. vectensis v1.0 assembly (genscan result)	scaffold_417_genscan_9
	JGI N. vectensis v1.0 assembly (genscan result)	scaffold_417_genscan_14
	JGI N. vectensis v1.0 assembly (genscan result)	scaffold_474_genscan_7
	JGI N. vectensis v1.0 assembly (genscan result)	scaffold_474_genscan_13
	JGI N. vectensis v1.0 assembly (genscan result)	scaffold_474_genscan_10
	JGI N. vectensis v1.0 assembly (genscan result)	scaffold_50_genscan_66
	JGI N. vectensis v1.0 assembly (genscan result)	scaffold_6_genscan_255
	JGI N. vectensis v1.0 assembly (genscan result)	scaffold_70_genscan_97
	JGI N. vectensis v1.0 assembly (genscan result)	scaffold_70_genscan_98
	JGI N. vectensis v1.0 assembly (genscan result)	scaffold_91_genscan_12
	JGI N. vectensis v1.0 assembly (genscan result)	scaffold_91_genscan_13
N. vectensis (sea anemone)	JGI *N. vectensis* v1.0 annotation	estExt_fgenesh1_pg.C_11630001
	JGI *N. vectensis* v1.0 annotation	estExt_fgenesh1_pg.C_3190006
	JGI *N. vectensis* v1.0 annotation	estExt_fgenesh1_pg.C_320002
	JGI *N. vectensis* v1.0 annotation	estExt_fgenesh1_pg.C_320006
	JGI *N. vectensis* v1.0 annotation	estExt_fgenesh1_pg.C_700066
	JGI *N. vectensis* v1.0 annotation	fgenesh1_pg.scaffold_192000040
	JGI *N. vectensis* v1.0 annotation	fgenesh1_pg.scaffold_291000006
	JGI *N. vectensis* v1.0 annotation	fgenesh1_pg.scaffold_405000006
	JGI *N. vectensis* v1.0 annotation	fgenesh1_pg.scaffold_474000013

To get a more detailed view of NACHT domain containing sequences in the selected species, different protein databases were checked and all sequences that could be confirmed by Pfam Protein Search or NCBI Conserved Domain Search under the default threshold were listed above.
