# Supplementary material for: Novel genes dramatically alter regulatory network topology in amphioxus
Source: Genome Biol. 2008 Aug 4;9(8):R123. doi: 10.1186/gb-2008-9-8-r123 (PMC2575513; doi:10.1186/gb-2008-9-8-r123)
Supplement: Additional data file 3 — SRCR domain combinations in different genomes. [file gb-2008-9-8-r123-S3.pdf]

Additional Table 3. List of SRCR domain combinations in different genomes

Genome	SRCR Domain Containing Sequence	SRCR Domain Combinations	SRCR Partner Domains*
N. vectensis	66	8	MAM, Ldl_recept_a, CUB, EGF_CA, Trypsin, GLTT, LRR_1, ig
C. elegans	1	0	————
D. melanogaster	4	5	Lysyl_oxidase, Lectin_C, CBM_14, Ldl_recept_a, Trypsin
Sea urchin	269	32	CUB, Exo_endo_phos, Kringle, F5_F8_type_C, EGF, GPS, 7tm_2, Sushi, Ldl_recept_b, Zona_pellucida, ShK, LRR_1, 7tm_1, Y_phosphatase, RVT_1, rve, WSC, HYR, V-set, Tim17, ig, DUF1891, HSP70, TIL, Somatomedin_B, MAM, Fibrinogen_C, PAN_1, zf-C2HC, zf-B_box, I-set, EGF_CA
Amphioxus	229	56	MACPF, F5_F8_type_C, PAN_1, Lectin_C, EGF, Sushi, CUB, MAM, Kringle, Zona_pellucida, U-box, TSP_1, I-set, Arf, IF4E, PALP, Lysyl_oxidase, T4_deiodinase, Furin-like, WD40, Fz, Gal_Lectin, Pentaxin, TNF, Trefoil, fn3, GPS, PKD_channel, PSI, VWD, EGF_2, Cadherin, EGF_CA, Gram_pos_anchor, WSC, GON, Ldl_recept_a, Prion_octapep, Collagen, Fibrinogen_C, VWA, Trypsin, Neur_chan_LBD, Neur_chan_memb, PFK, Cu-oxidase_3, Chitin_synth_2, OTU, SAM_2, GCC2_GCC3, C1q, TIL, DOMON, SEA, NIDO, CBM_14
Ciona	5	4	Trypsin, Lysyl_oxidase, Sushi, EGF
Zebrafish	34	6	Collagen, Kringle, Trypsin, Lysyl_oxidase, Ldl_recept_a, BACK
Tetraodon	22	5	Trypsin, SH3BGR, Lysyl_oxidase, EGF_CA, Collagen
Fugu	16	5	Kringle, Trypsin, Lysyl_oxidase, BACK, Zona_pellucida
Xenopus	25	4	Trypsin, Kringle, Lysyl_oxidase, Collagen
Chicken	41	10	Lysyl_oxidase, Kringle, Trypsin, CHCH, CUB, Zona_pellucida, Collagen, SEA, Ldl_recept_a, MAM
Dog	28	10	Collagen, BACK, Macscav_rec, SEA, CUB, MAM, Ldl_recept_a, Trypsin, Lysyl_oxidase, ig
Mouse	27	12	Ldl_recept_a, Trypsin, Lysyl_oxidase, CUB, Zona_pellucida, Collagen, SEA, MAM, Macscav_rec, Kringle, BACK, ig
Human	23	12	Kazal_2, Ldl_recept_a, Trypsin, CUB, Zona_pellucida, SEA, MAM, BACK, Lysyl_oxidase, Collagen, Macscav_rec, Kringle

*Although SRCR domain itself was not counted in both 'SRCR Domain Combinations' and 'SRCR Partner Domains' column, tandem repeats of SRCR domains do exist in all above genomes.
