# Supplementary material for: Novel genes dramatically alter regulatory network topology in amphioxus
Source: Genome Biol. 2008 Aug 4;9(8):R123. doi: 10.1186/gb-2008-9-8-r123 (PMC2575513; doi:10.1186/gb-2008-9-8-r123)
Supplement: Additional data file 4 — Partner domains that combine with individual TIR or NACHT domains in different genomes. [file gb-2008-9-8-r123-S4.pdf]

Additional Table 4. List of partner domains that combine with individual TIR or NACHT domains in each designated genome

Genome	TIR Domain Combinations	TIR Partner Domains	NACHT Domain Combinations	NACHT Partner Domains
N. vectensis	4	Death, ig, I-set, V-set	3	DED, A2M_N, Phospholip_A2_1
C. elegans	3	LRR_1, SAM_1, SAM_2	0	—
D. melanogaster	5	LRRNT, LRRCT, LRR_1, SAM_2, SAM_1	0	—
Sea urchin	6	LRR_1, LRRCT, PID, SAM_2, SAM_1, Death	15	Death, GPS, 7tm_2, LRR_2, MFS_1, SRP54, Sterol_desat, Lectin_C, KH_1, EGF, EGF_2, Sulfatase, RVT_1, Fibrinogen_C, ZU5
Amphioxus	28	LRR_1, LRRCT, LRRNT, EGF, HMG_box, TPR_1, TPR_2, Death, NACHT, NB-ARC, WD40, V-set, ig, I-set, DUF6, Pkinase, Pkinase_Tyr, SAM_2, SAM_1, HEAT_PBS, HEAT, CARD, Glycos_transf_1, Laminin_N, Laminin_EGF, Laminin_II, Laminin_G_2, Laminin_G_1	21	F-box, WD40, Death, LRR_1, Autophagy_N, Autophagy_C, RNA_pol_Rpc4, CARD, V-set, ig, I-set, SH3_1, SH3_2, DED, TIR, OKR_DC_1, F5_F8_type_C, Gal_Lectin, Myb_DNA-binding, zf-C3HC4, LRR_2
Ciona	1	LRR_1	0	—
Zebrafish	2	LRR_1, Death	3	CARD, LRR_1, PAAD_DAPIN
Tetraodon	8	LRR_1, LRR_2, SAM_1, SAM_2, LRRCT, Death, C2, ig	4	PAAD_DAPIN, zf-C3HC4, CARD, Filament
Fugu	7	ig, V-set, SAM_1, SAM_2, LRR_1, LRRCT, Death	4	LRR_1, Filament, CARD, PAAD_DAPIN
Xenopus	4	LRR_1, LRRCT, ig, Death	3	LRR_1, LRR_2, CARD
Chicken	7	LRR_1, SAM_2, SAM_1, Death, ig, I-set, V-set	3	LRR_1, PAAD_DAPIN, CARD
Dog	6	ig, Death, LRR_1, LRRCT, SAM_2, SAM_1	5	PAAD_DAPIN, LRR_1, CARD, Tropomodulin, LRR_2
Mouse	8	ig, LRRCT, LRR_1, Death, SAM_2, SAM_1, V-set, I-set	5	LRR_1, BIR, CARD, PAAD_DAPIN, LRR_2
Human	8	ig, V-set, I-set, LRR_1, LRRCT, Death, SAM_2, SAM_1	4	PAAD_DAPIN, CARD, LRR_1, BIR
