# Supplementary material for: Novel genes dramatically alter regulatory network topology in amphioxus
Source: Genome Biol. 2008 Aug 4;9(8):R123. doi: 10.1186/gb-2008-9-8-r123 (PMC2575513; doi:10.1186/gb-2008-9-8-r123)
Supplement: Additional data file 5 — Selected JGI-predicted amphioxus gene models that have been verified by RT-PCR. [file gb-2008-9-8-r123-S5.pdf]

Additional Table 5. Verification of JGI-predicted amphioxus gene models by cDNA sequencing

GenBank ID	JGI Protein ID (Name)	Domain combination*	Size of JGI-predicted model	Region spanned by cDNA**
EU049585	Brafl1_82667 (fgenesh2_pg.scaffold_114000007)	TNFr—Caspase	1431bp; 476aa	224-1073bp
EU049586	Brafl1_82667 (fgenesh2_pg.scaffold_114000007)	TNFr—Caspase	1431bp; 476aa	262-877bp
EU049591	Brafl1_105741 (fgenesh2_pg.scaffold_505000014)	Death—Caspase	1320bp; 439aa	210-1128bp
EU049592	Brafl1_82459 (fgenesh2_pg.scaffold_111000114)	Death—NACHT—LRRs	3342bp; 1113aa	395-2180bp
EU049593	Brafl1_89453 (fgenesh2_pg.scaffold_187000018)	Death—NACHT—LRRs	3333bp; 1110aa	166-1307bp
EU049594	Brafl1_89451 (fgenesh2_pg.scaffold_187000016)	Death—NACHT—LRRs	3615bp; 1204aa	949-2708bp
EU049589	Brafl1_98233 (fgenesh2_pg.scaffold_317000043)	DED—NACHT	2067bp; 688aa	234-1945bp
EU049596	Brafl1_68590 (fgenesh2_pg.scaffold_19000170)	TIR—[NB-ARC]—WD40s	5076bp; 1691aa	1108-3692bp
EU049587	Brafl1_94467 (fgenesh2_pg.scaffold_253000051)	CARD—NACHT	3156bp; 1051aa	426-1126bp
EU049595	Brafl1_92153 (fgenesh2_pg.scaffold_219000011)	CARD—NACHT—LRRs	2994bp; 997aa	92-1843bp
EU049588	Brafl1_194931 (gw.97.227.1)	DED—DED—Caspase	1428bp; 476aa	195-1028bp
EU049590	Brafl1_246353 (e_gw.470.10.1)	Death—TIR	897bp; 298aa	210-676bp
EU049584	Brafl1_95677 (fgenesh2_pg.scaffold_272000001)	SAM—TIR	1367bp; 455aa	346-1275bp
EU049583	Brafl1_131196 (estExt_fgenesh2_pg.C_5050026)	TIR—SAM	2145bp; 714aa	454-1022bp
EU279424	Brafl1_244359 (e_gw.440.22.1)	CARD—Caspase	1083bp; 360aa	36-711bp
EU279425	Brafl1_88143 (fgenesh2_pg.scaffold_170000017)	TIR—Laminin	4041bp;1346aa	425-1075bp

Selected JGI-predicted amphioxus gene models (including shortcut proteins) were verified by RT-PCR. Models are considered “proven” when cDNAs were recovered that spanned the domain combinations in question. Some variations between the cloned sequences and related JGI models could be the result of haplotype variation and alternative splicing.

*Domain combinations are colored in red to illustrate the approximate region confirmed (proportion of model).

**Region spanned by cDNA is an ongoing project, which includes the characterization of alternative transcripts, and will be maintained as a more thorough and updated table on our website at [61].
