# Supplementary material for: Novel genes dramatically alter regulatory network topology in amphioxus
Source: Genome Biol. 2008 Aug 4;9(8):R123. doi: 10.1186/gb-2008-9-8-r123 (PMC2575513; doi:10.1186/gb-2008-9-8-r123)
Supplement: Additional data file 6 — Examples of novel domain combinations in amphioxus that represent the shortcuts between two or more proteins present in human. [file gb-2008-9-8-r123-S6.doc]

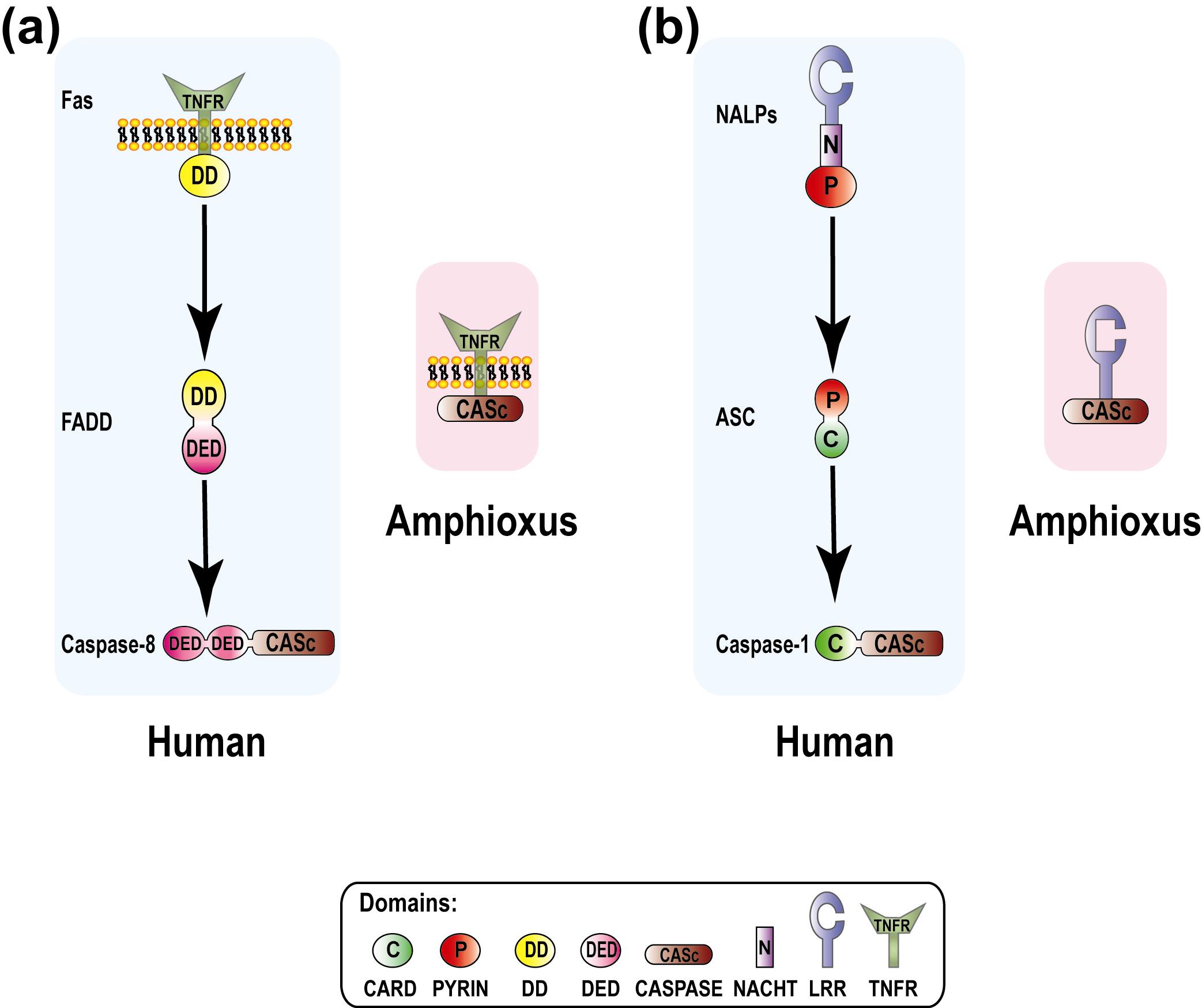


**Additional data file 6.** Examples of novel domain combinations in amphioxus represent the shortcuts between two or more proteins present in human. In both **(**a**)** Fas-induced apoptosis and **(**b**)** NALPs signaling pathways, amphioxus (shown against a pink background) has proteins with unusual domain architectures that contain both upstream receptor and downstream activator domains, which could bypass the usual signal transduction pathways in human (shown against a blue background).
