# Supplementary material for: Novel genes dramatically alter regulatory network topology in amphioxus
Source: Genome Biol. 2008 Aug 4;9(8):R123. doi: 10.1186/gb-2008-9-8-r123 (PMC2575513; doi:10.1186/gb-2008-9-8-r123)
Supplement: Additional data file 7 — Alignment of sequences in the vicinity of the catalytic center of the caspase domain from human caspases and amphioxus proteins with TNFR-caspase or LRRs-caspase architectures. [file gb-2008-9-8-r123-S7.doc]

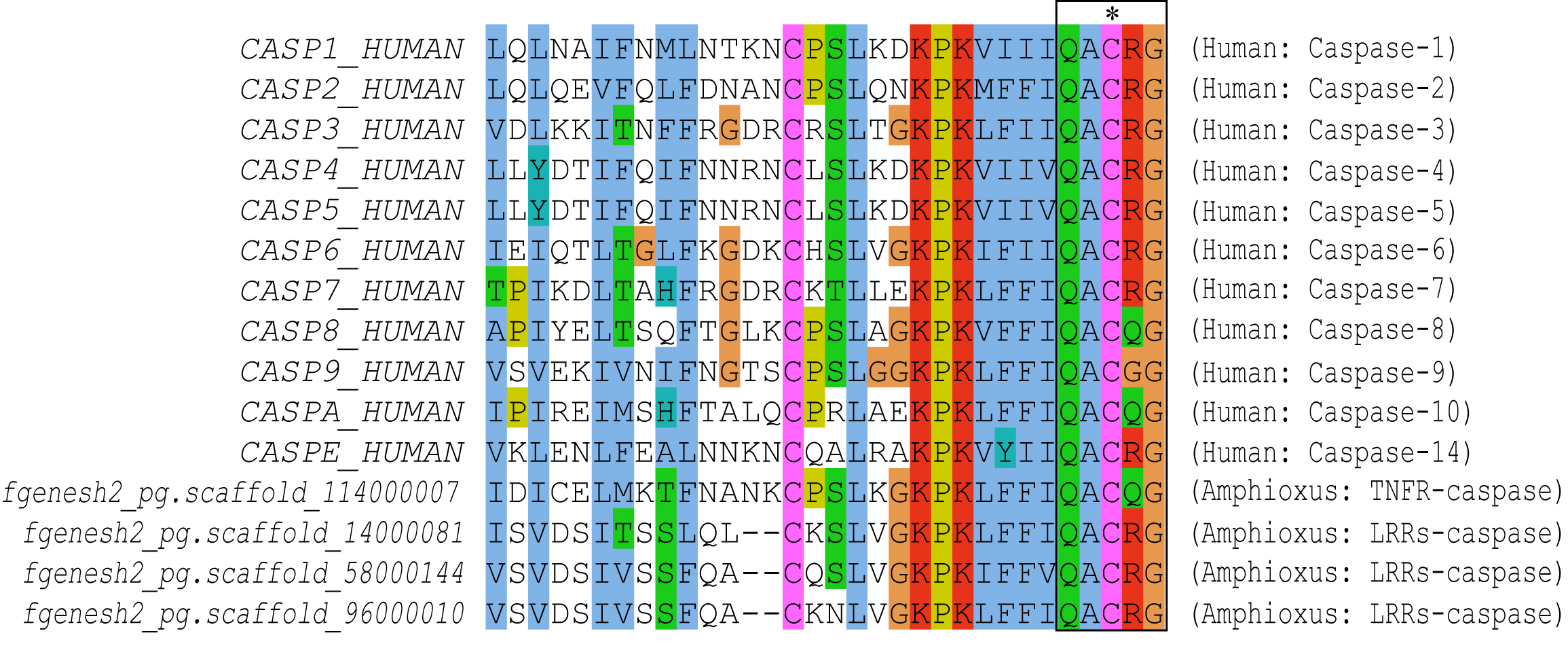


**Additional data file 7.** Alignment of sequences in the vicinity of the catalytic center of the caspase domain from human caspases and amphioxus proteins with TNFR-caspase or LRRs-caspase architectures. The catalytic center is boxed and the active-site cysteine residue is highlighted by ‘*’.
